# M(IV)- and M(V)-Substituted {MW_5_} Lindqvist-Type Polyoxometalates from Plenary [W_6_O_19_]^2–^ or WO_3_·H_2_O: A Gateway to Wider Systematic Reactivity Studies

**DOI:** 10.1021/acs.inorgchem.6c03884

**Published:** 2026-10-14

**Authors:** Dominic Shiels, Daniel Lebbie, Amar Mohammed, Magda Pascual-Borràs, Rosie Black, Corinne Wills, Paul G. Waddell, Benjamin R. Horrocks, R. John Errington

**Affiliations:** † NUPOM Lab, Chemistry, School of Natural and Environmental Sciences, 5994 Newcastle University, Newcastle upon Tyne NE1 7RU, U.K.; ‡ Departament de Química Inorgànica i Orgànica, Institut de Química Teòrica i Computacional, Universitat de Barcelona, Barcelona 08028, Spain; ○ Chemistry, School of Natural and Environmental Sciences, Newcastle University, Newcastle upon Tyne NE1 7RU, U.K.

## Abstract

The reactivity of polyoxometalates (POMs) may be tuned by heterometal substitution into the POM framework, and systematic reactivity studies of these nanoscale molecular metal oxide structures require reliable, efficient synthetic methods. We have now shown that nonaqueous base degradation of either WO_3_·H_2_O or plenary (TBA)_2_[W_6_O_19_] (TBA)_2_ **1** followed by reaction with heterometal precursors provides convenient, high-yield access to M^IV^- and M^V^-substituted {MW_5_} Lindqvist-type hexametalates [(^i^PrO)­TiW_5_O_18_]^3–^ **2**, [(MeO)­MW_5_O_18_]^3–^ (M = Ti **3**, Sn **4**), [{(MeO)­MW_5_O_18_}_2_]^6–^ (M = Zr **5**, Hf **6**) and [MW_5_O_19_]^3–^ (M = V **7**, Nb **8**, Ta **9**). Crystal structures of (TBA)_6_ **5** and (TBA)_6_ **6** showed the anions to be isostructural, and kinetic studies of CD_3_OD exchange revealed slightly slower exchange at Hf than Zr. For **5**, DFT analysis of MeOH exchange revealed a transition state involving a six-membered ring via insertion of MeOH into a Zr­(μ-OMe)Zr bridge. Alkoxide protonolysis reactions provided monomeric {LMW_5_} derivatives with 7-coordinate Zr or Hf, and X-ray crystal structures of (TBA)_3_[(PhCO_2_)­ZrW_5_O_18_] (TBA)_3_ **12**, (TBA)_3_[(PhCO_2_)­HfW_5_O_18_] (TBA)_3_ **13** and (TBA)_3_[(acac)­HfW_5_O_18_] (TBA)_3_ **14** were obtained. Cyclic voltammetry showed one-electron couples for **3**, **4**, **7**, **8,** and **9** but not for dimeric **5** and **6**, consistent with calculated reduction potentials for these {MW_5_} anions.

## Graphical abstract

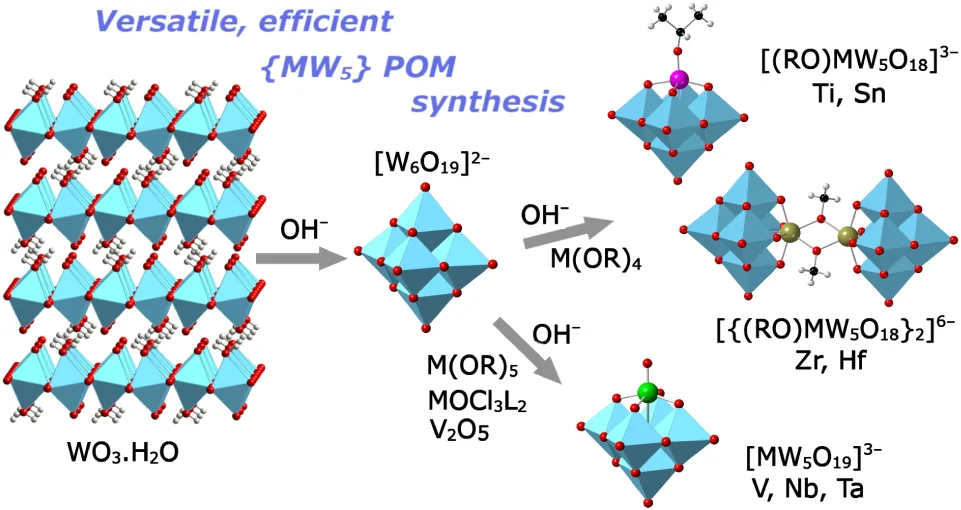

## Introduction

Polyoxometalates (POMs) of the group 5 and group 6 metals V, Nb, Ta, Mo and W are molecular metal oxides occupying the chemical landscape between discrete monometallic complexes and extended solid oxides, and their remarkably versatile structural and electronic properties continue to attract widespread interest from theoretical and applications perspectives.^1^ One particular feature of POMs is that one or more of the addenda metals in the metal oxide framework may be substituted by reactive heterometals, and such species provide well-defined models of doped metal oxide catalysts for detailed studies of electronic properties and solution reactivity. Following Pope’s seminal overview,^1a^ POM research expanded rapidly during the 1980’s and included ventures into nonaqueous chemistry that opened up exciting new prospects for POM reactivity.^2^ During these studies, it was evident that tungstate speciation in organic solvents is different from that in water, and the tetrabutylammonium hexatungstate salt (TBA)_2_[W_6_O_19_] (TBA)_2_ **1**, prepared by acidification of mixed aqueous/organic solutions of Na_2_WO_4_,^3^ was established as a paradigm for nonaqueous polyoxotungstate chemistry. The plenary [W_6_O_19_]^2–^ anion **1** is generally rather inert, but substitution of {W=O}^4+^ to give [XMW_5_O_18_]^n–^ derivatives enables rational tuning of the charge on the anion, the Lewis acid and redox properties of M, the reactivity of M–X and the basicity of MOW oxygens, thereby providing a versatile template for systematic reactivity studies. As part of a program to fully evaluate the reactivities of these “single-site” {MW_5_} molecular metal oxides, we have developed reliable and scalable synthetic routes to these derivatives and describe here efficient and versatile methodologies involving the base degradation of (TBA)_2_ **1** or WO_3_·H_2_O in organic solvents (Scheme 1).

**Scheme 1:**
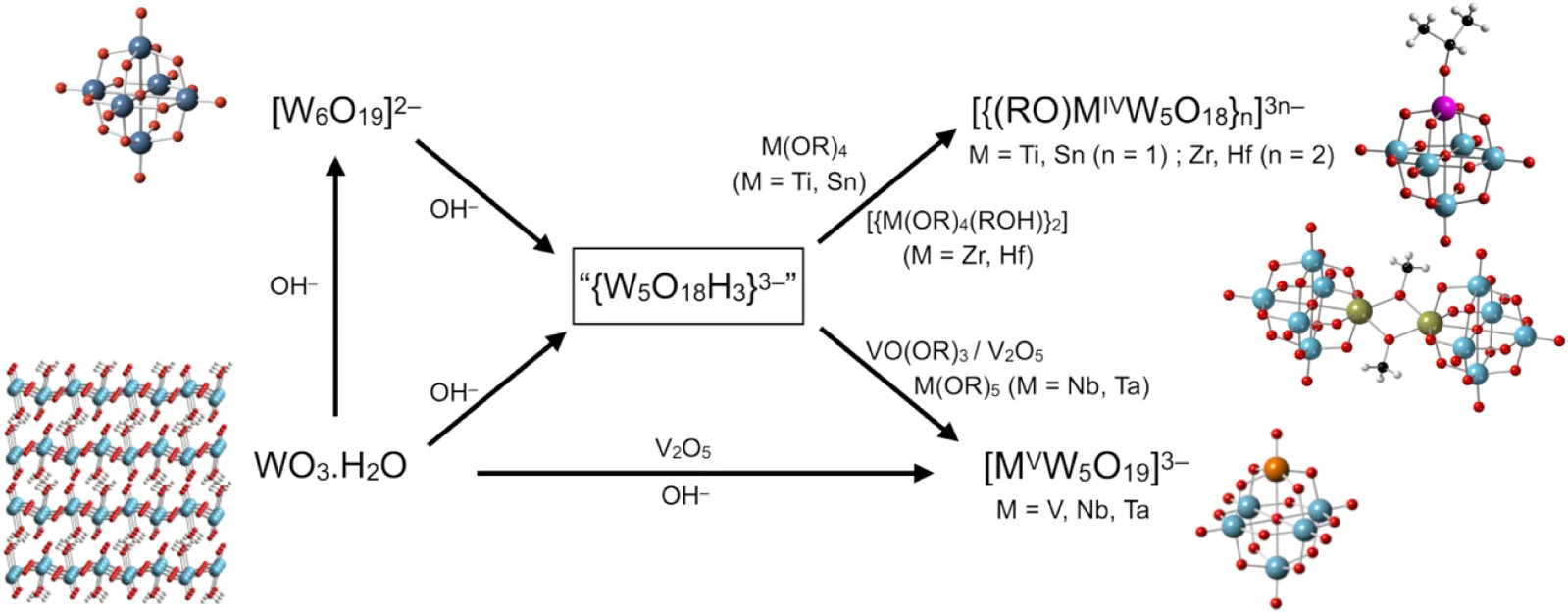
Conversion of WO_3_·H_2_O and [W_6_O_19_]^2–^ to M^IV^ and M^V^ Substituted {MW_5_} Lindqvist Anions

Our initial foray into the nonaqueous chemistry of Lindqvist anions involved the hydrolytic aggregation of metal alkoxides in the presence of (TBA)_2_[WO_4_], which provided access not only to ^17^O-enriched (TBA)_2_ **1** but also to a range of alkoxido-substituted M^IV^ derivatives [(RO)­MW_5_O_18_]^3–^ (M = Ti, Sn) and [{(μ-OR)­MW_5_O_18_}_2_]^6–^ (M = Zr, Hf) as well as [MW_5_O_19_]^3–^ (M = V, Nb, Ta) as TBA^+^ salts.^4^ This access to reactive {MW_5_} derivatives led to the first covalent attachment of {MW_5_} units to the surface of silicon^5^ and detailed investigations of reactivity.^6^ By modifying this hydrolytic strategy, we subsequently prepared the {Co^II^W_5_} derivative (TBA)_6_[(CoW_5_O_18_H)_2_] by generating a solution containing a “virtual” {W_5_} lacunary precursor.^7^ While this work successfully established access to a series of reactive {MW_5_} Lindqvist anions, we sought a more convenient synthesis methodology for scale-up and extensive reactivity studies. By analogy with the preparation of {ML}^x+^ substituted [(L)­MEW_11_O_39_]^(n+4–x)–^ Keggin species from lacunary [EW_11_O_39_]^(n+4)–^ (where E is a central heteroatom such as Al, Si, P *etc*.) we turned our attention to the generation of the lacunary {W_5_} species. It is worth noting that lacunary Keggin anions are readily prepared as solution intermediates or as isolated protonated or alkali metal derivatives [MEW_11_O_39_]^(n+3)–^ (where M occupies the “vacant” site in the lacunary structure),^2d,8^ but the lacunary Lindqvist anion [W_5_O_18_]^6–^ has not been isolated for use as a precursor to {MW_5_} species, although the {W_5_O_18_} structural unit has been observed as structural components of larger polytungstates.^9^ The complete removal of one {WO}^4+^ from a plenary Keggin structure upon treatment with base can be represented by eq 1 and the analogous reaction for the plenary Lindqvist structure by eq 2.

1 $${[{\mathrm{EW}}_{12}O_{40}]}^{n-}{+6\mathrm{OH}}^{-}\to {[{\mathrm{EW}}_{11}O_{39}]}^{\left(n+4\right)-}{{+\mathrm{WO}}_{4}}^{2-}{+3H}_{2}O$$

2 $${[W_{6}O_{19}]}^{2-}{+6\mathrm{OH}}^{-}\to {[W_{5}O_{18}]}^{6-}{{+\mathrm{WO}}_{4}}^{2-}{+3H}_{2}O$$

Our recent characterization of the new isopolytungstate [W_7_O_24_H]^5–^ containing the {W_5_O_18_} structural unit demonstrated that the lacunary {W_5_} fragment may be generated in nonaqueous solutions of tungstates [W_*x*_O_*y*_H_*z*_]^n–^ by manipulating the anionic charge density per W atom in the system (i.e., n/x) and, importantly, that the {W_5_} unit is present at lower values of n/x than the stoichiometry indicated in eq 2.^10^ In order to synthesize [XMW_5_O_18_]^3–^ species from **1** we reasoned that, by adjusting n/x to 0.6, we might generate species equivalent to the “virtual” lacunary [W_5_O_18_H_3_]^3–^ for reaction with M^IV^(OR)_4_ alkoxide or possibly oxide (e.g., V_2_O_5_) heterometal precursors. Note that this n/x value was achieved in our hydrolytic approach by adjusting the relative amounts of (TBA)_2_[WO_4_] and WO­(OMe)_4_. We now report that nonaqueous manipulation of n/x, achieved by base degradation of either (TBA)_2_ **1** or polymeric WO_3_·H_2_O with (TBA)­OH, provides convenient access to substituted {MW_5_} Lindqvist-type anions.

## Experimental Section

### General Experimental Details

The Supporting Information provides details of general procedures, spectroscopy, electrochemistry, X-ray crystallography and computation. **Caution!** Extreme care should be taken both in the handling of the cryogen liquid nitrogen and its use in the Schlenk line trap to avoid the condensation of oxygen from air.

### Syntheses

#### (TBA)_2_[W_6_O_19_] (TBA)_2_ **1**

Removal of the volatiles from methanolic (TBA)­OH (1 *M*, 27.0 mL, 27.0 mmol) gave an oil which was dissolved in DMF (15 mL). WO_3_·H_2_O (20.0 g, 80.0 mmol) was added and the resulting yellow suspension was heated with stirring. At 160 °C the suspension became much paler and after heating at 180 °C for 10–15 min gave a slightly hazy, faintly blue solution, which was allowed to cool. Addition of diethyl ether (160 mL) with vigorous stirring gave a precipitate which was filtered, pumped dry and dissolved in MeCN (45 mL) with heating. The crystals formed by cooling the filtered solution were isolated by filtration and pumped dry. The filtrate was reduced in volume and cooled in the freezer overnight to give a second crop of crystals which was separated from the mother liquor by cannulation and pumped dry. The ^183^W NMR spectrum of a saturated solution in hot *d*_6_-DMSO contained a single peak at 63.7 ppm (total yield 23.4 g, 93%).

#### (TBA)_3_[(^I^PrO)­TiW_5_O_18_] (TBA)_3_ **2**

After removal of the volatiles from methanolic (TBA)­OH (1*M*, 11.8 mL, 11.8 mmol), the oily residue was dissolved in MeCN (10 mL) and the solution pumped dry. This process was repeated, the residue dissolved in MeCN (20 mL) and (TBA)_2_ **1** (14.00 g, 7.40 mmol) added with stirring and gentle warming until the solution was clear (∼30 min). ^17^O-enriched H_2_O (40% enrichment, 133 μL, 7.38 mmol) was then added to the solution, which was stirred overnight. After removal of the volatiles under vacuum, the residue was dissolved in MeCN (30 mL) and Ti­(O^i^Pr)_4_ (2.7 mL, 8.88 mmol) was added with stirring and the mixture was heated at 50 °C overnight. Volatiles were removed under vacuum and the residue was triturated with hexanes (20 mL) then washed with toluene (20 mL) and diethyl ether (20 mL) and pumped dry to give a crystalline solid (14.0 g, 77%), which contained a small amount of a hydrolysis product that could be removed by crystallization. NMR spectra were consistent with previously reported data.^6a^

#### (TBA)_3_[(MeO)­TiW_5_O_18_], (TBA)_3_ **3**

After removal of the volatiles from methanolic (TBA)­OH (1*M*, 8.5 mL, 8.5 mmol), the oily residue was dissolved in MeCN (5 mL) and the solution pumped dry. The residue was dissolved in MeCN (25 mL) and (TBA)_2_ **1** (10.00 g, 5.29 mmol) added with stirring and gentle warming for ca.1 h to give a slightly hazy solution. ^17^O-enriched H_2_O (40% enrichment, 95 μL, 5.3 mmol) was then added to the solution, which was stirred for 30 min before addition of Ti­(O^i^Pr)_4_ (1.93 mL, 6.36 mmol). After stirring at 50 °C for 20 h, volatiles were removed from the very slightly hazy solution under vacuum and the residual sticky solid was triturated with toluene (15, 10, and 10 mL), pumped dry and then dissolved in MeCN (10 mL) with warming. MeOH (1 mL) was added, the solution stirred at 80 °C for 15 min, then at 50 °C for 30 min and allowed to cool. Addition of diethyl ether (80 mL) gave a solid which, after the separation of the supernatant liquid, was washed with diethyl ether (30 mL) and dried *in vacuo* to give 12.00 g of crude product. Small amounts of impurities were removed by recrystallization from MeCN/diethyl ether. NMR spectra were consistent with previously reported data.^4a^

#### (TBA)_3_[(MeO)­SnW_5_O_18_] (TBA)_3_ **4**

The residue after removal of the volatiles from methanolic (TBA)­OH (17.0 mL, 17.0 mmol), was dissolved in MeCN (10 mL) and the resulting solution was then pumped dry. This process was repeated and the (TBA)­OH was dissolved in MeCN (40 mL). (TBA)_2_ **1** (20.0 g, 10.6 mmol) was added to this solution with stirring to give a cloudy solution, followed by addition of ^17^O-enriched H_2_O (40% enrichment, 191 μL, 10.4 mmol) and stirring overnight. Sn­(O^t^Bu)_4_ (5.23 g, 12.72 mmol) was added to give a cloudy solution which was heated overnight at 40 °C. A ^119^Sn NMR spectrum of the resulting solution was recorded, which showed a major peak at −648 ppm in addition to minor impurity peaks at −614 and −665 ppm. After removal of the volatiles, the residue was pumped dry and triturated with toluene and then diethyl ether to afford a white solid, which was suspended in MeOH (40 mL) and stirred at 85 °C overnight. Removal of the volatiles and recrystallization of the solid residue from MeCN gave two fractions with ^119^Sn NMR spectra containing a single peak at −649 ppm for (TBA)_3_ **4** and ^17^O NMR spectra that were as previously reported^4b,6c^ and contained no peaks due to (TBA)_2_ **1**. Overall yield 18.9 g, 71%.

#### (TBA)_6_[{(MeO)­ZrW_5_O_18_}_2_] (TBA)_6_ **5**

The residue after removal of the volatiles from methanolic (TBA)­OH (0.93 *M*, 4.7 mL, 4.33 mmol) was dissolved in MeCN (10 mL) and the resulting solution was then pumped dry. This process was repeated and the (TBA)­OH was dissolved in MeCN (30 mL). (TBA)_2_ **1** (5.13 g, 2.71 mmol) was added with stirring until the solid had dissolved. The solution was transferred via cannula onto [{Zr­(^i^PrO)_4_(^i^PrOH)}_2_] (1.26 g, 1.63 mmol) and the mixture was stirred until the solution was clear before addition of ^17^O-enriched H_2_O (49 μL, 2.71 mmol). After stirring overnight, the solution was pumped dry and the remaining solid was triturated with toluene and diethyl ether to give a white powder (6.73 g), which was stirred in MeOH (50 mL) overnight. Ether was added to the resulting solution and cooling to −30 °C in the freezer gave 2.77 g of colorless crystals. A further 1.50 g was obtained from the mother liquor to give an overall yield of 64%. NMR spectra were consistent with previously reported data.^6b^

#### (TBA)_6_[{(MeO)­HfW_5_O_18_}_2_] (TBA)_6_ **6**

The residue after removal of the volatiles from methanolic (TBA)­OH (1.0 *M*, 8.0 mL, 8.0 mmol), was dissolved in MeCN (15 mL) and the resulting solution was then pumped dry. This process was repeated and the (TBA)­OH was dissolved in MeCN (40 mL). (TBA)_2_ **1** (9.48 g, 5.01 mmol) was added and the mixture was stirred until the solid had dissolved. Addition of ^17^O-enriched H_2_O (90 μL, 5.01 mmol) gave a clear solution that was stirred for 15 min before addition of [{Hf­(^i^PrO)_4_(^i^PrOH)}_2_] (2.85 g, 3.00 mmol). After stirring overnight, volatiles were removed under reduced pressure and the residue triturated with diethyl ether (40 mL). The residual solid was dried in vacuo, dissolved in the minimum of MeOH and placed in the freezer overnight. The resulting solid was isolated by filtration and pumped dry (6.93 g). Volatiles were removed from the filtrate and the residue was dissolved in a mixture of MeCN (5 mL) and MeOH (2 mL) with stirring. After 1 h, diethyl ether (40 mL) was added to give a precipitate which was filtered, recrystallized from the minimum amount of MeOH and pumped dry (4.33 g). Total yield 11.26 g, 86%. ^1^H NMR (300.13 MHz, CD_3_CN) δ (ppm): 3.84 (OC*H*_3_). ^13^C NMR (75.48 MHz, CD_3_CN) δ (ppm): 58.0 (O*C*H_3_). ^17^O NMR (40.7 MHz, CD_3_CN) δ (ppm): 712, 690 (W=O), 464 (HfOW), 387, 379 (WOW), −61 (μ_6_-O). ^183^W NMR (20.8 MHz, CD_3_CN) δ (ppm): 54.7 (4W, W_eq_), 68.7 (1W, W_ax_). *v*_max_/cm^–1^ = 2959, 2931, 2871, 1482, 1379, 1152, 1107, 1055, 1026, 967 (w), 943 (vs), 884, 790 (vs), 632, 561, 498, 438, 413 (vs).

#### (TBA)_3_[VW_5_O_19_] (TBA)_3_ **7**

##### Method A – From (TBA)_2_ **1** and VO­(O^t^Bu)_3_

Volatiles were removed from methanolic (TBA)­OH (1 *M*, 1.7 mL, 1.7 mmol) to give an oil, which was dissolved in MeCN (2–3 mL) and then the solvent was removed again. This process was repeated twice more to ensure removal of the MeOH. The oil was dissolved in MeCN (20 mL) and (TBA)_2_ **1** (2.0 g, 1.1 mmol) was added. The suspension was stirred at room temperature for 60–90 min or until all the solid had dissolved. ^17^O-enriched H_2_O (40% enrichment, 19.1 μL, 1.1 mmol) was added and the solution was stirred overnight. The mixture was transferred via cannula to a separate flask containing VO­(O^t^Bu)_3_ (0.36 g, 1.3 mmol) to give a yellow solution which was stirred at room temperature overnight. Volatiles were removed, and the remaining yellow solid was dissolved in the minimum amount of hot MeCN and cooled to −30 °C in a freezer overnight. Crystals were isolated by vacuum filtration, washed with diethyl ether (2 × 20 mL) and dried under vacuum overnight at 50 °C (2.1 g, 83% yield). (Note: solid can also be easily recrystallized by slow evaporation of saturated DCM solutions). ^17^O NMR (40.7 MHz, CD_3_CN) δ (ppm): 1218 (V=O), 733 (W=O), 564 (VOW), 398, 391 (WOW), −73 (μ_6_-O). ^51^V NMR (29.9 MHz, CD_3_CN) δ (ppm): −508. ^183^W NMR (20.8 MHz, *d*_6_-DMSO) δ (ppm): 78.4 (broad), 79.6 (broad). *v*_max_/cm^–1^ = 2958, 2872, 1481, 1381, 1151, 1107, 1055, 1026, 989, 963, 949 (vs), 883, 788 (vs), 736, 581, 436 (vs).

##### Method B – From (TBA)_2_ **1** and V_2_O_5_

Volatiles were removed from methanolic (TBA)­OH (1 *M*, 1.7 mL, 1.7 mmol) before addition of DMF (3 mL) and (TBA)_2_ **1** (2.0 g, 1.1 mmol). The suspension was stirred at room temperature for 10 min to dissolve the crystalline solid and ^17^O-enriched H_2_O (40% enrichment, 19 μL, 1.1 mmol) was added with stirring for a further 10 min. After addition of V_2_O_5_ (0.115 g, 0.63 mmol), the stirred colorless solution slowly became pale yellow at room temperature and after heating at 180 °C for 15 min gave an orange solution containing some suspended yellow crystals. ^51^V NMR of the hot reaction mixture showed a single peak with a temperature-dependent chemical shift. The solution became paler yellow upon cooling with the formation of yellow crystals. The addition of diethyl ether (50 mL) with stirring gave further yellow crystalline product, which was filtered, washed with diethyl ether (2 × 20 mL) and dried under vacuum. Yield 2.23 g, 88%.

##### Method C – From WO_3_·H_2_O and V_2_O_5_

Volatiles were removed from methanolic (TBA)­OH (1 *M*, 7.5 mL, 7.5 mmol) before addition of DMF (5 mL), followed by WO_3_·H_2_O (3.12 g, 12.5 mmol) and V_2_O_5_ (0.23 g, 1.25 mmol). The stirred mixture was heated at 180 °C for 35 min to give an orange-yellow solution, which crystallized upon cooling. After addition of diethyl ether (120 mL) with stirring, the yellow crystals were filtered, washed with diethyl ether (20 mL) and pumped dry (4.42 g, 88%).

##### Method D – From (TBA)_2_ **1** and VOCl_3_

Volatiles were removed from methanolic (TBA)­OH (0.93 *M*, 5.9 mL, 5.5 mmol) to give an oil, which was dissolved in MeCN (5 mL) and the solvent removed again. The process was repeated twice more to ensure complete removal of the MeOH. The oil was dissolved in MeCN (15 mL) and (TBA)_2_ **1** (2.0 g, 1.1 mmol) was added. The suspension was stirred at room temperature until the solid had dissolved (∼15 min) before removal of the volatiles and redissolution of the residue in MeCN (5 mL). The mixture was pumped dry, the process repeated and the residue dissolved in pyridine (15 mL). VOCl_3_ (0.12 mL, 0.22 g, 1.3 mmol) was added to give a red/brown solution, which rapidly became green/brown, and the mixture was stirred at room temperature overnight before addition of iodine (1–2 crystals) to oxidize any unwanted V^IV^, which removed the green color and gave a brown solution. Volatiles were removed and the crude yellow/brown solid was dissolved in the minimum amount of hot MeCN and cooled to −30 °C in a freezer overnight. Crystals were isolated by vacuum filtration, washed with cold dichloromethane (3 × 2 mL) and diethyl ether (2 × 20 mL) then dried under vacuum overnight at 50 °C (1.9 g, 75%). The identity of the product was confirmed by ^51^V NMR spectroscopy, which showed a major peak at −508 ppm (CD_3_CN) and minor impurity peaks at −510 and −364 ppm.

#### (TBA)_3_[NbW_5_O_19_] (TBA)_3_ **8**

##### Method A – From (TBA)_2_ **1** and Nb­(OEt)_5_

Volatiles were removed from methanolic (TBA)­OH (1.0 *M*, 1.7 mL, 1.7 mmol) to give an oil, which was dissolved in MeCN (2–3 mL) and the solvent was removed again. This process was repeated twice more to ensure complete removal of the MeOH. The oil was dissolved in MeCN (20 mL) and (TBA)_2_ **1** (2.0 g, 1.1 mmol) was added. The suspension was stirred at room temperature until the solid had dissolved (60–90 min). ^17^O-enriched H_2_O (40% enrichment, 42.0 μL, 2.3 mmol) was added and the solution was stirred overnight. The mixture was transferred via cannula to a separate flask containing Nb­(OEt)_5_ (0.40 g, 1.3 mmol) and the colorless solution was stirred at room temperature overnight. After removal of the volatiles, the remaining solid was dissolved in the minimum amount of hot MeCN and cooled to −30 °C in a freezer overnight. Crystals were isolated by vacuum filtration, washed with diethyl ether (2 × 20 mL) and dried under vacuum overnight at 50 °C. (2.0 g, 77%) The solid can also be recrystallized by slow evaporation of a saturated DCM solution. ^93^Nb NMR (122.4 MHz, *d*_6_-DMSO) δ (ppm): −883. ^17^O NMR (40.7 MHz, CD_3_CN) δ (ppm): Nb=O not resolved, 734 (W=O,), 458 (NbOW), 394 (WOW), −65 (μ_6_-O). ^183^W NMR (20.8 MHz, *d*_6_-DMSO) δ (ppm): 29.4 (1W, W_ax_), 74.5 (4W, W_eq_). *v*_max_/cm^–1^ = 2959, 2873, 1482, 1380, 1151, 1107, 1056, 1027, 953 (vs), 915, 885, 789 (vs), 585, 431 (vs).

##### Method B – From WO_3_·H_2_O and Nb­(OEt)_5_

Volatiles were removed from methanolic (TBA)­OH (1.0 *M*, 6.95 mL, 6.95 mmol) before addition of DMF (ca. 4 mL) and WO_3_·H_2_O (2.88 g, 11.53 mmol) with stirring and heating to 180 °C. After 15 min the resulting solution was transferred onto Nb­(OEt)_5_ (0.73 g, 2.31 mmol) via cannula and washed through with extra DMF (2 mL). After addition of ^17^O-enriched H_2_O (40% enrichment, 50 μL, 2.8 mmol), the stirred mixture was heated for 45 min. The ^183^W NMR spectrum of the reaction solution showed the presence of **1**, **8** and [W_10_O_32_]^4–^. After returning the NMR sample to the main reaction mixture, heated was continued at 180 °C for a further 2.5 h. Crystals that formed upon cooling were separated, washed with diethyl ether, pumped dry and were shown to be (TBA)_3_ **8** by ^17^O NMR (1.35 g, 29%). Addition of diethyl ether to the mother liquor gave a precipitate that was washed with diethyl ether and dried under vacuum (2.32 g). ^17^O and ^183^W NMR spectra of this solid showed the presence of (TBA)_3_ **8** (ca. 50%) in addition to **1** and [W_10_O_32_]^4–^. The overall yield of (TBA)_3_ **8** was *ca*. 2.5 g, 53%.

##### Method C – From (TBA)_2_ **1** and [NbOCl_3_(MeCN)_2_]

Volatiles were removed from methanolic (TBA)­OH (0.93 *M*, 1.5 mL, 1.4 mmol) to give an oil, which was dissolved in MeCN (2–3 mL). The solvent was removed again and the process repeated twice more to ensure complete removal of the MeOH. The oil was dissolved in MeCN (5 mL), ^17^O-enriched (TBA)_2_ **1** (0.5 g, 0.26 mmol) was added and the suspension was stirred at room temperature until the solid had dissolved (∼15 min). The volatiles were removed, and the oil was redissolved in MeCN (5 mL). The mixture was pumped dry and this process was repeated to give a residual oil that was dissolved in pyridine (5 mL). Addition of [NbOCl_3_(MeCN)_2_] (94 mg, 0.32 mmol) gave a colorless solution which was stirred overnight and then passed through a PTFE syringe filter before removal of the volatiles. The oily residue was dissolved in the minimum amount of hot MeCN and cooled to −30 °C in a freezer overnight to afford colorless crystals which were separated from the mother liquor by cannula filtration and dried under vacuum to give a colorless solid (0.43 g, 67%). The product was characterized by ^17^O NMR spectroscopy, and minor peaks in the spectrum at 415 and 777 ppm indicated the presence of a small amount of (TBA)_2_ **1**.

#### (TBA)_3_[TaW_5_O_19_] (TBA)_3_ **9**

##### Method A – From (TBA)_2_ **1** and Ta­(OEt)_5_

Volatiles were removed from methanolic (TBA)­OH (1 *M*, 1.7 mL, 1.7 mmol) to give an oil, which was dissolved in MeCN (2–3 mL), pumped dry and the process repeated twice more to ensure complete removal of the MeOH. The remaining oil was dissolved in MeCN (20 mL) and (TBA)_2_ **1** (2 g, 1.1 mmol) was added with stirring at room temperature until all of the solid had dissolved (60–90 min). ^17^O-enriched H_2_O (40% enrichment, 42.0 μL, 2.3 mmol) was added and solution was stirred overnight. The mixture was transferred via cannula to a separate flask containing Ta­(OEt)_5_ (0.52 g, 1.3 mmol) and the colorless solution was stirred at room temperature overnight. After removal of the volatiles, the remaining solid was dissolved in the minimum amount of hot MeCN and cooled to −30 °C in a freezer overnight. Crystals isolated by vacuum filtration were washed with diethyl ether (2 × 20 mL) and dried under vacuum overnight at 50 °C. (1.9 g, 70%) The solid can also be recrystallized by slow evaporation of a saturated DCM solution. ^17^O NMR (40.7 MHz, CD_3_CN) δ (ppm): Ta=O not resolved, 736 (W=O), 422 (TaOW), 395 (WOW), −73 (μ_6_-O). ^183^W NMR (20.8 MHz, *d*_6_-DMSO) δ (ppm): 39.7 (1W, W_ax_), 69.7 (4W, W_eq_). *v*_max_/cm^–1^: 2959, 2873, 1481, 1381, 1152, 1107, 1054, 1026, 953 (vs), 906, 884, 783 (vs), 574, 496, 426 (vs).

##### Method B – From WO_3_·H_2_O and Ta­(OEt)_5_

DMF (4 mL) and WO_3_·H_2_O (2.15 g, 8.61 mmol) were added to the residue obtained by removal of the volatiles from methanolic (TBA)­OH (1 *M*, 2.9 mL, 2.9 mmol) and the mixture was stirred and heated to 180 °C for 10 min to give a slightly hazy solution. The pressure was reduced briefly to remove volatile components and the solution was allowed to cool to room temperature before addition of methanolic (TBA)­OH (1 *M*, 2.3 mL, 2.3 mmol), 40% ^17^O-enriched H_2_O (200 μL, 11.1 mmol) and MeCN (10 mL) with stirring. After 20 min, the solution was transferred onto Ta­(OEt)_5_ (0.78 g, 1.92 mmol) and the reaction mixture was stirred for 16 h at room temperature. Addition of diethyl ether gave a colorless precipitate, which was filtered in air, washed with diethyl ether (10 mL) and pumped dry to give a product which was shown by ^17^O NMR to be >90% pure (3.13 g). The product was dissolved in MeCN and centrifuged to remove a small amount of suspended fine solid before concentration, saturation by addition of diethyl ether and cooling in the freezer to give colorless crystals (2.46 g) which contained ∼4% (TBA)_2_ **1** impurity.

### Alkoxide Protolytic Metathesis Reactions

#### (TBA)_3_[(PhCO_2_)­ZrW_5_O_18_] (TBA)_3_ **12**

Benzoic acid (0.04 g, 0.33 mmol) was added to a solution of (TBA)_6_ **5** (0.40 g, 0.097 mmol) in MeCN (7 mL) with stirring. After 1 h, the volume was reduced under reduced pressure to about 1 mL and diethyl ether (25 mL) was added to precipitate the product. The supernatant liquid was removed by cannulation and the solid was washed with diethyl ether (2 × 5 mL) then dried under vacuum to give a quantitative yield (0.41 g). Single crystals for X-ray diffraction were obtained by cooling a saturated solution in MeCN to −30 °C. ^1^H NMR (300.13 MHz, CD_3_CN) δ (ppm): 7.50 (2H, t), 7.64 (1H, t), 8.10 (2H, d). ^13^C NMR (75.48 MHz, *d*_6_-DMSO) δ (ppm): 128.5, 129.4, 132.5, 133.5 (*C*_6_H_5_) and 175.1 (br, *C*O_2_). ^17^O NMR (40.7 MHz, CD_3_CN) δ (ppm): 723, 705 (W=O), 478 (ZrOW), 392, 382 (WOW), −50 (μ_6_-O). ^183^W NMR (20.8 MHz, DMSO) δ (ppm): 42.0 (4W, W_eq_), 77.9 (1W, W_ax_).

#### (TBA)_3_[(PhCO_2_)­HfW_5_O_18_] (TBA)_3_ **13**

(TBA)_6_ **6** (0.81 g, 0.19 mmol) and benzoic acid (0.04 g, 0.38 mmol) were dissolved in MeCN (5 mL) and stirred overnight. Volatiles were removed under reduced pressure and the resulting solid was triturated with diethyl ether before drying under vacuum and recrystallization from the minimum amount of MeCN by cooling in the freezer. The crystals were separated and dried (0.43 g) and a further crop was obtained from the mother liquor after reduction in the volume and cooling in the freezer (0.10 g). Total yield 0.53 g, 63%. ^1^H NMR (300.13 MHz, CD_3_CN) δ (ppm): 7.53 (2H, t), 7.68 (1H, t), 8.12 (2H, d). ^13^C NMR (176.07 MHz, *d*_6_-DMSO) δ (ppm): 128.5, 129.4, 132.6, 133.9 (*C*_6_H_5_), 177.5 (br, *C*O_2_). ^17^O NMR (40.7 MHz, CD_3_CN) δ (ppm): 721, 700 (W=O), 455 (HfOW), 391, 381 (WOW), −53 (μ_6_-O). ^183^W NMR (20.8 MHz, DMSO) δ (ppm): 45.4 (4W, W_eq_), 65.5 (1W, W_ax_).

#### (TBA)_3_[(acac)­HfW_5_O_18_] (TBA)_3_ **14**

Acetylacetone (39 μL, 0.38 mmol) was added to (TBA)_6_ **6** (0.83 g, 0.19 mmol) in MeCN (5 mL) and the clear, colorless solution was stirred overnight to give a yellow solution. After removal of the volatiles under reduced pressure, the residue was triturated with diethyl ether to give a quantitative yield of a pale yellow solid (0.87 g). Single crystals were obtained by recrystallization from MeCN/diethyl ether. ^1^H NMR (300.13 MHz, CD_3_CN) δ (ppm): 2.03 (6H, s, C*H*_3_), 5.72 (1H, s, C*H*). ^13^C NMR (75.48 MHz, CD_3_CN) δ (ppm): 26.0 (*C*H_3_), 103.5 (*C*H), 190.1 (*C*O). ^17^O NMR (40.7 MHz, CD_3_CN) δ (ppm): 714, 695 (W=O), 462 (HfOW), 388, 377 (WOW), −60 (μ_6_-O). ^183^W NMR (20.8 MHz, CD_3_CN) δ (ppm): 52.1 (4W, W_eq_), 62.0 (1W, W_ax_).

## Results and Discussion

### Synthesis of (TBA)_2_ **1** from WO_3_

To facilitate our investigations into base-promoted conversion of (TBA)_2_ **1** to {MW_5_} Lindqvist-type POMs, we simplified the synthesis of (TBA)_2_ **1** starting material by treating WO_3_·H_2_O with a stoichiometric amount of (TBA)­OH in DMF at 180 °C according to eq 3, whereupon the yellow suspension of the oxide dissolved to give a colorless solution over about 20 min. The ^183^W NMR spectrum of the reaction solution recorded at 100 °C contained a major peak at 64 ppm due to **1** and a minor impurity peak at −18 ppm that was assigned to the equatorial tungsten atoms of [W_10_O_32_]^4–^ (Figure S1a).

3 $${\mathrm{6 WO}}_{3}{\cdot H}_{2}O+2\left(\mathrm{TBA}\right)\mathrm{OH}\to {\left(\mathrm{TBA}\right)}_{2}\left[W_{6}O_{19}\right]{+\mathrm{7 H}}_{2}O$$

The solvent volume was kept to a minimum and the product was obtained in almost quantitative yield by addition of diethyl ether to the cooled solution. Recrystallization from hot MeCN afforded pure (TBA)_2_ **1** as shown by ^183^W NMR (Figure S1b). This synthesis is more convenient than the previously reported acidification of Na_2_WO_4_·2H_2_O,^3^ providing higher yields from fewer reagents, and is easily scalable.

### Synthesis of {M^IV^W_5_} Derivatives from (TBA)_2_ **1**

Interest in reactive {M^IV^W_5_} Lindqvist POMs^6d,11^ prompted us to investigate whether the alkoxido-substituted (TBA)_3_[(RO)­M^IV^W_5_O_18_] (M = Ti, Sn) and dimeric (TBA)_6_[{(RO)­MW_5_O_18_}_2_] (M = Zr, Hf) might be accessible from (TBA)_2_ **1** according to eq 4.

4 $$5{\left(\mathrm{TBA}\right)}_{2}\left[W_{6}O_{19}\right]+8\left(\mathrm{TBA}\right)\mathrm{OH}+6M{\left(\mathrm{OR}\right)}_{4}+5H_{2}O\to 6{\left(\mathrm{TBA}\right)}_{3}\left[\left(\mathrm{RO}\right){\mathrm{MW}}_{5}O_{18}\right]+18\mathrm{ROH}$$

These reactions were carried out stepwise, using a solution of (TBA)­OH prepared from a stock methanolic solution by evaporation and redissolution in MeCN. (TBA)_2_ **1** gradually dissolved when stirred with 1.6 mol-equivalents of (TBA)­OH at ambient temperature prior to addition of 1.0 mol-equivalent of H_2_O to the reaction mixture and subsequent transfer of this reaction mixture onto the heterometal alkoxide in MeCN. TBA salts of [(^i^PrO)­TiW_5_O_18_]^3–^ **2**, [(MeO)­MW_5_O_18_]^3–^ (M = Ti **3**, Sn **4**) and [{(MeO)­MW_5_O_18_}_2_]^6–^ (M = Zr **5**, Hf **6**) were obtained in good yields after recrystallization from MeCN. Syntheses were routinely monitored by ^17^O NMR spectroscopy by starting with ^17^O-enriched (TBA)_2_ **1** or by using ^17^O-enriched H_2_O in the reactions, whereupon the initially complex ^17^O NMR spectra produced upon base degradation of **1** simplified dramatically upon reaction with M­(OR)_4_ to give spectra characteristic of [(RO)­MW_5_O_18_]^3–^ species. FTIR and NMR (^1^H, ^17^O, ^183^W and ^119^Sn) spectra of recrystallized products were consistent with those previously prepared using our hydrolytic assembly approach. Specific details are provided below.

(TBA)_3_[(^i^PrO)­TiW_5_O_18_], (TBA)_3_ **2** and (TBA)_3_[(MeO)­TiW_5_O_18_], (TBA)_3_ **3** – The ^17^O NMR spectrum of (TBA)_2_ **1** shown in Figure 1a contains peaks at 777 ppm for W=O, 416 for WOW and −79 for μ_6_-O. (The latter is generally not visible unless long pulse delays are used because of the long *T*_1_ associated with the high symmetry of this site).^10^ After treatment of (TBA)_2_ **1** with (TBA)­OH and ^17^O-enriched H_2_O the spectrum shown in Figure 1b indicates complex solution speciation, which will be addressed in a separate paper, but treatment with Ti­(O^i^Pr)_4_ gave the much simpler spectrum in Figure 1c showing peaks for **2** and a small amount of hydrolysis product [(μ-O)­(TiW_5_O_18_)_2_]^6–^, as evidenced by the μ_2_-O peak at 632 ppm.^6d^ This remarkably clean reaction enabled the large scale (∼20 g) synthesis of ^17^O-enriched (TBA)_3_ **2**. The presence of hydrolysis products can be mitigated by converting all {TiW_5_} species to the methoxido derivative (TBA)_3_ **3** in high yield by adding an excess of MeOH to the reaction mixture as shown in Figure 1d. The spectrum of recrystallized (TBA)_3_ **3** in Figure 1e is typical of [(RO)­TiW_5_O_18_]^3–^ species, which have similar ^17^O NMR spectra but can be distinguished by their ^1^H NMR spectra (Figure S2). A key feature of this synthetic approach is the absence of (TBA)_2_ **1** in the products, which enables higher yields upon simple recrystallization and straightforward scale-up, facilitating highly concentrated solutions for ^183^W NMR spectra with sufficient resolution to observe ^2^ *J*(^183^W_eq_^183^W_ax_) coupling of 7 Hz for the first time (Figure S3).

**Figure 1:**
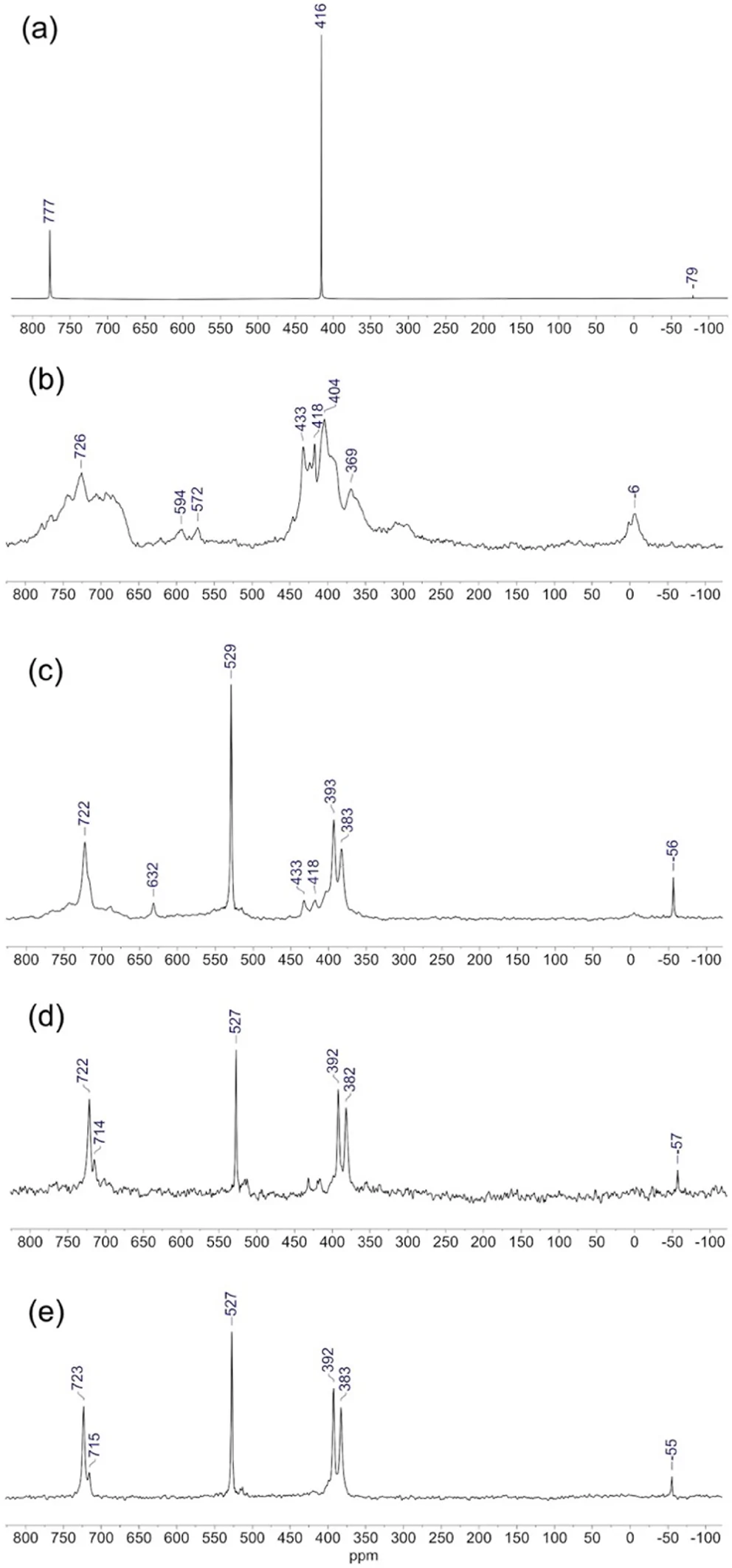
^17^O NMR spectra of: (TBA)_2_ **1** before (a) and after (b) treatment with 1.6 mol-equivalents of (TBA)­OH and 1.0 mol-equivalent of 40% ^17^O-enriched H_2_O; reaction mixture 30 min after addition of Ti­(O^i^Pr)_4_ (c); reaction mixture after addition of an excess of MeOH (d) and recrystallized (TBA)_3_ **3** (e).

(TBA)_3_[(MeO)­SnW_5_O_18_] (TBA)_3_ **4** – The treatment of Sn­(O^t^Bu)_4_ with base-degraded (TBA)_2_ **1** followed by addition of an excess of MeOH gave improved yields and purity of moisture-sensitive (TBA)_3_ **4** than previously obtained with our hydrolytic method.^4b^ For example, a 20 g scale reaction gave a 71% yield of ^17^O-enriched (TBA)_3_ **4** compared to 36% with the hydrolytic method. ^17^O and ^119^Sn NMR spectra after the various stages of reaction are shown in Figure S4. The major peak at −649 ppm in the ^119^Sn NMR spectrum of the initial product can be assigned to (TBA)_3_ **4**,^4b^ suggesting that not all of the MeOH had been removed from the methanolic (TBA)­OH. The peak at −665 ppm was assigned to [(^t^BuO)­SnW_5_O_18_]^3–^ by rerecording the spectrum after treatment with an excess of ^t^BuOH to differentiate from [(μ-O)­(SnW_5_O_18_)_2_]^6–^, which has a similar chemical shift.^6c,6d^ The small peak at −614 ppm may be due to [ClSnW_5_O_18_]^3–^ resulting from a chlorido impurity in the Sn­(O^t^Bu)_4_. However, after treatment with an excess of MeOH, the ^119^Sn NMR spectrum contained only the peak for (TBA)_3_ **4** at −649 ppm.

(TBA)_6_[{(MeO)­ZrW_5_O_18_}_2_] (TBA)_6_ **5** – Following our brief report of the reaction between base-degraded (TBA)_2_ **1** and [{Zr­(O^i^Pr)_4_(^i^PrOH)}_2_],^6e^ we optimized this procedure to provide more reliable access to pure (TBA)_6_ **5**. ^17^O NMR monitoring (Figure S5) showed that (TBA)_6_ **5** was obtained after treatment of the initial product with an excess of MeOH, and an improved X-ray crystal structure was obtained from single crystals grown from MeCN.

(TBA)_6_[{(MeO)­HfW_5_O_18_}_2_] (TBA)_6_ **6** – Following our preliminary report,^4c^ we now provide full details for the dimeric hafnium-substituted (TBA)_6_ **6**, which we have prepared in good yield from (TBA)_2_ **1** and [{Hf­(O^i^Pr)_4_(^i^PrOH)}_2_] after recrystallization from MeOH. The ^1^H NMR spectrum contains a peak for μ-OMe at 3.83 ppm (Figure 2) and the corresponding peak in the ^13^C NMR spectrum appears at 58.0 ppm. In the ^17^O NMR spectrum shown in Figure 3, peaks are observed for W=O at 712 and 691, HfOW at 464, overlapping broad WOW at 387 and the central μ^6^-O at −61 ppm. The W_ax_ and W_eq_ peaks for **6** appear at 68.7 and 54.7 ppm respectively in the ^183^W NMR spectrum (Figure 4), which also contains minor impurity peaks.

**Figure 2:**
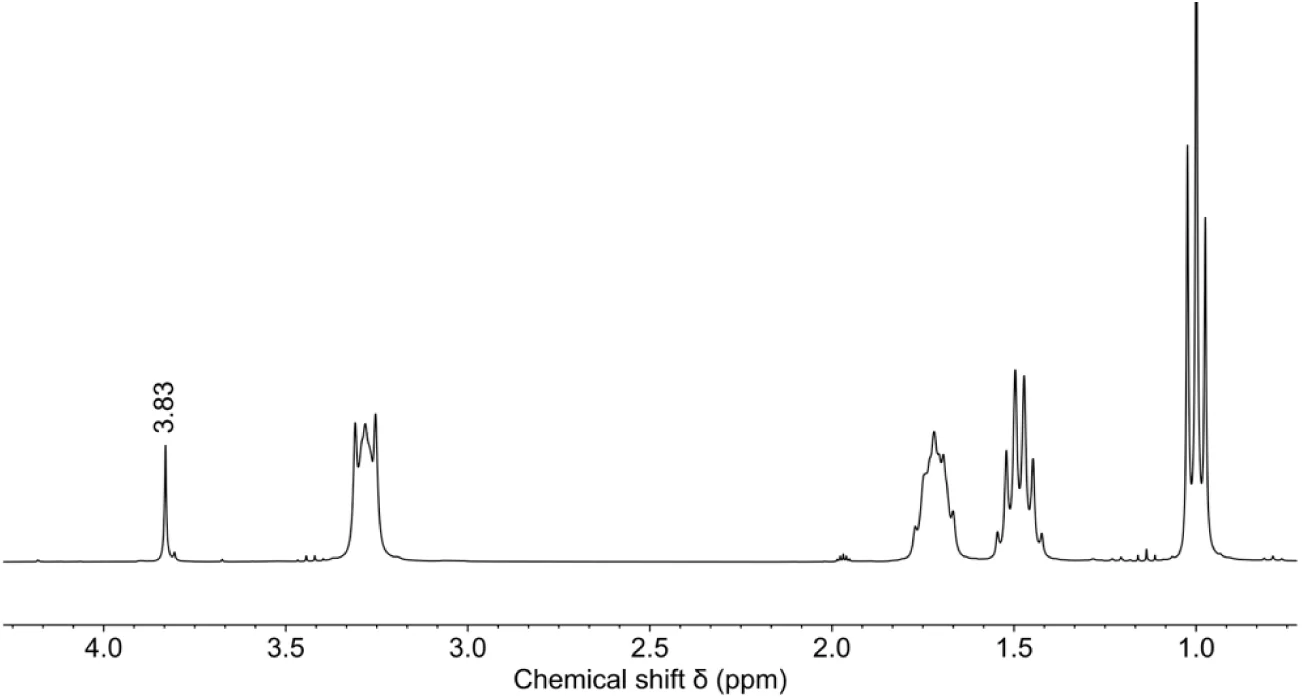
^1^H NMR spectrum of (TBA)_6_[{(MeO)­HfW_5_O_18_}_2_] (TBA)_6_ **6** recorded in CD_3_CN.

**Figure 3:**
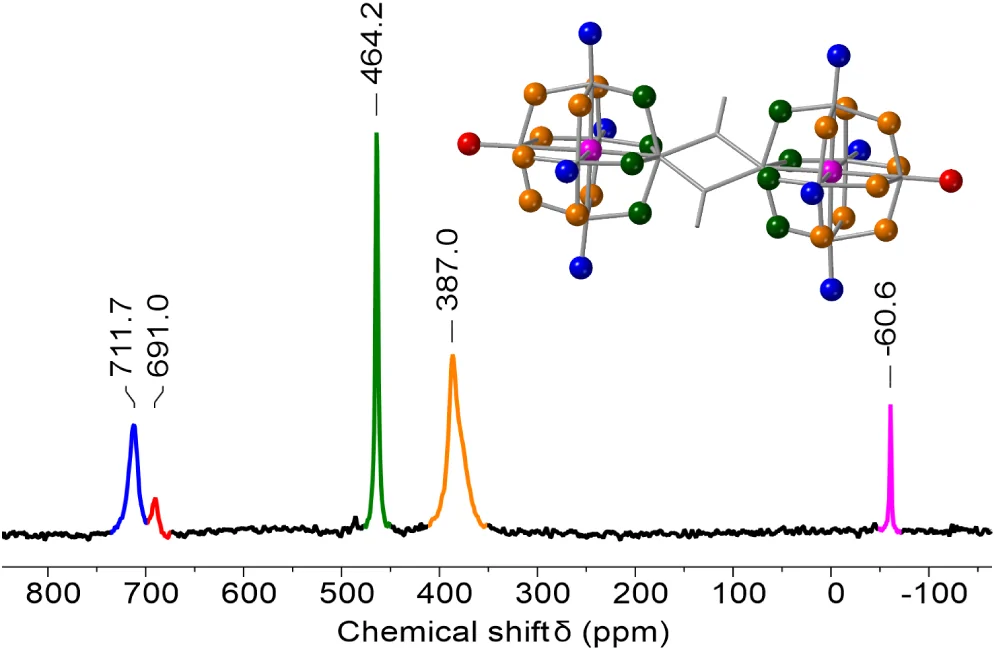
^17^O NMR spectrum of (TBA)_6_[{(MeO)­HfW_5_O_18_}_2_] (TBA)_6_ **6** recorded in CD_3_CN.

**Figure 4:**
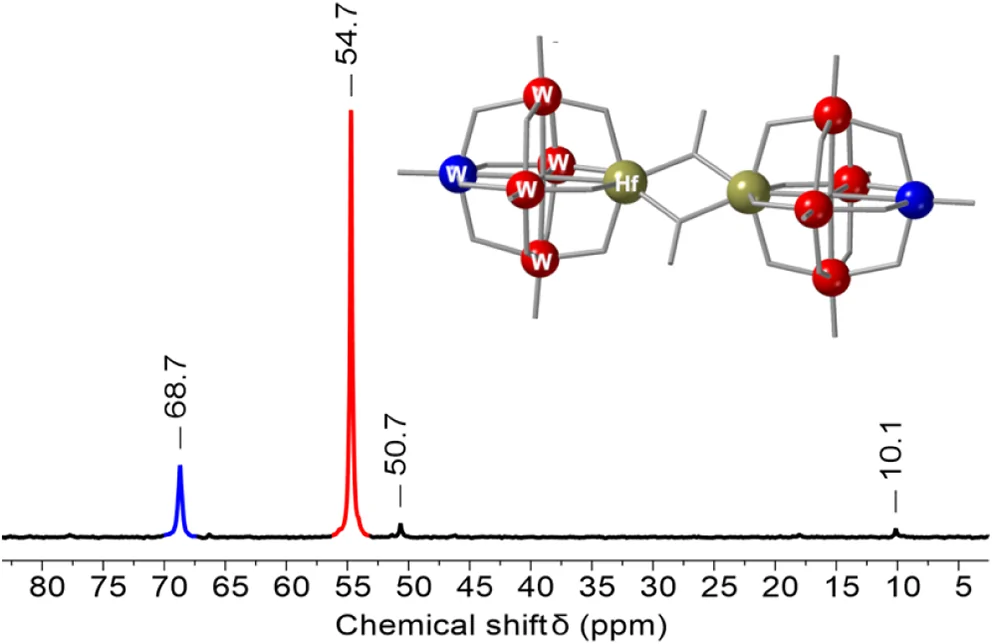
^183^W NMR spectrum of (TBA)_6_[{(MeO)­HfW_5_O_18_}_2_] (TBA)_6_ **6** recorded in CD_3_CN.

Crystallographic parameters for isostructural (TBA)_6_ **5** and (TBA)_6_ **6** are given in Table S1 and bond lengths and angles for anions **5** and **6** (Figure 5) are compared in Table S2. The structures of **5** and **6** are equivalent, and apparent differences between Zr–OMe, Zr–OW_eq_ and Zr−μ_6_O of 2.148, 2.115, and 2.366 Å respectively in **5** and Hf–OMe, Hf–OW_eq_ and Hf−μ_6_O of 2.131, 2.097, and 2.352 Å in **6** are within the error range. The MOW_eq_ bridges to the lacunary {W_5_O_18_} fragment have longer M–O and shorter W_eq_–O bonds, and the equatorial plane of the {W_5_O_18_} unit has alternating long and short W_eq_–O bonds. The W_eq_OW_ax_ linkages are similarly asymmetric, with longer W_eq_–O and shorter W_ax_–O bonds.

**Figure 5:**
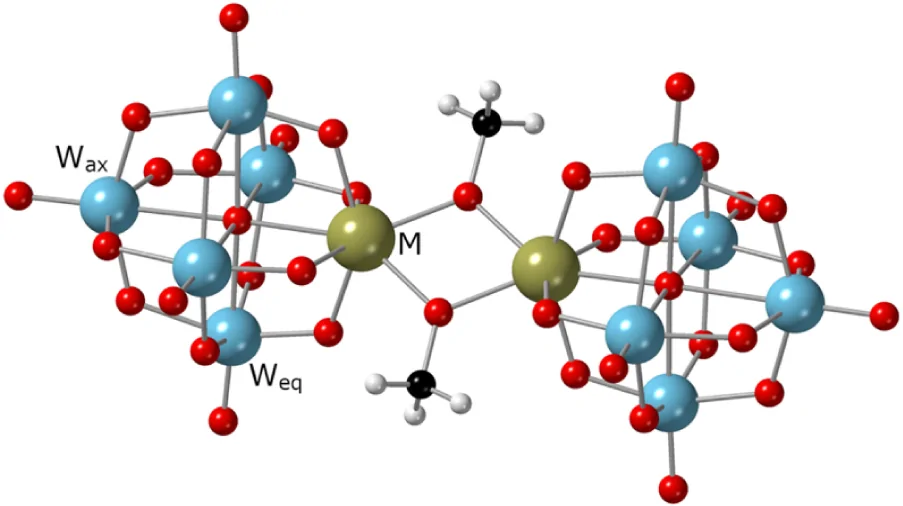
Structure of [{(MeO)­MW_5_O_18_}_2_]^6–^ anions in (TBA)_6_ **5** (M = Zr) and (TBA)_6_ **6** (M = Hf).

### Synthesis of {M^V^W_5_} Derivatives from (TBA)_2_ **1**

An analogous degradative protocol was used to prepare Group 5 heterometal-substituted Lindqvist anions [MW_5_O_19_]^3–^ (M = V, **7**; Nb, **8**; Ta, **9**) by reacting base-degraded (TBA)_2_ **1** with alkoxides VO­(O^t^Bu)_3_ or M­(OR)_5_ (M = Nb, Ta) according to eqs 4 and 6.

5 $$5{\left(\mathrm{TBA}\right)}_{2}\left[W_{6}O_{19}\right]+8\left(\mathrm{TBA}\right)\mathrm{OH}+\mathrm{6 VO}{\left(\mathrm{OR}\right)}_{3}{+\mathrm{5 H}}_{2}O\to 6{\left(\mathrm{TBA}\right)}_{3}\left[{\mathrm{VW}}_{5}O_{19}\right]+\mathrm{18 ROH}$$

6 $$5{\left(\mathrm{TBA}\right)}_{2}\left[W_{6}O_{19}\right]+8\left(\mathrm{TBA}\right)\mathrm{OH}+\mathrm{6 M}{\left(\mathrm{OR}\right)}_{5}{+\mathrm{11 H}}_{2}O\to 6{\left(\mathrm{TBA}\right)}_{3}\left[{\mathrm{MW}}_{5}O_{19}\right]+\mathrm{30 ROH}$$

(TBA)_3_[VW_5_O_19_] (TBA)_3_ **7** – The vanadium-substituted hexatungstates first reported by Flynn and Pope included a range of [V_2_W_4_O_19_]^4–^ and [VW_5_O_19_]^3–^ salts obtained by acidification of aqueous mixtures of [WO_4_]^2–^ and [VO_3_]^−^, and quaternary ammonium salts were obtained from alkali metal salts by cation exchange.^12^ Klemperer reported a nonaqueous synthesis of (TBA)_3_ **7** involving initial digestion of V_2_O_5_ with (TBA)­OH in acetonitrile to generate a soluble vanadate precursor mixture followed by treatment with (TBA)_4_[W_10_O_32_] under reflux for 6 h.^13^ Our previous attempts to synthesize (TBA)_3_ **7** by hydrolysis of VO­(OMe)_3_ and WO­(OMe)_4_ in the presence of (TBA)_2_[WO_4_] gave impure products containing **1** and other vanadate species,^4a^ whereas the crude product from a small-scale degradative reaction with ^17^O-enriched (TBA)_2_ **1** after stirring overnight at room temperature and removal of the solvent gave the ^17^O NMR spectrum shown in Figure 6, which shows clean formation of (TBA)_3_ **7** and only minor impurity peaks. The peaks at 1219.8 and 565.8 ppm can be assigned to the terminal V=O and V–O–W bridges respectively and are significantly broadened compared to others in the spectrum due to coupling to the quadrupolar ^51^V nucleus (I = 7/2). The remaining peaks are sharper and assigned to W=O at 735.1 ppm, WOW at 398.6 and 391.9 ppm and central μ_6_-O at −72.4 ppm.

**Figure 6:**
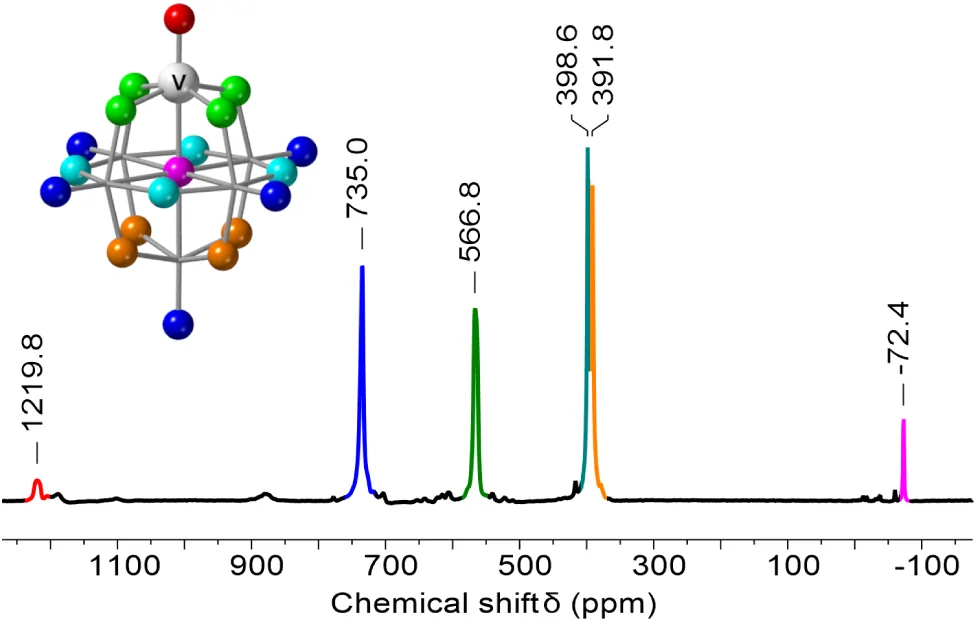
^17^O NMR spectrum of crude (TBA)_3_ **7** produced by treatment of ^17^O-enriched (TBA)_2_ **1** with 1.6 eq. of TBA­(OH), followed by reaction with VO­(O^t^Bu)_3_, recorded in CD_3_CN.

The crude product from a larger scale reaction was readily recrystallized from acetonitrile and ^51^V and ^183^W NMR spectra of the resulting yellow crystals are shown in Figure 7. The ^51^V NMR spectrum shows a characteristic single peak at −508 ppm for (TBA)_3_ **7**,^14^ while the ^183^W NMR spectrum contains a broad feature that contains two overlapping peaks at 78.4 and 79.6 ppm that were previously assigned to the two tungsten environments of (TBA)_3_ **7** as indicated in Figure 7.^14,15^ The 1.2 ppm difference between the axial (blue) and equatorial (red) chemical shifts is much smaller than that observed for other {MW_5_} Lindqvist anions.^4,6^

7 $$5{\left(\mathrm{TBA}\right)}_{2}\left[W_{6}O_{19}\right]+8\left(\mathrm{TBA}\right){\mathrm{OH}+\mathrm{3 V}}_{2}O_{5}\to 6{\left(\mathrm{TBA}\right)}_{3}\left[{\mathrm{VW}}_{5}O_{19}\right]{+\mathrm{4 H}}_{2}O$$

**Figure 7:**
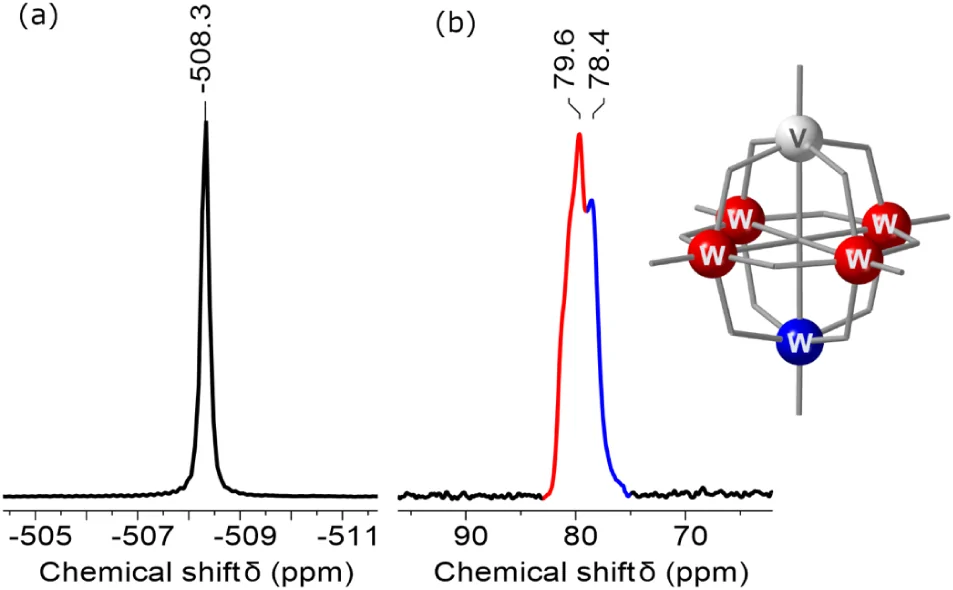
^51^V (a) and ^183^W (b) NMR spectra of (TBA)_3_ **7**. The ^51^V NMR spectrum was recorded in CD_3_CN and the ^183^W NMR spectrum was recorded in *d*_6_-DMSO.

The synthesis of (TBA)_3_ **7** was further simplified by using V_2_O_5_ instead of VO­(O^t^Bu)_3_ according to eq 6, and ^17^O-enrichment was achieved by addition of ^17^O-enriched H_2_O to the degraded (TBA)_2_ **1**. Elevated temperatures were required to fully dissolve the V_2_O_5_ so, to avoid the formation of acetamide resulting from the reaction between MeCN and (TBA)­OH under these conditions, a minimum amount of DMF was used as the solvent. The ^51^V NMR spectrum of the hot reaction mixture contained a single peak with a temperature-dependent chemical shift, as shown in Figure S6a, indicating the quantitative formation of **7**.^16^ Pure (TBA)_3_ **7** was isolated by precipitation in 88% yield as a pure compound, as confirmed by the ^17^O NMR spectrum shown in Figure S6b.

(TBA)_3_[NbW_5_O_19_] (TBA)_3_ **8** – The niobium-substituted [NbW_5_O_19_]^3–^ **8** was first obtained by the acidification of an aqueous solution of Na_2_WO_4_ and the hexaniobate K_8_[Nb_6_O_19_].^17–19^ We found that treatment of Nb­(OEt)_5_ with an acetonitrile solution generated from (TBA)_2_ **1**, TBA­(OH) and H_2_O according to eq 5 gave (TBA)_3_ **8** in 77% yield after crystallization. The ^17^O NMR spectrum of the resulting colorless crystals is shown in Figure 8. Peaks at 734.2 and 393.9 ppm are assigned to terminal W=O and W–O–W respectively. Although separate peaks are expected for the two W=O and two W–O–W environments, these are not resolved because of broadening due to coupling between ^17^O and quadrupolar Nb (*I* = 9/2). This broadening is even more pronounced for the Nb–O–W peak at 456.9 ppm, and for terminal Nb=O, which is difficult to observe without ^93^Nb decoupling, although Klemperer reported a chemical shift of 799 ppm in the ^17^O­{^93^Nb} decoupled spectrum.^20^ A relatively sharp peak for the central μ_6_-O is observed at −64.8 ppm. Low intensity peaks in the ^17^O NMR spectrum of (TBA)_3_ **8** can be assigned to disubstituted [Nb_2_W_4_O_19_]^4–^,^18^ (Figure S7) which was removed upon recrystallization. The presence of these peaks suggests that (TBA)_4_[Nb_2_W_4_O_19_] might be obtained by adjusting the relative amounts of TBA­(OH) and Nb­(OEt)_5_ used in the reaction, although this is not addressed here. Interestingly, we observed no evidence for the formation of disubstituted (TBA)_4_[V_2_W_4_O_19_] during the synthesis of (TBA)_3_ **7**. ^93^Nb and ^183^W NMR spectra for (TBA)_3_ **8** are shown in Figure 9. The ^93^Nb NMR spectrum contains a single broad peak (fwhm = 2700 Hz) at −883 ppm when recorded in *d*_6_-DMSO. This is consistent with the reported value of −888 ppm obtained in acetonitrile (fwhm = 600–800 Hz).^20^ The different line widths may be attributed to the different dielectric constants and viscosities of the solvents, as well as the order of magnitude greater concentration in the *d*_6_-DMSO solution. The ^183^W NMR spectrum shows signals at 29.4 and 74.5 ppm (in a 1:4 ratio respectively) due to **8** and a smaller peak at 59.2 ppm due to **1** (about 6.5% after a single crystallization), although peaks due to **1** were not observed in the ^17^O NMR spectrum shown in Figure 8. Importantly, the yield of (TBA)_3_ **8** was significantly higher than that reported by Klemperer.^20^ Attempts to use Nb_2_O_5_ instead of Nb­(OEt)_5_ by analogy with eq 6 were unsuccessful.

**Figure 8:**
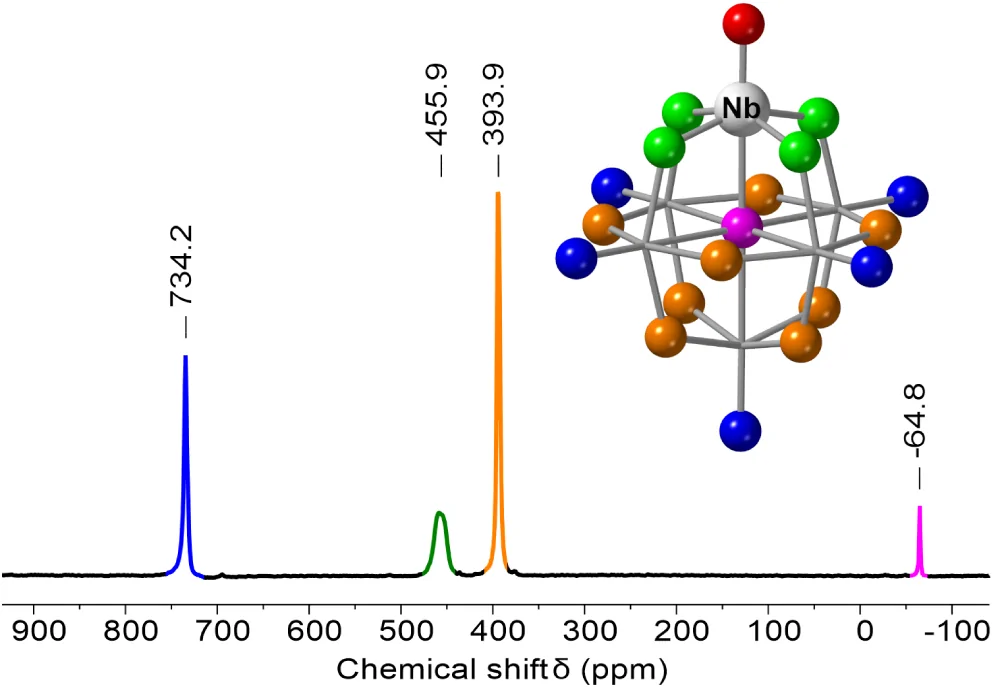
^17^O NMR spectrum of (TBA)_3_ **8** obtained by treatment of ^17^O-enriched (TBA)_2_ **1** with 1.6 eq. of TBA­(OH), followed by reaction with Nb­(OEt)_5_.

**Figure 9:**
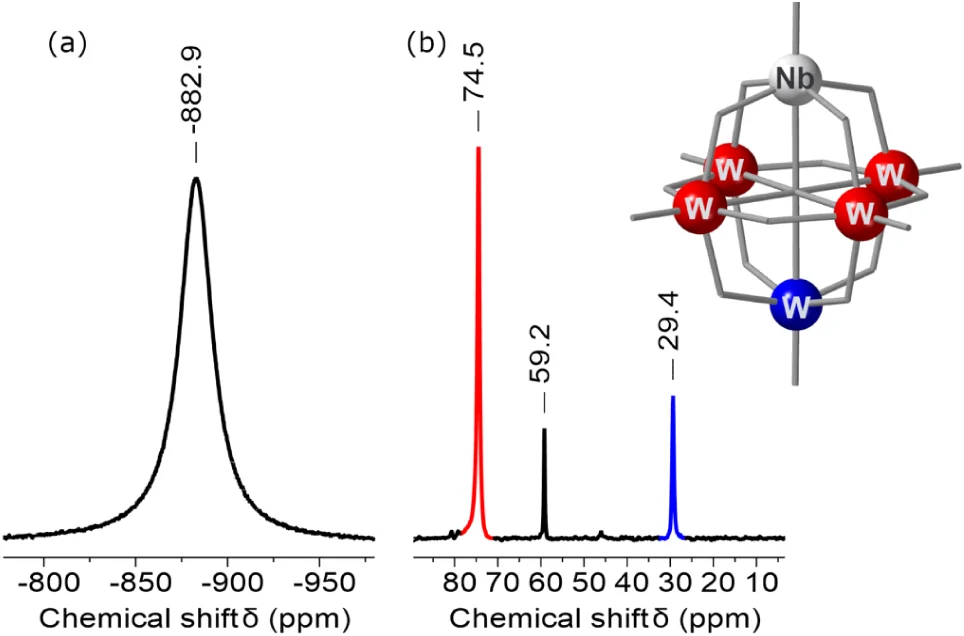
^93^Nb (a) and ^183^W (b) NMR spectra of (TBA)_3_ **8** in *d*_6_-DMSO. The peak at 59.2 ppm in (b) is due to **1**.

(TBA)_3_[TaW_5_O_19_] (TBA)_3_ **9** – Addition of Ta­(OEt)_5_ to the solution obtained by degradation of (TBA)_2_ **1** with 1.6 mol-equivalents of TBA­(OH) gave large colorless crystals of (TBA)_3_ **9** after stirring, removal of the volaties and recrystallization from hot MeCN. The^17^O NMR spectrum of these crystals (Figure 10) was similar to those of (TBA)_3_ **7** and (TBA)_3_ **8** and indicated clean formation of (TBA)_3_ **9**. The peak at 736.3 ppm is assigned to W=O units and, as for **7** and **8**, the expected two peaks in a 4:1 ratio are not resolved. The peaks at 421.6 and 395.0 ppm in a 1:2 ratio are assigned to Ta–O–W and the two nonresolved W–O–W sites respectively, while the peak at −72.7 ppm is due to the central μ^6^-O. The Ta–O–W peak (green) is notably less broadened than VOW in the spectrum of **7** or NbOW in the spectrum of **8**. This can be ascribed to the quadrupole moment of ^181^Ta (*I* = 7/2, Q = 3 b), which is larger than that of ^51^V (*I* = 7/2, Q = −0.05 b) and ^93^Nb (*I* = 9/2, Q = −0.2 b), and serves to spin-decouple ^181^Ta from ^17^O via rapid metal quadrupole relaxation. Despite this, the expected peak for Ta=O is not present at the previously reported chemical shift of 666 ppm, presumably due to a lack of ^17^O enrichment at this site, which is consistent with nonstatistical enrichment observed by Klemperer.^20^

**Figure 10:**
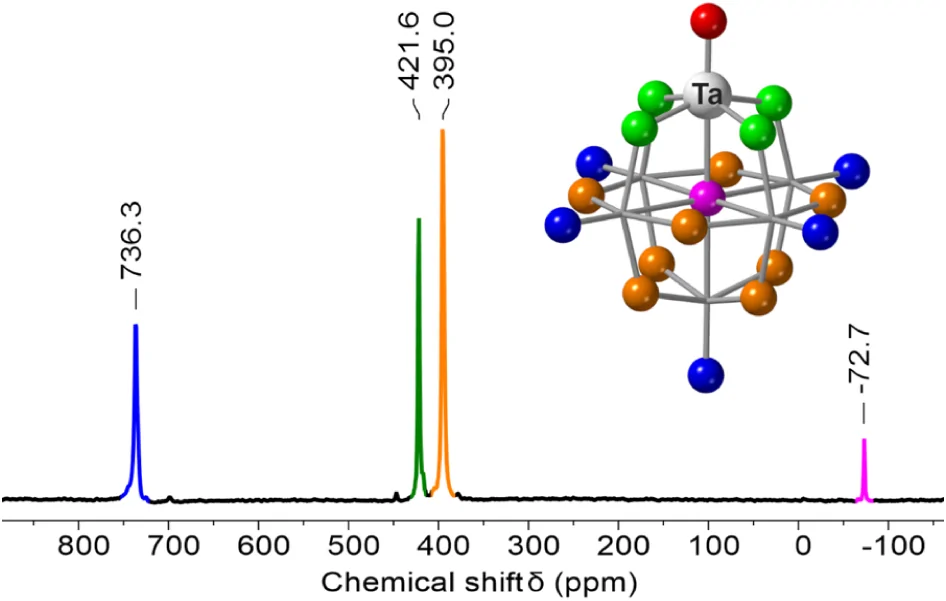
^17^O NMR spectrum of (TBA)_3_ **9** obtained by treatment of ^17^O-enriched (TBA)_2_ **1** with 1.6 eq. of TBA­(OH), followed by reaction with Ta­(OEt)_5_. Spectrum recorded in CD_3_CN.

The ^183^W NMR spectrum of (TBA)_3_ **9** shown in Figure 11 is similar to that of (TBA)_3_ **8**, showing 4:1 ratio peaks at 70.1 and 39.6 ppm respectively, and the small peak at 59.2 ppm can be assigned to (TBA)_2_ **1**. As with the niobium analogue, peaks for **1** were not observed in the ^17^O spectrum but the relevant peak is present in the ^183^W NMR spectrum, perhaps because of the much greater concentration used for ^183^W NMR.

**Figure 11:**
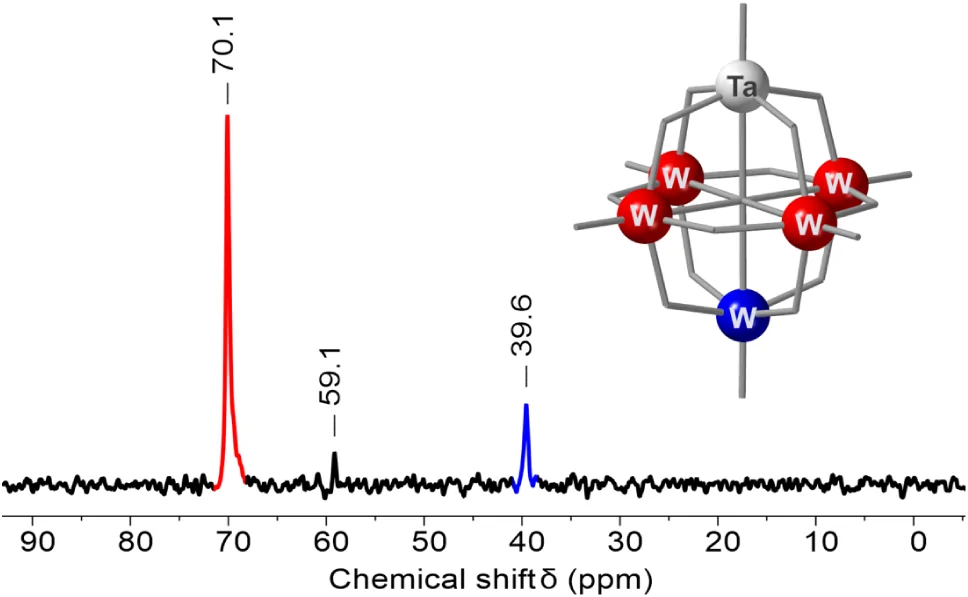
^183^W NMR spectrum of (TBA)_3_ **9** in *d*_6_-DMSO. The peak at 59.1 ppm is due to **1**.

### Modified Procedures from WO_3_·H_2_O

The clean conversion of WO_3_·H_2_O to (TBA)_2_ **1** led us to explore the use of WO_3_·H_2_O as the tungsten precursor in the synthesis of substituted [(RO)­M^IV^W_5_O_18_]^3–^ and [M^V^W_5_O_19_]^3–^ anions according to eqs 8–10.

8 $${5\mathrm{WO}}_{3}{\cdot H}_{2}O+3\left(\mathrm{TBA}\right)\mathrm{OH}+M{\left(\mathrm{OR}\right)}_{4}\to {\left(\mathrm{TBA}\right)}_{3}\left[\left(\mathrm{RO}\right){\mathrm{MW}}_{5}O_{18}\right]{+3\mathrm{ROH}+5H}_{2}O$$

9 $${10\mathrm{WO}}_{3}{\cdot H}_{2}O+6\left(\mathrm{TBA}\right){\mathrm{OH}+V}_{2}O_{5}\to 2{\left(\mathrm{TBA}\right)}_{3}\left[{\mathrm{VW}}_{5}O_{19}\right]{+13H}_{2}O$$

10 $${5\mathrm{WO}}_{3}{\cdot H}_{2}O+3\left(\mathrm{TBA}\right)\mathrm{OH}+M{\left(\mathrm{OEt}\right)}_{5}\to {\left(\mathrm{TBA}\right)}_{3}\left[{\mathrm{MW}}_{5}O_{19}\right]{+5\mathrm{EtOH}+4H}_{2}O$$

The presence of water in the reaction mixture presents a problem with regard to hydrolysis of alkoxido [(RO)­M^IV^W_5_O_18_]^3–^ anions in eq 7, so this approach was not pursued for the M­(IV) derivatives. However, product hydrolysis is not an issue for the [M^V^W_5_O_19_]^3–^ species and, as in the clean synthesis of (TBA)_3_ **7** from (TBA)_2_ **1** and V_2_O_5_ (eq 6), we were able to show that the high temperature reaction between V_2_O_5_, WO_3_·H_2_O and (TBA)­OH in DMF (eq 8) gives a high yield of (TBA)_3_ **7**, although attempts to prepare (TBA)_3_ **8** similarly from WO_3_·H_2_O and Nb_2_O_5_ were unsuccessful due to the poor solubility of Nb_2_O_5_ under these conditions. Soluble alkoxides M­(OEt)_5_ (M = Nb, Ta) were therefore used in attempts to synthesize (TBA)_3_ **8** and (TBA)_3_ **9** according to eq 10. ^183^W NMR spectra of the product from high temperature digestion of WO_3_·H_2_O with (TBA)­OH and treatment with Nb­(OEt)_5_ showed the presence of **1** and [W_10_O_32_]^4–^ impurities. As a result, the method was modified for the synthesis of (TBA)_3_ **9** by first generating **1** *in situ* at high temperature before carrying out the base degradation and the reaction with Ta­(OEt)_5_ at room temperature.

(TBA)_3_[VW_5_O_19_] (TBA)_3_ **7** – The ^183^W NMR spectrum of the reaction mixture obtained by heating a mixture of WO_3_·H_2_O, V_2_O_5_ and (TBA)­OH in the minimum volume of DMF at 180 °C for 35 min according to eq 8 showed only the characteristic broadened peaks for **7** at 83.9 and 85.9 ppm (Figure S8). Crystals of (TBA)_3_ **7** obtained upon cooling were shown to be pure by ^51^V NMR, and further product was obtained after precipitation and crystallization to give an overall yield of 88%.

(TBA)_3_[NbW_5_O_19_] (TBA)_3_ **8** – WO_3_·H_2_O was treated with 0.6 mol-equivalents of (TBA)­OH in DMF and heated to 180 °C to give a solution which was treated with Nb­(OEt)_5_. The ^183^W NMR spectrum of the hot reaction mixture showed the presence of **8** together with significant amounts of **1** and [W_10_O_32_]^4–^ (Figure S9a). After further heating, the initial crystalline product formed upon cooling was shown to be pure (TBA)_3_ **8** (Figure S9b) and a second fraction obtained by precipitation was shown to contain **1** and [W_10_O_32_]^4–^ in addition to **8** (Figure S9c). We suspected that [W_10_O_32_]^4–^ was generated during the high-temperature base treatment so a modified, stepwise procedure was used for the synthesis of (TBA)_3_ **9** from WO_3_·H_2_O and Ta­(OEt)_5_ (see below).

(TBA)_3_[TaW_5_O_19_] (TBA)_3_ **9** – (TBA)_2_ **1** was prepared *in situ* from WO_3_·H_2_O and (TBA)­OH in a minimum volume of DMF at 180 °C prior to degradation with further (TBA)­OH at room temperature, treatment with^17^O-enriched H_2_O and then reaction with Ta­(OEt)_5_ according to the overall stoichiometry shown in eq 10 After stirring overnight, (TBA)_3_ **9** was obtained in ca. 85% yield by precipitation with diethyl ether, and the ^17^O NMR spectrum (Figure S10a) showed >90% purity with the presence of a small amount of **1** and minor peaks that may be due to [Ta_2_W_4_O_19_]^4–^. The latter was removed by recrystallization, although ∼4% (TBA)_2_ **1** impurity remained (Figure S10b). The product showed a low intensity peak for Ta=O at 666 ppm, confirming the previously reported poor enrichment at this site.^20^

### Modified Method for MOCl_3_ Heterometal Precursors (M = V, Nb)

Treatment of (TBA)_2_ **1** with 5.2 mol-equivalents of base (eq 10) gives a precursor solution with a higher effective n/x for reaction with MOCl_3_ as in eq 12 This approach produces (TBA)Cl as a byproduct, and the Lewis acidity of MOCl_3_ requires removal of water produced during the degradation step in order to avoid the formation of HCl and protonation reactions to give (TBA)_2_ **1**, which may be mitigated by using pyridine as solvent.

11 $$5{[W_{6}O_{19}]}^{2-}{+26\mathrm{OH}}^{-}\to 6{[W_{5}O_{18}]}^{6-}{+13H}_{2}O$$

12 $${[W_{5}O_{18}]}^{6-}{+\mathrm{MOCl}}_{3}\to {[{\mathrm{MW}}_{5}O_{19}]}^{3-}{+3\mathrm{Cl}}^{-}$$

This method gave a 75% yield of (TBA)_3_ **7** from VOCl_3_ and a 67% yield of (TBA)_3_ **8** from [NbOCl_3_(MeCN)_2_]. These oxychlorides are readily obtained from V_2_O_5_ or NbCl_5_ and represent convenient alternatives to the alkoxido precursors, which may be advantageous for other heterometals.

### Homologous Methoxido {M^IV^W_5_} Reactivities (M = Ti, Sn, Zr, Hf)

Improved access to (TBA)_6_[{(MeO)­MW_5_O_18_}_2_] (M = Zr, **5**; Hf, **6**) facilitated systematic protonolysis reactions of these dimeric second and third row Group 4 {MW_5_} derivatives for comparison with those of monomeric [(MeO)­MW_5_O_18_]^3–^ (M = Ti, **3**; Sn, **4**). Our previous studies of **3** and **4** revealed the generally faster kinetics for protonolysis of SnOMe vs TiOMe,^6^ ^(d)^ and we were interested to determine the extent to which the higher coordination number for Zr and Hf in dimeric **5** and **6** might affect the kinetics of such reactions.

Kinetic plots for CD_3_OD exchange with **5** and **6** at 298 K are shown in Figure 12 and variable-temperature plots for (TBA)_6_ **6** are shown in Figure S11. Least squares fitting of these plots gave rates for the forward reaction *k*_f_ of 2.03 × 10^–2^ and 1.11 × 10^–2^ *M* ^–1^ s^–1^ for **5** and **6** respectively and rates for the reverse reaction *k*_b_ of 2.59 × 10^–2^ and 1.81 × 10^–2^ *M* ^–1^ s^–1^ respectively, with *K* = 0.787 and ΔG^0^ = 0.59 kJ mol^–1^ for **5** and *K* = 0.612 and ΔG^0^ = 1.22 kJ mol^–1^ for **6**. These values are compared with those for the monomeric titanium analogue **3** and the Keggin anions [(MeO)­MPW_11_O_39_]^4–^ (M = Ti, Sn) in Table 1,^6d^ which shows that equilibrium constants *K* for dimeric **5** and **6** are larger than monomeric **3**, while forward and backward rates *k*_f_ and *k*_b_ are greater for Zr than for Hf. Variable temperature ^1^H NMR experiments enabled the determination of forward and reverse activation energies of 47.0 (±3.9) and 44.7 (±5.0) kJ mol^–1^ respectively for **6** from Arrhenius plots (Figure S12).

**Figure 12:**
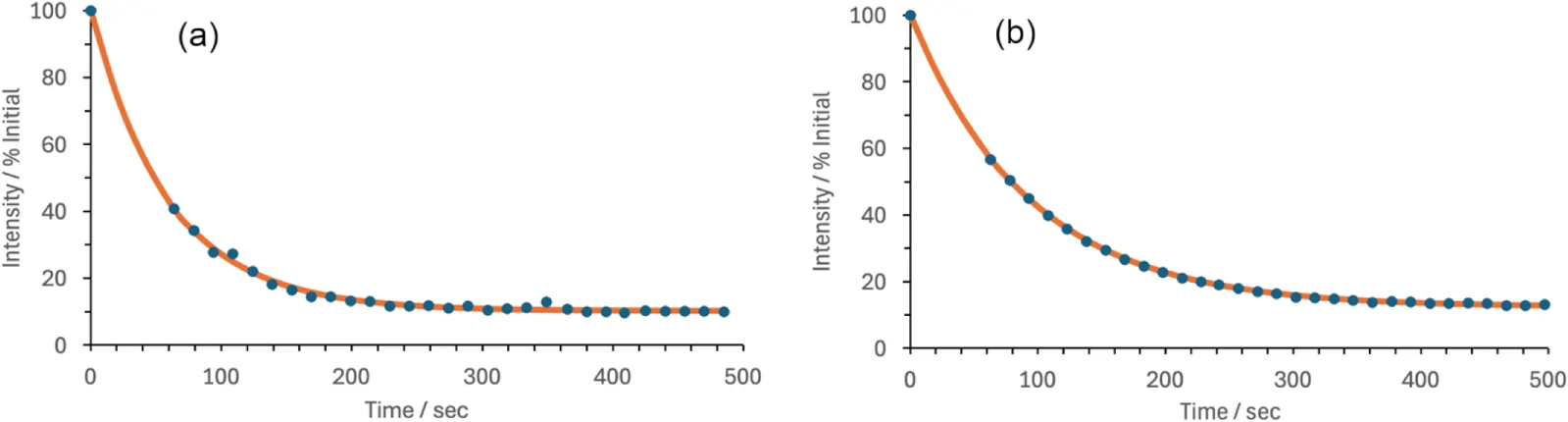
^1^H NMR kinetic plots for CD_3_OD exchange with (TBA)_6_ **5** (a) and (TBA)_6_ **6** (b) at 298 K. Least squares fits are shown by the solid lines.

**Table 1:** Kinetic Parameters from Analysis of NMR Data for Exchange of CD_3_OD with Methoxido-Substituted POMs^a^

Reaction	*K*	*k*_f_/*M* ^–1^ s^–1^	*k*_b_/*M* ^–1^ s^–1^	ΔG^0^ @ 298 K/kJ mol^–1^	Ref
{MeOZrW_5_}_2_ + 11.0 CD_3_OD	7.87 × 10^–1^	2.03 × 10^–2^	2.59 × 10^–2^	0.59	this work
{MeOHfW_5_}_2_ + 11.0 CD_3_OD	6.12 × 10^–1^	1.11 × 10^–2^	1.81 × 10^–2^	1.22	this work
{MeOTiW_5_} + 3.9 CD_3_OD	3.3 × 10^–1^	2.0 × 10^–2^	6.1 × 10^–2^	2.78	ref 6d
{MeOTiPW_11_} + 9 CD_3_OD	4.2 × 10^–1^	4.1 × 10^–4^	9.8 × 10^–4^	2.14	ref 6d
{MeOSnPW_11_} + 9.5 CD_3_OD	4 × 10^–1^	3 × 10^–2^	8 × 10^–2^	2.13	ref 6d

a *K* denotes the equilibrium constant, *k*_f_ the forward second order rate constant and *k*_b_ the backward (reverse) rate constant.

The exchange process for **5** and **6** was expected to be more complex than that for monomeric **3** because of the presence of two μ_2_-OCH_3_ groups in the dimeric anions. DFT calculations for methanol-methoxide exchange in the dimeric anion **5** showed that exchange proceeds *via* approach of MeOH to one of the Zr atoms then insertion into one of the Zr­(OMe)Zr bridges to form a six-membered ring that facilitates the proton transfer from the methanol to the methoxide groups (Figure S13). The energy barrier of 84.1 kJ mol^–1^ is comparable to the energy barriers for hydrolysis reported in our previous publication (Table S3).^6d^ Analogous calculations for methanol exchange with **6** have yet to provide the transition state structure for the Hf analogue.

### Protolytic Ligand Metathesis Reactions

Protonolysis of monomeric alkoxido {(RO)­MW_5_} (M = Ti, Sn) derivatives provides useful access to a range of substituted {LMW_5_} Lindqvist type POMs. We previously showed that the aryloxido ligands introduced in reactions between phenols and dimeric **5** are too bulky to bridge between {ZrW_5_} units and give monomeric derivatives containing six-coordinate Zr, while CH_3_CO_2_H or acacH react to give monomeric chelate derivatives [(AcO)­ZrW_5_O_18_]^3–^ **10** and [(acac)­ZrW_5_O_18_]^3–^ **11** which contain seven-coordinate Zr.^6b^ We have now prepared the monomeric benzoates (TBA)_3_[(PhCO_2_)­ZrW_5_O_18_] (TBA)_3_ **12** and (TBA)_3_[(PhCO_2_)­HfW_5_O_18_] (TBA)_3_ **13** as well as (TBA)_3_[(acac)­HfW_5_O_18_] (TBA)_3_ **14** from (TBA)_6_ **6** for comparison. NMR and FTIR spectra are shown in Figures S14–S19 and ^17^O NMR data are collected in Table 2 together with those for (TBA)_3_[(acac)­ZrW_5_O_18_]. Our previous studies showed that the electronic properties of monomeric {MW_5_} methoxido anions with 6-coordinate M depend markedly on the nature of M.^6c^ Hence, in **3** and **4** where the Sn–OMe bond is more ionic than Ti–OMe, the different charge distributions across the tungstate framework are reflected in the ^17^O NMR chemical shifts, which are sensitive to the strength of metal-oxido bonding. The variations in chemical shifts from those of **3** therefore provide some insight into the effects of the heterometal M and its coordination geometry as well as the influence of the ligand bound to M in {LMW_5_O_18_} Lindqvist anions.

**Table 2:** ^17^O and ^183^W NMR Data for {M^IV^W_5_} Anions [(MeO)­MW_5_O_18_]^3–^ (M = Ti, **3**; M = Sn, **4**), [{(MeO)­MW_5_O_18_}_2_]^6–^ (M = Zr, **5**; M = Hf, **6**), [(PhCO_2_)­MW_5_O_18_]^3–^ (M = Zr, **12**; Hf, **13**), and [(acac)­MW_5_O_18_]^3–^ (M = Zr, **11**; M = Hf **14**)

	δ_O_/ppm					δ_W_/ppm	
Anion	W_eq_=O	W_ax_=O	MOW	WOW	μ_6_-O	W_eq_	W_ax_
**3**	723	716	527	393, 382	–56	32.3	64.5
**4**	721	685	397	384, 364	19	76.9	–128.1
**5**	713	692	486	387, 379	–57	37.6	64.4
**6**	711	689	464	386, 379	–61	54.7	68.7
**12**	723	705	478	392, 382	–50	42.0	77.9
**13**	721	700	455	391, 381	–53	45.4	65.5
**11** ^a^	714	697	483	387, 377	–58	38.8	63.8
**14**	714	695	462	388, 377	–60	52.1	62.0

a Ref 6b.

^17^O chemical shifts for W_eq_=O, W_ax_=O and MOW_eq_ for dimeric methoxides **5** and **6**, which contain 7-coordinate Zr and Hf, are shifted notably upfield from those of the monomeric {TiW_5_} methoxide **3**. The shielding effect and chemical shift deviations from the {TiW_5_} values for the W_ax_=O and MOW sites are greatest for {HfW_5_} **6**. For the Zr and Hf {MW_5_} methoxide (**5**,**6**) benzoate (**12**,**13**) and acac (**11**,**14**) derivatives, MOW chemical shifts are affected much more by the identity of the heterometal than those for the other oxygen sites, and HfOW peaks appear about 20 ppm upfield of ZrOW. In contrast, the nature of the ligand bonded to Zr or Hf affects the ^17^O chemical shifts of all oxygen sites associated with the {W_5_O_18_} framework. For a given M (M = Zr, Hf), peaks in the ^17^O NMR spectra of {(MeO)­MW_5_} and {(acac)­MW_5_} anions have similar shifts, but the W=O, WOW and μ_6_-O peaks are upfield and MOW peaks are downfield of those for the {(PhCO_2_)­MW_5_} anions. The correlation between δ_O_ and M–O bond strength, which is associated with the degree of M–O π-bonding, is well established,^13,21,22^ and the plot of weighted mean δ_W=O_ versus ν­(W=O) for terminal W=O of transition-metal substituted {LMW_5_} Lindqvist anions (Figure 13) reveals the effects of heterometal M and ligand L on terminal W–O bond strength. The dimeric Zr and Hf methoxides **5** and **6** and the monomeric Zr and Hf acac derivatives **11**and **14** appear to have the weakest W=O bonds, followed by the monomeric Zr and Hf benzoates **12** and **13** and the monomeric Ti methoxide **3**, while [M^V^W_5_O_19_]^3–^ anions **7**, **8**, and **9** have stronger W=O bonds than the anions containing {LM^IV^W_5_O_18_}^3–^ Lindqvist units. As expected, the strongest W=O bonds are associated with the dianion **1**. Given that the maximum errors for weighted δ_W=O_ and ν­(W=O) are ±1 ppm and ±3 cm^–1^ respectively, the deviation of the main group derivative **4** with weighted δ_W=O_= 714 ppm and ν­(W=O) = 950 cm^–1^ from the trend line in Figure 13 is likely due to the different heterometal atomic orbital contributions to the frontier molecular orbitals, although a detailed analysis is beyond the scope of this paper. It is worth noting that the ^183^W NMR data do not show a similar trend.

**Figure 13:**
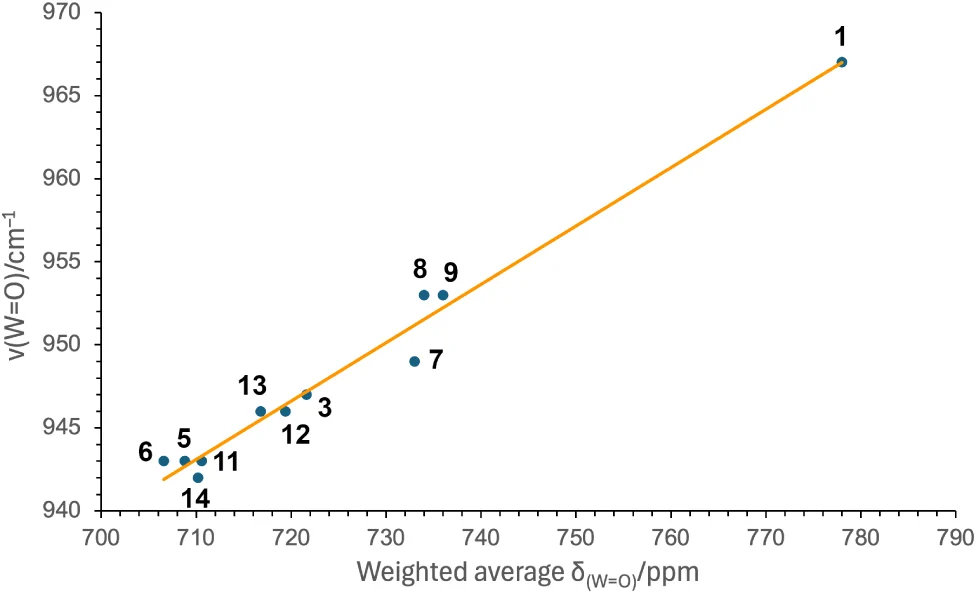
Correlation between the weighted mean δ_W=O_ and ν­(W=O) for terminal W=O of **1** and {MW_5_} transition metal substituted Lindqvist anions **3**, **5**, **6**, **7**, **8**, **9**, **11**, **12**, **13** and **14**.

X-ray structures of the anions **12**, **13**, and **14** are shown in Figure S20 and associated crystallographic details are given in Table S4. The mean bond distances and angles shown in Table S5 are similar for these monomeric anions, apart from the angles subtended by the ligands, which are greater for acac than O_2_CPh.

### Comparative Cyclic Voltammetry

Cyclic voltammograms of TBA salts of **1** and the M^IV^-substituted anions **3**, **4**, **5** and **6** as 3 m*M* solutions in MeCN are compared with those of analogous solutions of M^V^-substituted anions **7**, **8** and **9** in Figure 14. The redox couples at ca. −2.3 V vs Ag/AgNO_3_ for **3** and **4** and *ca*. −2.1 for **8** and **9** suggests that the one-electron reduced anions [(MeO)­MW_5_O_18_]^4–^ (M = Ti, Sn) and [MW_5_O_19_]^4–^ (M = Nb, Ta) should be chemically accessible. The significantly more negative potential than the first redox couple for **1** is consistent with the higher charge for these anions. In stark contrast, 3 m*M* solutions of **5** and **6** (i.e., with twice the heterometal concentration) show no redox events within the potential window. The relatively positive reduction potential of **7** is associated with the presence of a low-energy valence 3d orbital at the vanadium center, whereas the reduction events of the other {MW_5_} anions are associated with tungsten-based orbitals. We have previously described the one-electron-reduced **7** [VW_5_O_19_]^4–^ and its protonated derivative [VW_5_O_19_H]^3–^, together with DFT calculations that reveal how metal substitution affects LUMO energies and, consequently, reduction potentials.^23^ Calculated and observed first reduction potentials for **1**, **3**, **4**, **5**, **6**, **7**, **8** and **9** are shown in Table 3. The calculated potentials for **5** and **6** are −2.640 and −2.663 V vs Ag/AgNO_3_, which lie outside the solvent potential window. Consequently, no redox events are observed in the voltammograms of **5** and **6** shown in Figure 14.

**Figure 14:**
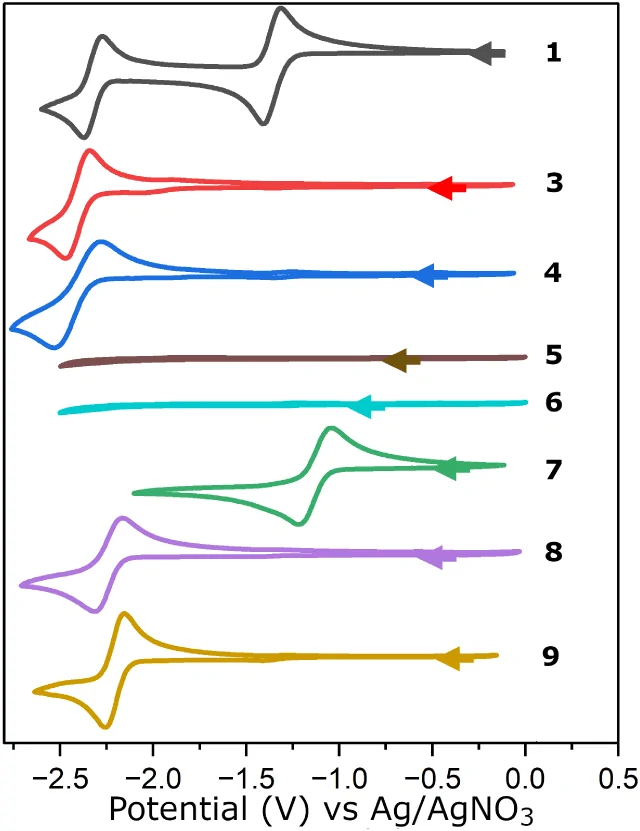
Cyclic voltammograms of 3 m*M* solutions of (TBA)_2_ **1**, (TBA)_3_[(MeO)­MW_5_O_18_] (**3** and **4**) (TBA)_6_[{(MeO)­MW_5_O_18_}_2_] (**5** and **6**) and (TBA)_3_[MW_5_O_19_] (**7**, **8**, **9**) in MeCN with 0.1 *M* (TBA)­PF_6_ supporting electrolyte, glassy carbon working electrode, Pt counter-electrode, Ag/AgNO_3_ quasi reference electrode and 100 mV s^–1^ scan rate. Arrows indicate rest potentials.

**Table 3:** Calculated and Observed First Reduction Potential E^0^ (V vs Ag/AgNO_3_) for [W_6_O_19_]^2–^ **1**, [(MeO)­MW_5_O_18_]^3–^ (M = Ti, **3**; M = Sn, **4**), [{(MeO)­MW_5_O_18_}_2_]^6–^ (M = Zr, **5**; M = Hf, **6**) and [MW_5_O_19_]^3–^ (M = V, **7**; Nb, **8**; Ta, **9**), Where E^0^ = (E_pa_ + E_pc_)/2

Anion	E^0^ (calc)	E^0^ (obs)
**1**	–1.292	–1.25
**3**	–2.287	–2.34
**4**	–2.358	–2.35
**5**	–2.640	–
**6**	–2.663	–
**7**	–0.789	–1.02
**8**	–2.101	–2.09
**9**	–2.073	–2.05

## Conclusions

We have developed a simple and efficient synthesis of hexatungstate (TBA)_2_ **1** from WO_3_·H_2_O and showed that M­(IV) and M­(V) substituted {MW_5_} Lindqvist anions can be obtained conveniently in nonaqueous solvents from either (TBA)_2_ **1** or WO_3_·H_2_O. By addition of the appropriate amount of (TBA)­OH, we were able to tune the anionic charge per W (n/x) of the putative {W_*x*_O_*y*_H_*z*_}^n–^ reactive intermediate to that previously achieved by mixing the appropriate amounts of (TBA)_2_WO_4_ and WO­(OMe)_4_. By eliminating the need to prepare WO­(OMe)_4_, this approach is significantly more convenient than our previously reported procedure, and is readily scaled. The improved access to this family of POMs will enable more extensive reactivity studies, and the investigations described here provide further insight into the influence of heteroatoms within POM structures on reactivity – an essential requirement for the design of POM-based catalysts.

In forthcoming publications, we will describe detailed spectroscopic investigations of speciation in the tungstate precursor solutions obtained by base degradation of (TBA)_2_ **1** and show how this approach provides access to an extensive range of other {MW_5_} species.

## Supplementary Material



## Notes

Details of general procedures, NMR and FTIR spectra, X-ray crystal structures, kinetic plots, and computational results (PDF)

## ⊥

Department of Chemistry, University of Rochester, Rochester, NY 14627, United States

## ∇

37 Peacock Drive, Caldecote, Cambridge CB23 7FE
