# Supplementary material for: M(IV)- and M(V)-Substituted {MW5} Lindqvist-Type Polyoxometalates from Plenary [W6O19]2– or WO3·H2O: A Gateway to Wider Systematic Reactivity Studies
Source: Inorg Chem. 2026 Oct 14;65(42):24883–96. doi: 10.1021/acs.inorgchem.6c03884 (PMC13715581; doi:10.1021/acs.inorgchem.6c03884)
Supplement: Supplementary file 1 [file ic6c03884_si_001.pdf]

Supporting Information

M(IV)- and M(V)-substituted {MW_5} Lindqvist-type polyoxometalates from plenary $[W_6O_{19}]^{2-}$ or $WO_3.H_2O$: a gateway to wider systematic reactivity studies.

Dominic Shiels,[a] Daniel Lebbie,[a] Amar Mohammed,[a] Magda Pascual-Borràs,[a, b] Rosie Black,[a] Corinne Wills,[c] Paul G. Waddell,[c] Benjamin R. Horrocks[c] and R. John Errington[a]*

[a] NUPOM Lab, Chemistry, School of Natural and Environmental Sciences, Newcastle University, Newcastle upon Tyne, NE1 7RU, UK.

[b] Departament de Química Inorgànica i Orgànica, Institut de Química Teòrica i Computacional, Universitat de Barcelona, 08028 Barcelona, Spain

[c] Chemistry, School of Natural and Environmental Sciences, Newcastle University, Newcastle upon Tyne, NE1 7RU, UK.

Corresponding Author Contact Information:
R. John Errington: john.errington@newcastle.ac.uk

Contents

		page
General experimental details.		S2
**Figure S1.**	^{183}W NMR spectra of the solution obtained by treatment of $WO_3.H_2O$ with 2 mole-equivalents of (TBA)OH in DMF after heating for 15 minutes at 180 °C (a), and the crystallised $(TBA)_2$**1** obtained from the solution (b). Spectra recorded at 100 °C.	S4
**Figure S2.**	^1H NMR spectra of $(TBA)_3$**2** (a) and $(TBA)_3$**3** (b).	S5
**Figure S3.**	^{183}W NMR spectrum of $(TBA)_3[(MeO)TiW_5O_{18}]$, $(TBA)_3$**3** showing $^2J(^{183}W_{ax}{}^{183}W_{eq})$.	S5
**Figure S4.**	^{17}O, ^{119}Sn and ^1H NMR spectra recorded during the synthesis of $(TBA)_3[(MeO)SnW_5O_{18}]$ $(TBA)_3$**4**.	S6
**Figure S5.**	^{17}O NMR spectra of products from treatment of base-degraded $(TBA)_2$**1** with $[\{Zr(O^iPr)_4(^iPrOH)\}_2]$ before (a) and after (b) treatment with MeOH, and ^{17}O (c) and ^1H (d) NMR spectra of recrystallised $(TBA)_6[\{(MeO)ZrW_5O_{18}\}_2]$ $(TBA)_6$**5**.	S7
**Table S1.**	Crystallographic parameters for $(TBA)_6[\{(MeO)MW_5O_{18}\}_2]$ (M = Zr, **5**; M = Hf, **6**).	S8
**Table S2.**	Average bond distances (Å) and angles (°) for anions **5** and **6**.	S8
**Figure S6.**	^{51}V NMR spectrum of the reaction mixture in the synthesis of $(TBA)_3$**7** from base-degraded $(TBA)_2$**1** and V_2O_5 with inset showing the temperature-dependence of the ^{51}V chemical shift (a) and of the ^{17}O NMR spectrum of the precipitated product (b).	S9
**Figure S7.**	Expansion of ^{17}O NMR spectrum of $(TBA)_8$**8** obtained from base-degraded $(TBA)_2$**1** and $Nb(OEt)_5$ showing impurity peaks at 695, 436 and 376 ppm assigned to $[Nb_2W_4O_{19}]^{4-}$.	S9
**Figure S8.**	^{183}W NMR spectrum of the solution obtained by heating a mixture of $WO_3.H_2O$, V_2O_5 and (TBA)OH (10:1:6 mole-ratio) in DMF at 180 °C for 35 minutes, recorded at 100 °C (a) and ^{51}V NMR spectrum of the crystalline product in MeCN (b).	S10
**Figure S9.**	^{183}W NMR spectrum of the solution obtained by treating the product from the reaction between $WO_3.H_2O$ and (TBA)OH with $Nb(OEt)_5$ (5:3:1 mole-ratio) in DMF at 180 °C for 30 minutes, recorded at 60°C (a), ^{17}O NMR spectrum of crystals obtained upon cooling after heating for 3 hours (b) and ^{183}W and ^{17}O NMR spectra of the solid obtained from the mother liquor by precipitation with diethyl ether (c).	S10

Figure S10. ^{17}O NMR spectra of the product obtained by (i) treatment of $WO_3.H_2O$ with 0.34 mole-equivalents of (TBA)OH in DMF at 180 °C for 10 min, (ii) addition of 0.27 mole-equivalents of methanolic (TBA)OH and 1.29 mole-equivalents of ^{17}O-enriched H_2O, (iii) reaction with 0.22 mole-equivalents of $Ta(OEt)_5$ at 20 °C overnight, (iv) precipitation of the product with diethyl ether (a) and recrystallisation from MeCN (b). S11

Figure S11. Variable temperature ^1H NMR kinetic plots for exchange reactions between $(TBA)_6$**6** and CD_3OD with least squares fits shown as orange lines. S12

Figure S12. Arrhenius plots obtained from kinetic data in Figure S11 for the forward and backward exchange reactions between $(TBA)_6$**6** and CD_3OD. S13

Figure S13. Calculated transition state for exchange reaction between $(TBA)_6$**5** and MeOH, highlighting the coordination environments of the Zr atoms. S13

Table S3. Comparison of calculated activation energies E_a ($kcal.mol^{-1}$) for hydrolysis of $\{(MeO)MW_5\}$ Lindqvist anions.

Figure S14. ^1H (a) and ^{17}O (b) NMR spectra of $(TBA)_3[(PhCO_2)ZrW_5O_{18}]$ $(TBA)_3$**12**. S14

Figure S15. ^1H (a) and ^{17}O (b) NMR spectra of $(TBA)_3[(PhCO_2)HfW_5O_{18}]$ $(TBA)_3$**13**. S15

Figure S16. ^1H (a), ^{13}C (b) and ^{17}O (c) NMR spectra of $(TBA)_3[(acac)HfW_5O_{18}]$ $(TBA)_3$**14**. S16

Figure S17. ATR FTIR spectra of $(TBA)_6[\{(MeO)MW_5O_{18}\}_2]$: M = Zr, **5** (a) and Hf, **6** (b). S17

Figure S18. ATR FTIR spectra of $(TBA)_3[(PhCO_2)MW_5O_{18}]$: M = Zr, **12** (a) and Hf, **13** (b). S18

Figure S19. ATR FTIR spectra of $(TBA)_3[(acac)MW_5O_{18}]$: M = Zr, **11** (a) and Hf, **14** (b). S19

Figure S20. X-ray crystal structures of the anions $[(PhCO_2)ZrW_5O_{18}]^{3-}$ **12** (a), $[(PhCO_2)HfW_5O_{18}]^{3-}$ **13** (b) and $[(acac)HfW_5O_{18}]^{3-}$ **14** (c). S20

Table S4. Crystallographic parameters for $(TBA)_3$**12**, $(TBA)_3$**13** and $(TBA)_3$**14**. S20

Table S5. Averaged bond distances (Å) and angles (°) for the monomeric anions $[(PhCO_2)ZrW_5O_{18}]^{3-}$ **12**, $[(PhCO_2)HfW_5O_{18}]^{3-}$ **13**, $[(acac)ZrW_5O_{18}]^{3-}$ **11** and $[(acac)HfW_5O_{18}]^{3-}$ **14**. S21

References S21

General experimental details

All reactions and manipulations were carried out under an atmosphere of dry, oxygen-free nitrogen in screw-top flasks fitted with J Young PTFE screw valves using Schlenk and dry-box techniques.[1]

Caution! Extreme care should be taken both in the handling of the cryogen liquid nitrogen and its use in the Schlenk line trap to avoid the condensation of oxygen from air.

Diethyl ether was dried over and distilled from sodium benzophenone ketyl, acetonitrile was dried over and distilled from calcium hydride, and methanol was pre-dried over 3A molecular sieves and distilled from magnesium methoxide. Dimethylsulfoxide and deuterated solvents were dried over activated 3A molecular sieves and then degassed. All solvents were stored over activated 3A molecular sieves under dry nitrogen. 40% ^{17}O-enriched water and deuterated solvents were purchased from Goss Scientific. $Sn(O^tBu)_4$ and $VO(O^tBu)_3$ were prepared from $SnCl_4$ or V_2O_5 respectively by treatment with ^tBuOH using procedures based on literature methods.[2, 3] $[\{Hf(O^iPr)_4(^iPrOH)\}_2]$ was prepared by treatment of $HfCl_4$ in THF with ^iPrOH and

ammonia gas, based on the original procedure reported by Bradley *et al.*[4] $Ti(O^iPr)_4$ was supplied by Tioxide UK and distilled before use, $[\{Zr(O^iPr)_4(^iPrOH)\}_2]$ and $[\{Ta(OEt)_5\}_2]$ were supplied by Epivalence. $[\{Nb(OEt)_5\}_2]$, WO_3H_2O and 1 *M* (TBA)OH in MeOH were purchased from Aldrich.

Spectroscopy – FTIR spectra were recorded on a Bruker Alpha spectrometer fitted with a Platinum ATR module (4000 - 400 cm^{-1}). NMR spectra were recorded on a Bruker Avance III 300 spectrometer operating at 300.0 MHz (^1H), 40.67 MHz (^{17}O); a Bruker Avance 400 spectrometer operating at 399.78 MHz (^1H), 54.20 MHz (^{17}O), or a Bruker Avance III HD 500 spectrometer operating at 500.15 MHz (^1H), 67.84 MHz (^{17}O), 20.84 MHz (^{183}W). NMR spectra were referenced by sample replacement to $SiMe_4$ (^1H and ^{13}C), H_2O (^{17}O) and aqueous 2 *M* Na_2WO_4 (^{183}W). ^1H and ^{13}C NMR resonances due to $^nBu_4N^+$ (TBA) cations are not listed in NMR data given below, but appear as multiplets centred at about 1.0, 1.4, 1.7 and 3.2 ppm in ^1H NMR spectra and as singlets at about 13, 19, 24 and 58 ppm in $^{13}C\{^1H\}$ NMR spectra. For the kinetic experiments, an accurately weighed amount of the POM was added to a 5 mm NMR tube in a glovebox and dissolved in a measured amount of CD_3CN. An ^1H NMR spectrum was recorded prior to addition of a measured amount of CD_3OD. The time of addition was noted and spectra were then acquired at intervals. Delay times for ^1H NMR spectra were adjusted to allow for the previously determined T_1 values for MOMe protons.

Electrochemistry – Cyclic voltammograms of 3 m*M* POM solutions in 0.1 *M* $(TBA)PF_6$ supporting electrolyte were recorded in an argon-filled Braun glovebox using a PalmSens*4* potentiostat, glassy carbon working electrode, Pt wire counter-electrode and $Ag/AgNO_3$ quasi reference electrode with a scan rate of 100 mVs^{-1}.

X-ray crystallography – Single crystal diffraction data for $(TBA)_6[\{(MeO)ZrW_5O_{18}\}_2]$ $(TBA)_6$**5**, $(TBA)_6[\{(MeO)HfW_5O_{18}\}_2]$ $(TBA)_6$**6** $(TBA)_3[(PhCO_2)HfW_5O_{18}]$ $(TBA)_3$**13** and $(TBA)_3[(acac)HfW_5O_{18}]$ $(TBA)_3$**14** were collected on an XtaLAB Synergy HyPix-Arc 100 diffractometer using copper radiation ($\lambda_{CuK\alpha}$ = 1.54184 Å). Data for $(TBA)_3$**12** were collected on an Xcalibur, Atlas, Gemini ultra diffractometer using copper radiation ($\lambda_{CuK\alpha}$ = 1.54184 Å). All data were collected at 150 K using an Oxford Cryosystems CryostreamPlus open-flow N_2 cooling device. Intensities were corrected for absorption using a multifaceted crystal model created by indexing the faces of the crystal for which data were collected.[5] Cell refinement, data collection and data reduction were undertaken via the software CrysAlisPro.[6] All structures were solved using XT[7] and refined by XL[8] using the Olex2 interface.[9] All non-hydrogen atoms were refined anisotropically and hydrogen atoms were positioned with idealised geometry. The displacement parameters of the hydrogen atoms were constrained using a riding model with $U_{(H)}$ set to be an appropriate multiple of the U_{eq} value of the parent atom. All structures exhibit some degree of disorder, for which details of the refinement are available in the CIF.

Computation – DFT calculations were performed on a series of compounds using the Gaussian 09 suite of programs.[10] The minima (reactants, intermediates and products) as well as transition states were obtained using the B3LYP hybrid functional with a 6-31G** basis set.[11, 12] For heavy elements, we used the standard double-zeta basis set with LANL pseudopotentials of Hay and Wadt.[13] Transition states were identified by the characteristic calculation of Hessian matrices with only one imaginary frequency corresponding to the reaction coordinate. The calculations include solvent effects by means of the polarizable continuum model (PCM) to simulate the effects of acetonitrile.[14] Energies reported here correspond to the computed Gibbs

energies in solution. All reported potentials are referenced to the Ag/$AgNO_3$ in acetonitrile.[15] The energy of the proton reported by Truhlar *et al*. was employed in calculations. [16]

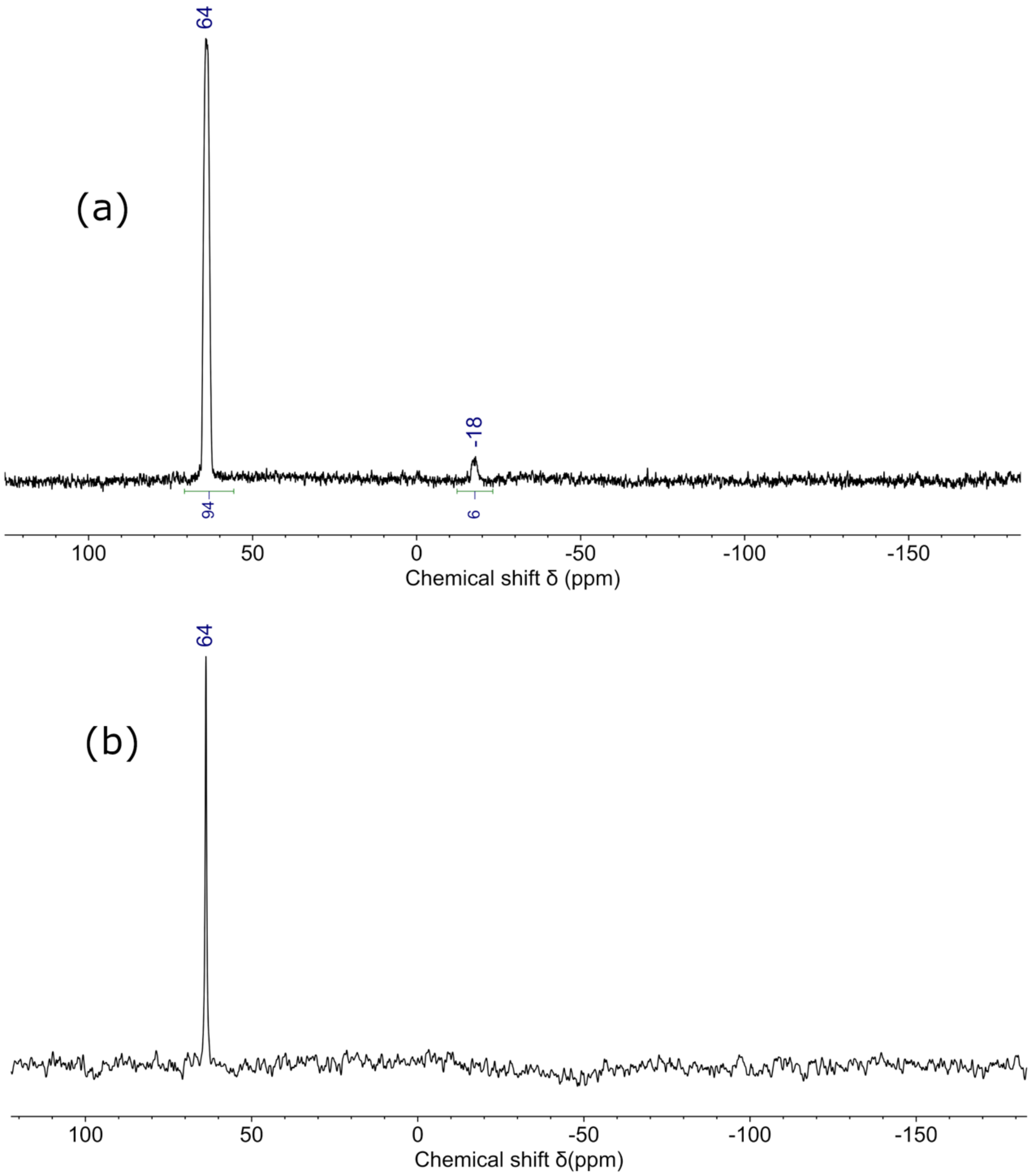

Figure S1. ^{183}W NMR spectra of the solution obtained by treatment of $WO_3.H_2O$ with 2 mole-equivalents of (TBA)OH in DMF after heating for 15 minutes at 180 °C (a), and the crystallised $(TBA)_2$**1** obtained from the solution (b). Spectra recorded at 100 °C.

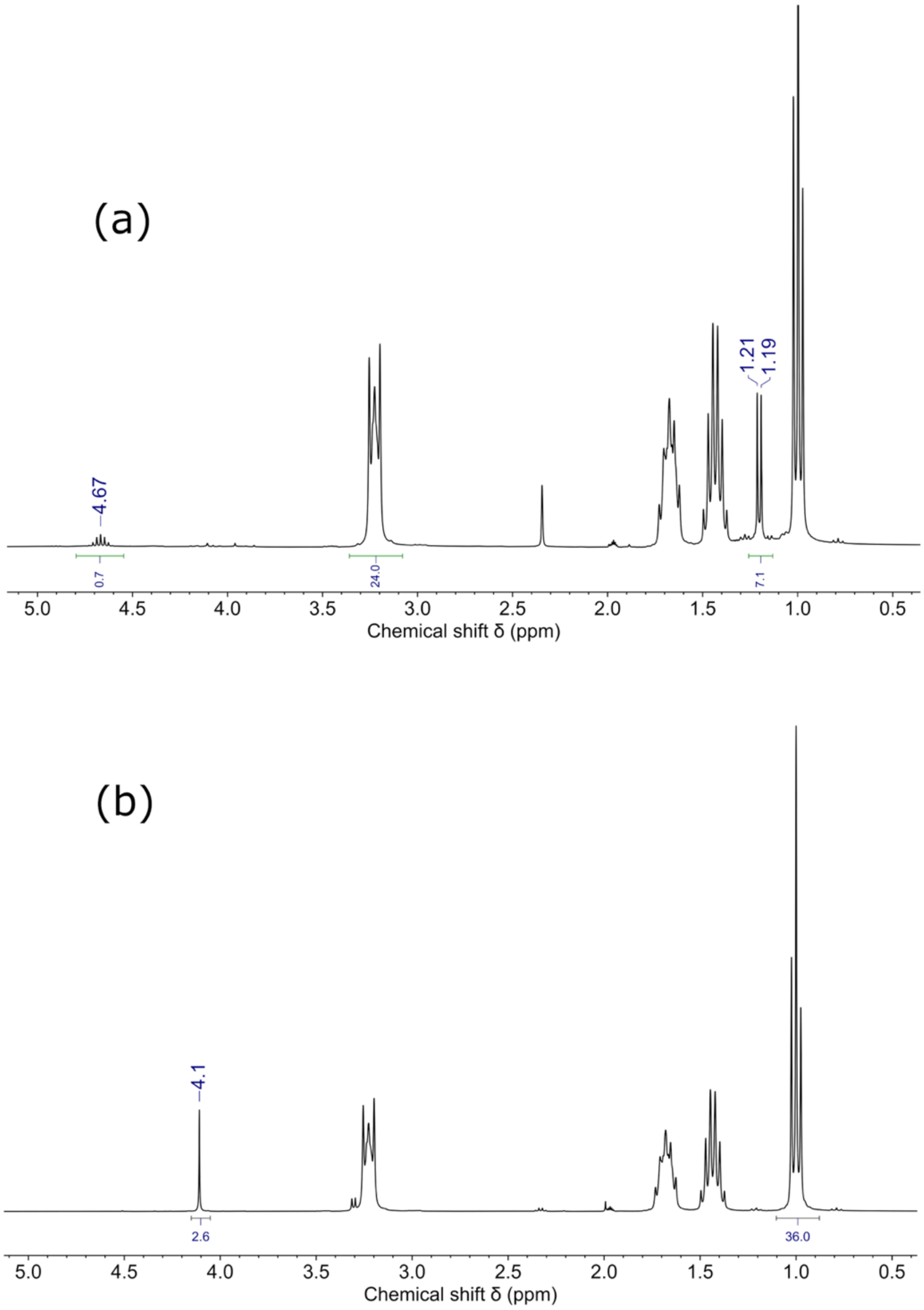

Figure S2. ^{1}H NMR spectra of $(TBA)_3[(^{i}PrO)TiW_5O_{18}]$ $(TBA)_3$**2** (a) and $(TBA)_3[(MeO)TiW_5O_{18}]$ $(TBA)_3$**3** (b).

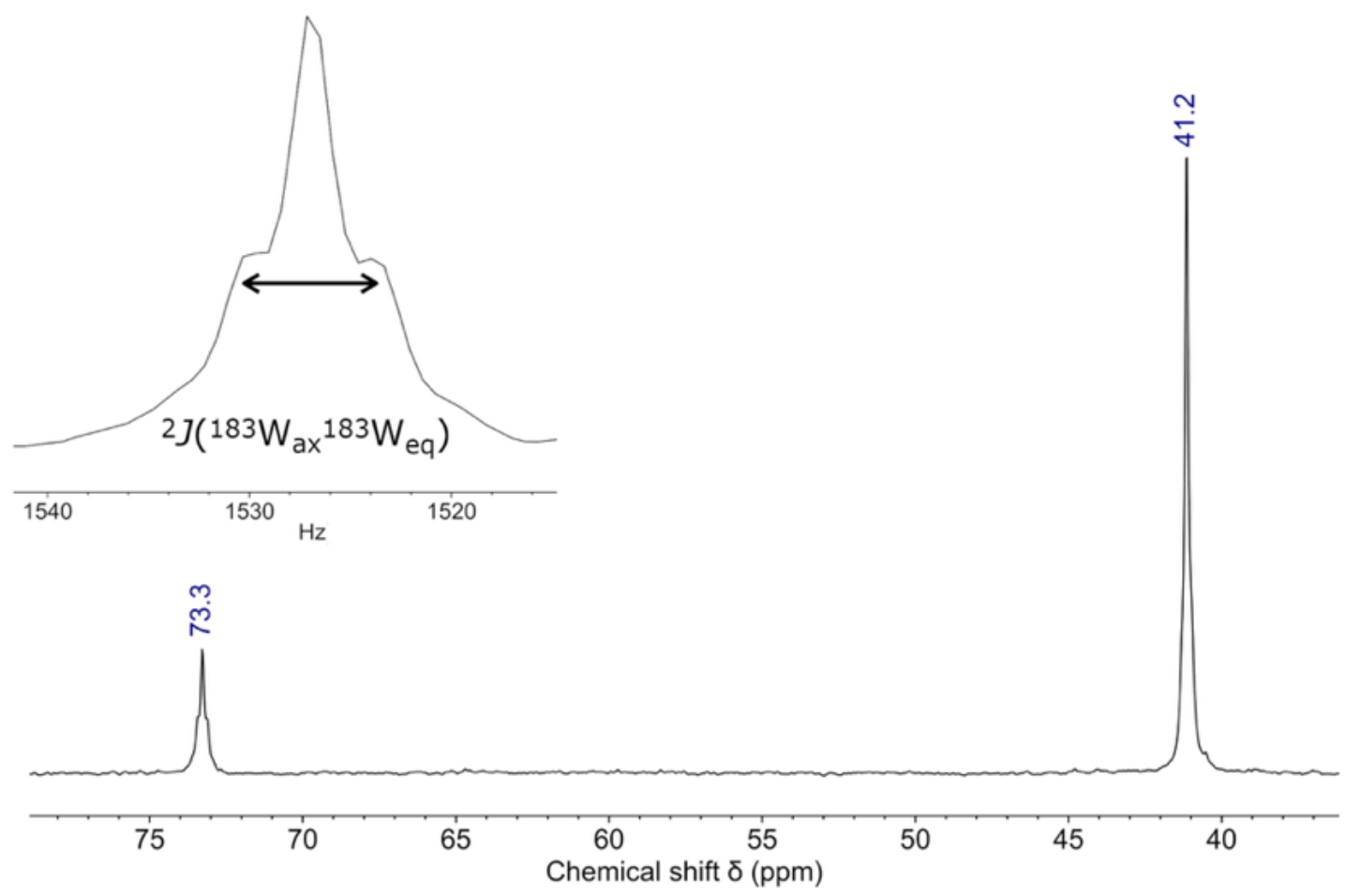

Figure S3. ^{183}W NMR spectrum of $(TBA)_3[(MeO)TiW_5O_{18}]$ $(TBA)_3$**3** showing $^{2}J(^{183}W_{ax}{}^{183}W_{eq})$.

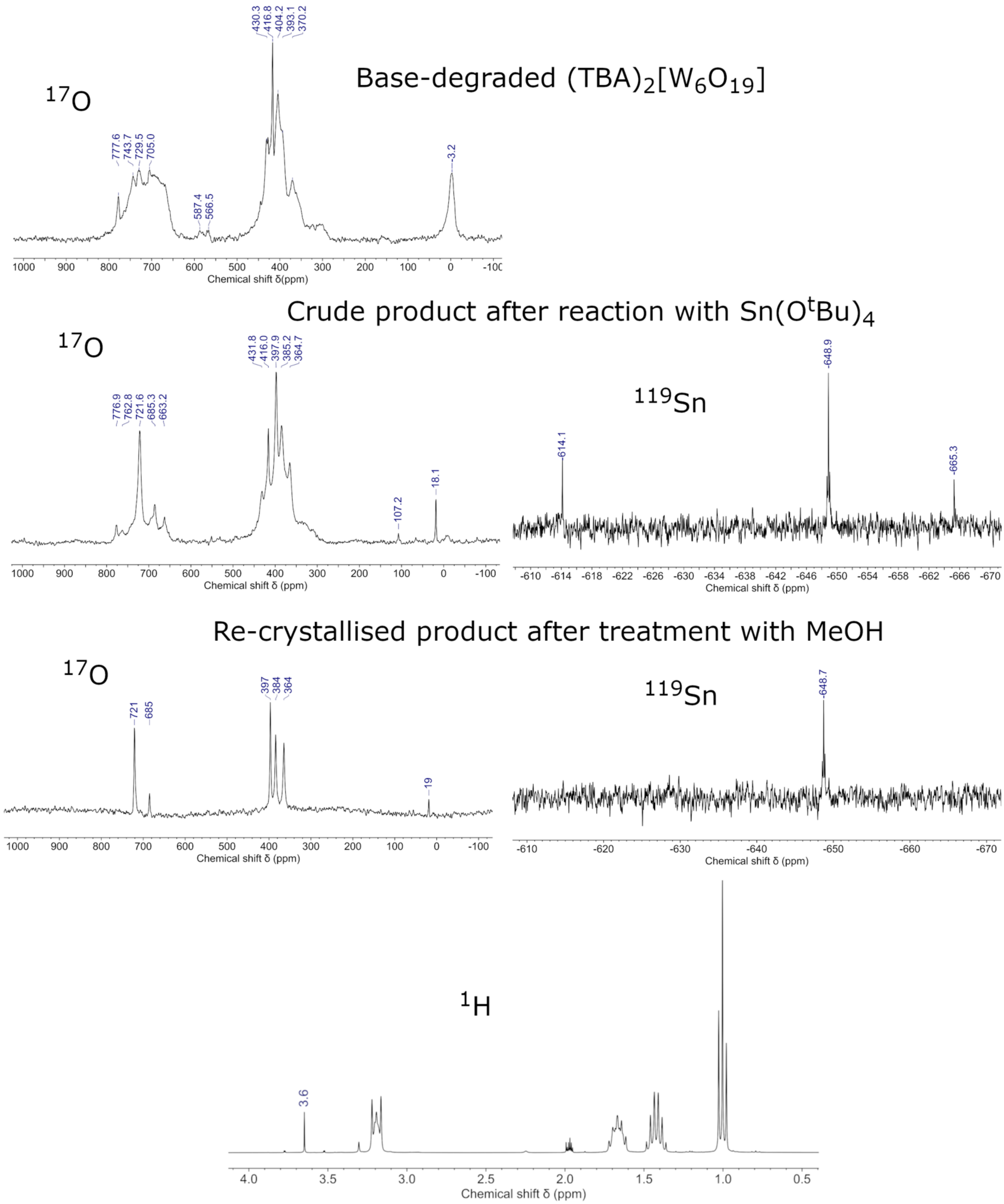

Figure S4. ^{17}O, ^{119}Sn and ^{1}H NMR spectra recorded during the synthesis of $(TBA)_3[(MeO)SnW_5O_{18}]$ $(TBA)_3$**4**.

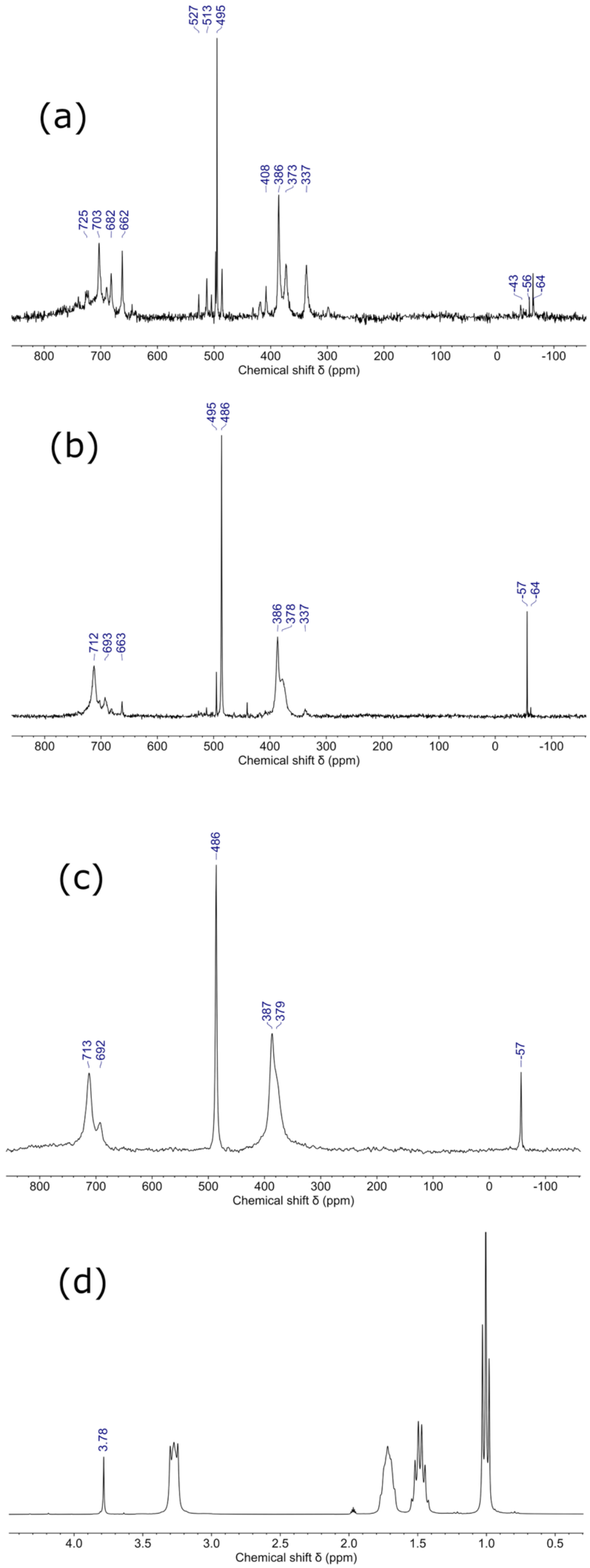

Figure S5. ^{17}O NMR spectra of products from treatment of base-degraded $(TBA)_2$**1** with $[\{Zr(O^iPr)_4(^iPrOH)\}_2]$ before (a) and after (b) treatment with MeOH, and ^{17}O (c) and ^1H (d) NMR spectra of recrystallised $(TBA)_6[\{(MeO)ZrW_5O_{18}\}_2]$ $(TBA)_6$**5**.

Table S1. Crystallographic parameters for $(TBA)_6[\{(MeO)MW_5O_{18}\}_2]$ (M = Zr, **5**; M = Hf, **6**)

	$(TBA)_6$**5**	$(TBA)_6$**6**
Empirical formula	$C_{110}H_{248}N_8O_{40}W_{10}Zr_2$	$C_{110}H_{248}Hf_2N_8O_{40}W_{10}$
Formula weight	4344.09	4518.63
Temperature/K	150.0(2)	150.0(2)
Crystal system	orthorhombic	orthorhombic
Space group	$Pca2_1$	$Pca2_1$
a/Å	34.3821(5)	34.3009(3)
b/Å	17.2735(2)	17.2598(2)
c/Å	25.4240(4)	25.4047(2)
α/°	90	90
β/°	90	90
γ/°	90	90
Volume/Å^3	15099.3(4)	15040.3(2)
Z	4	4
ρ_{calc}g/cm^3	1.911	1.996
μ/mm^{-1}	15.291	16.718
F(000)	8416.0	8672.0
Crystal size/mm^3	0.13 × 0.11 × 0.07	0.2 × 0.1 × 0.09
Radiation	CuKα (λ = 1.54184)	CuKα (λ = 1.54184)
2Θ range for data collection/°	5.152 to 155.284	5.152 to 155.284
Index ranges	$-36 \le h \le 41, -21 \le k \le 21, -30 \le l \le 30$	$-41 \le h \le 15, -20 \le k \le 20, -30 \le l \le 30$
Reflections collected	136581	64209
Independent reflections	27692 [R_{int} = 0.0366, R_{sigma} = 0.0262]	26734 [R_{int} = 0.0325, R_{sigma} = 0.0387]
Data/restraints/parameters	27692/2230/1740	26734/2053/1701
Goodness-of-fit on F^2	1.038	1.026
Final R indexes [I>=2σ (I)]	R_1 = 0.0396, wR_2 = 0.1042	R_1 = 0.0366, wR_2 = 0.0927
Final R indexes [all data]	R_1 = 0.0479, wR_2 = 0.1123	R_1 = 0.0457, wR_2 = 0.0992
Largest diff. peak/hole / e Å^{-3}	1.43/-1.94	1.19/-1.73
Flack parameter	0.188(13)	-0.023(11)

Table S2. Average bond distances (Å) and angles (°) for anions- **5** and **6**.

	**5** (M = Zr)	**6** (M = Hf)
M–OMe	2.148	2.131
M–OW_{eq}	2.115	2.097
M–μ_6O	2.366	2.352
O1MO2	67.2	66.7
M.....W_{eq}	3.361	3.369
W_{eq}–OM	1.842	1.839
W_{ax}–OM	2.415	2.417
W=O	1.720	1.719
W_{eq}–OW_{eq} (long)	1.956	1.955
W_{eq}–OW_{eq} (short)	1.929	1.925
W_{eq}–OW_{ax}	1.972	1.972
W_{eq}–μ_6O	2.340	2.337
W_{ax}–OW_{eq}	1.911	1.908
W_{ax}–μ_6O	2.415	2.417

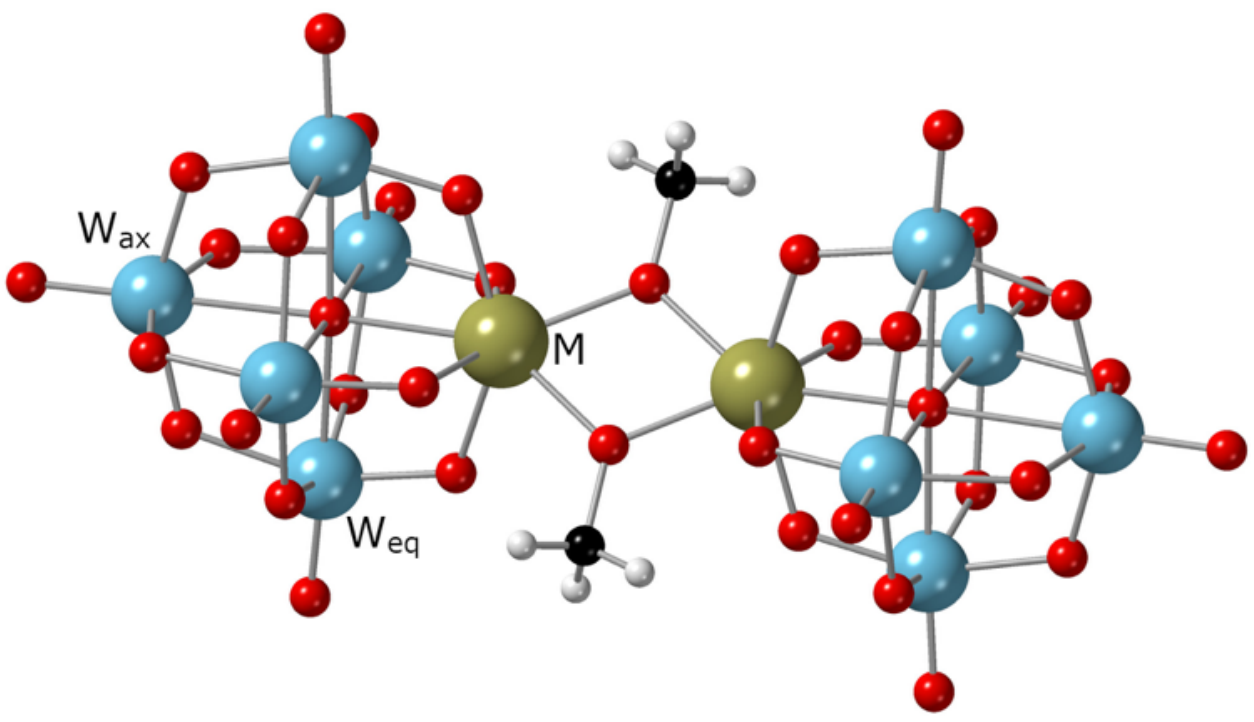

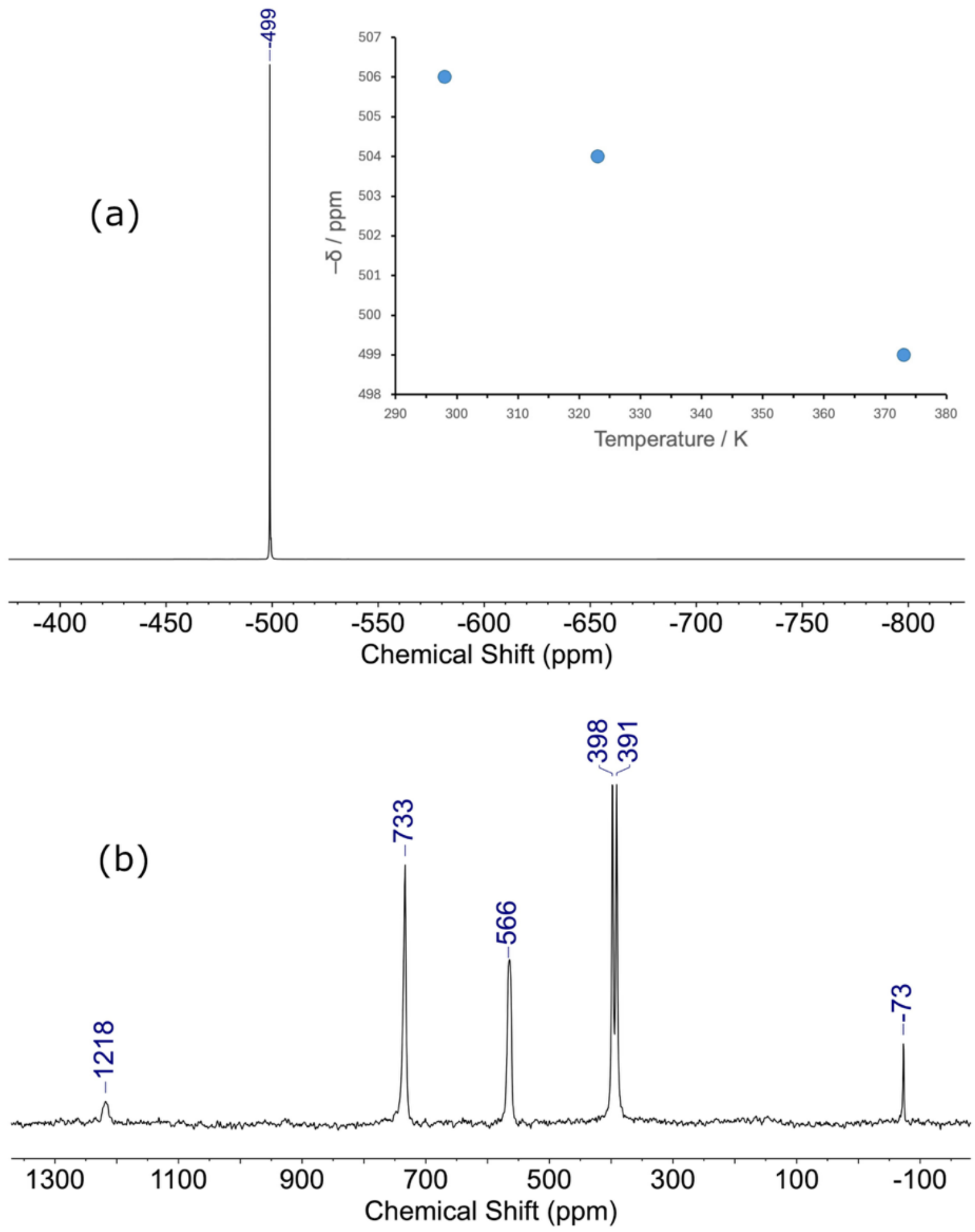

Figure S6. ^{51}V NMR spectrum of the reaction mixture in the synthesis of $(TBA)_3$**7** from base-degraded $(TBA)_2$**1** and V_2O_5 with inset showing the temperature-dependence of the ^{51}V chemical shift (a) and of the ^{17}O NMR spectrum of the precipitated product (b).

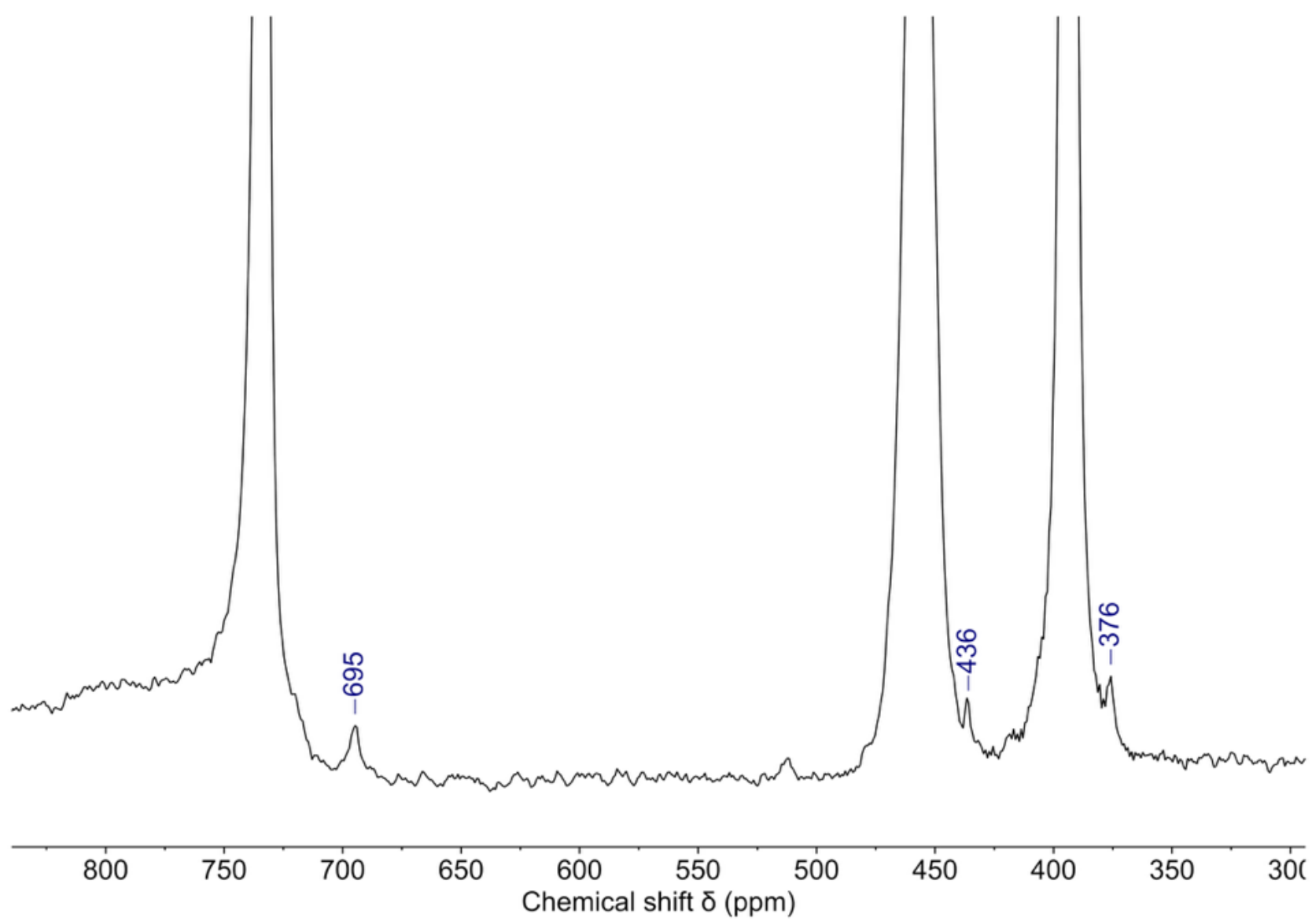

Figure S7. Expansion of ^{17}O NMR spectrum of $(TBA)_8$**8** obtained from base-degraded $(TBA)_2$**1** and $Nb(OEt)_5$ showing impurity peaks at 695, 436 and 376 ppm assigned to $[Nb_2W_4O_{19}]^{4-}$.

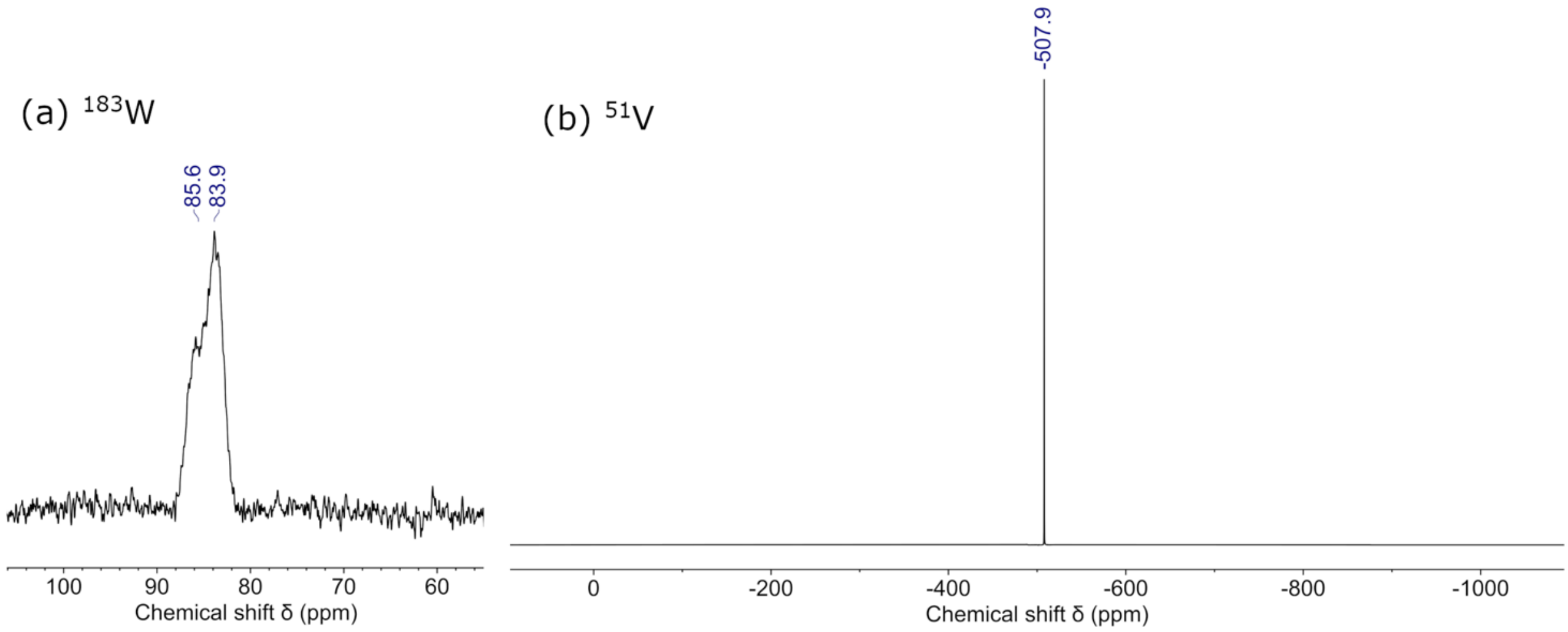

Figure S8. ^{183}W NMR spectrum of the solution obtained by heating a mixture of $WO_3.H_2O$, V_2O_5 and (TBA)OH (10:1:6 mole-ratio) in DMF at 180 °C for 35 minutes, recorded at 100 °C (a) and ^{51}V NMR spectrum of the crystalline product in MeCN (b).

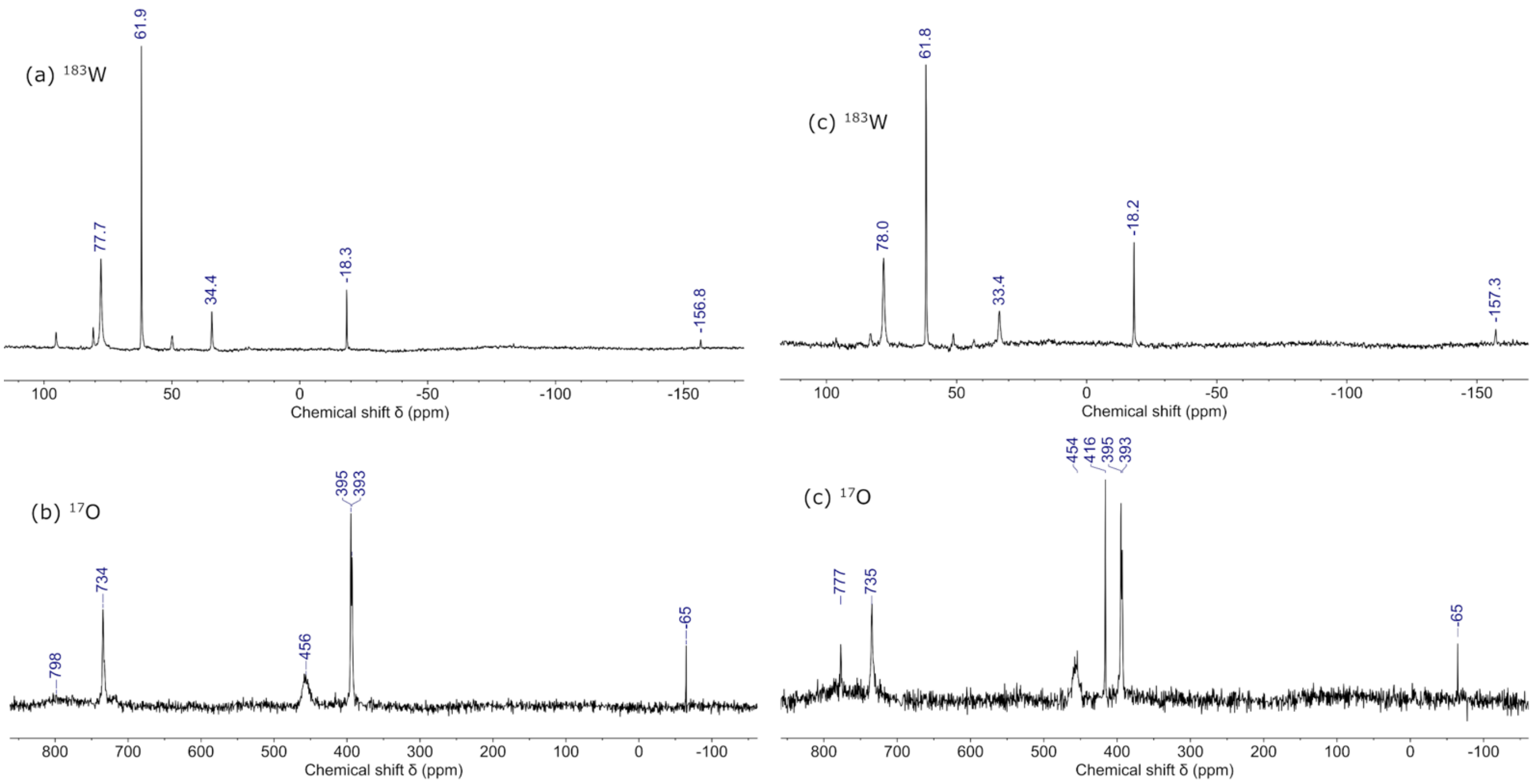

Figure S9. ^{183}W NMR spectrum of the solution obtained by treating the product from the reaction between $WO_3.H_2O$ and (TBA)OH with $Nb(OEt)_5$ (5:3:1 mole-ratio) in DMF at 180 °C for 30 minutes, recorded at 60°C (a), ^{17}O NMR spectrum of crystals obtained upon cooling after heating for 3 hours (b) and ^{183}W and ^{17}O NMR spectra of the solid obtained from the mother liquor by precipitation with diethyl ether (c).

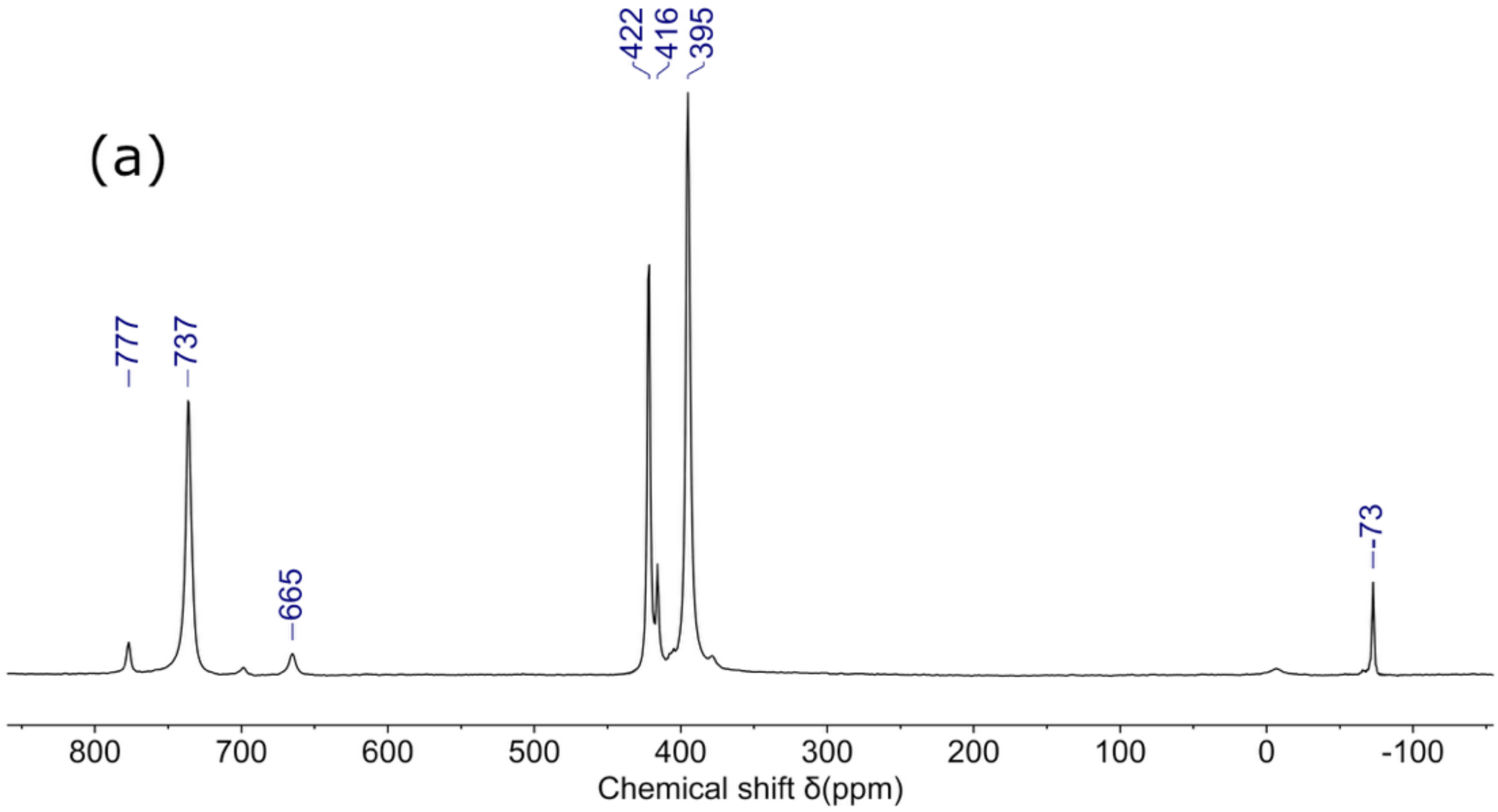

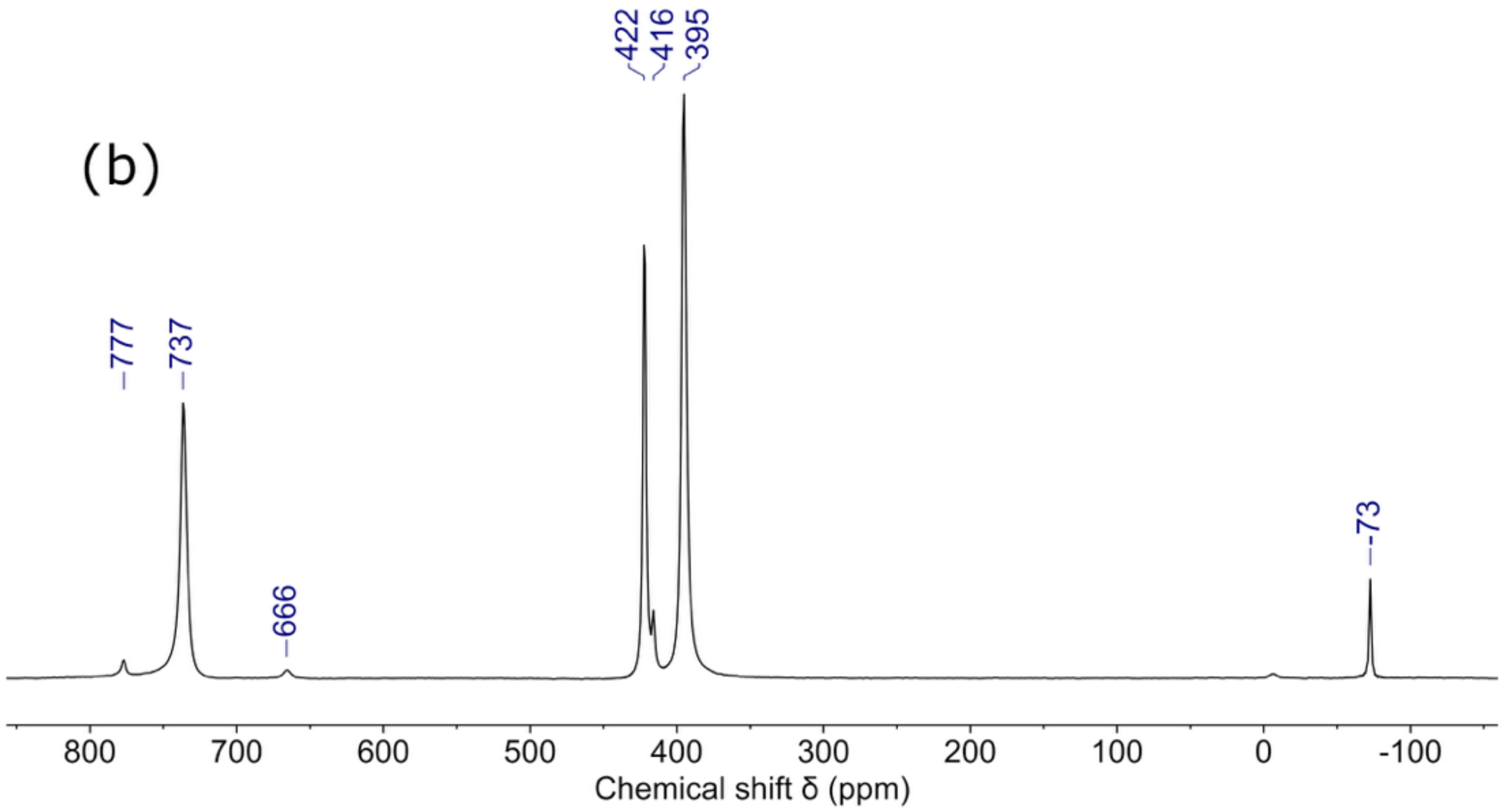

Figure S10. ^{17}O NMR spectra of the product obtained by (i) treatment of $WO_3.H_2O$ with 0.34 mole-equivalents of (TBA)OH in DMF at 180 °C for 10 min, (ii) addition of 0.27 mole-equivalents of methanolic (TBA)OH and 1.29 mole-equivalents of ^{17}O-enriched H_2O, (iii) reaction with 0.22 mole-equivalents of $Ta(OEt)_5$ at 20 °C overnight, (iv) precipitation of the product with diethyl ether (a) and recrystallisation from MeCN (b).

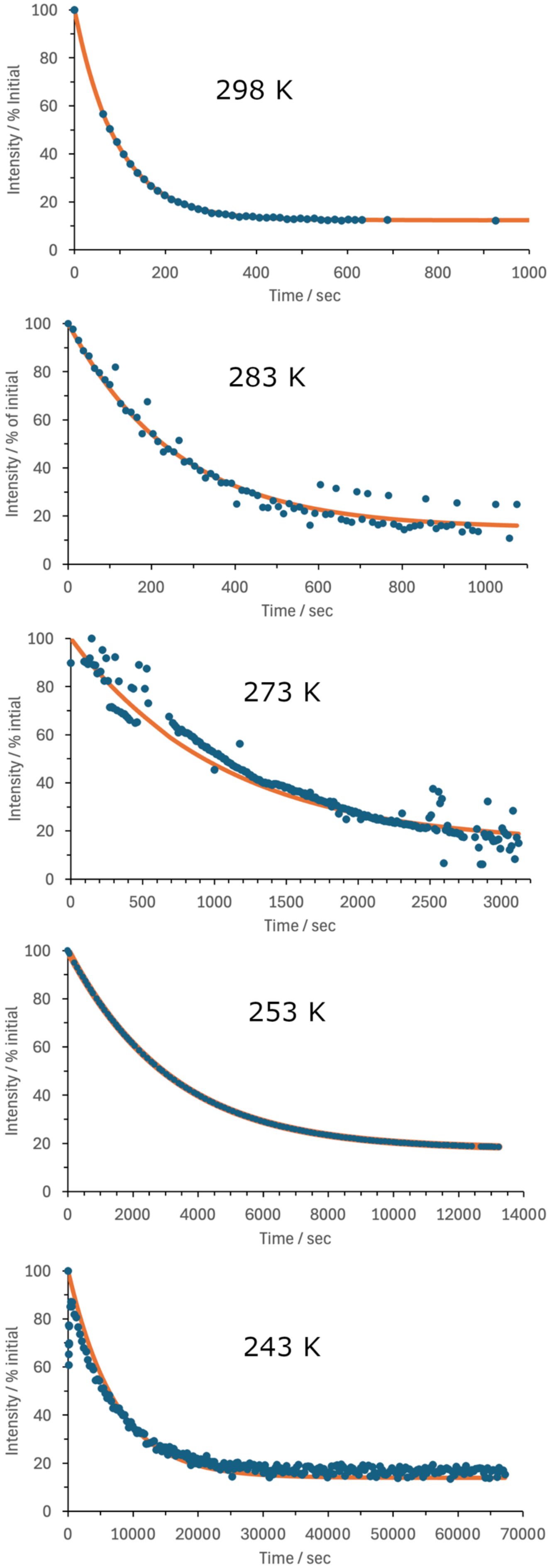

Figure S11. Variable temperature ^{1}H NMR kinetic plots for exchange reactions between $(TBA)_6$**6** and CD_3OD with least squares fits shown as orange lines.

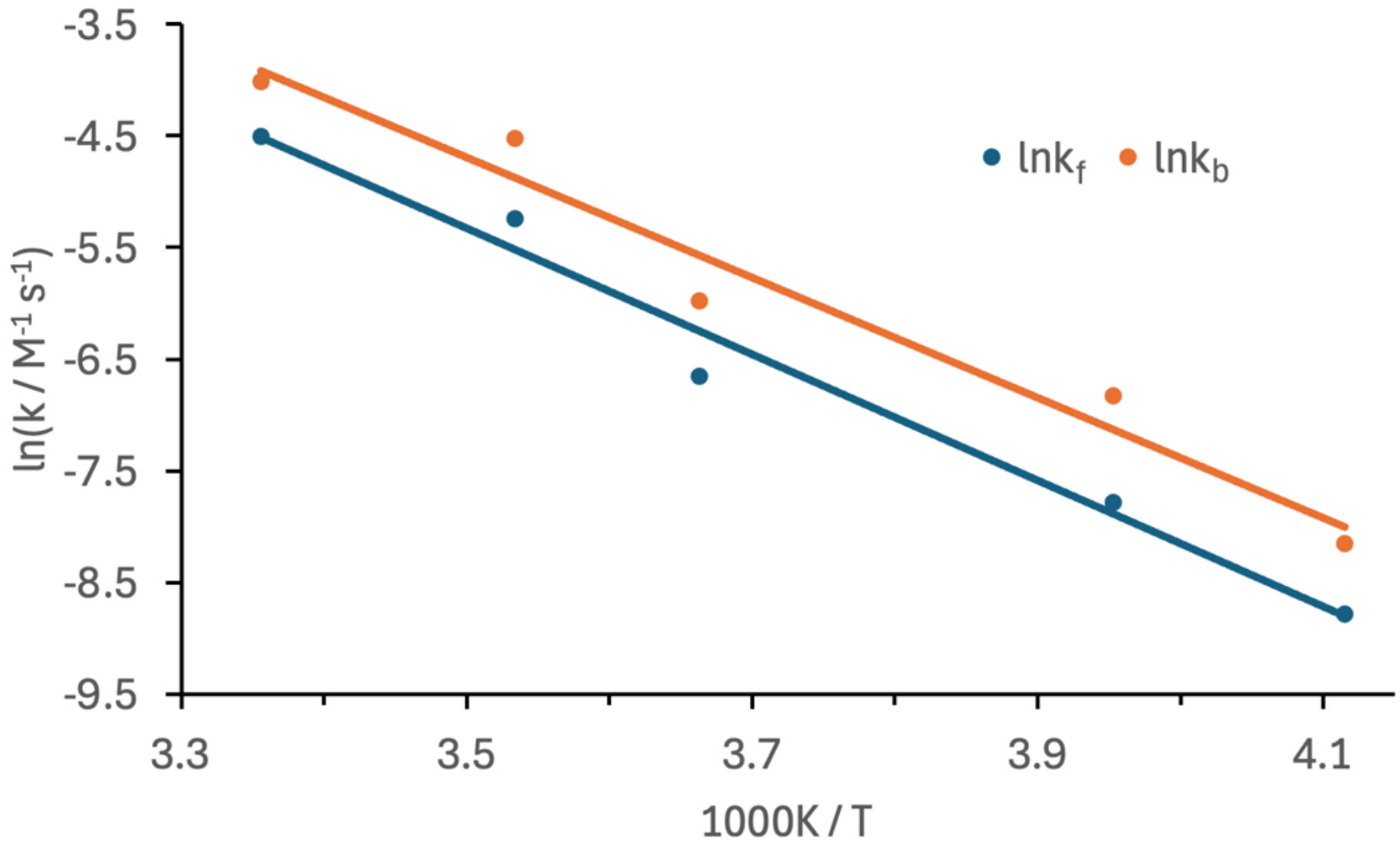

Figure S12. Arrhenius plots obtained from kinetic data in Figure S11 for the forward and backward exchange reactions between $(TBA)_6$**6** and CD_3OD.

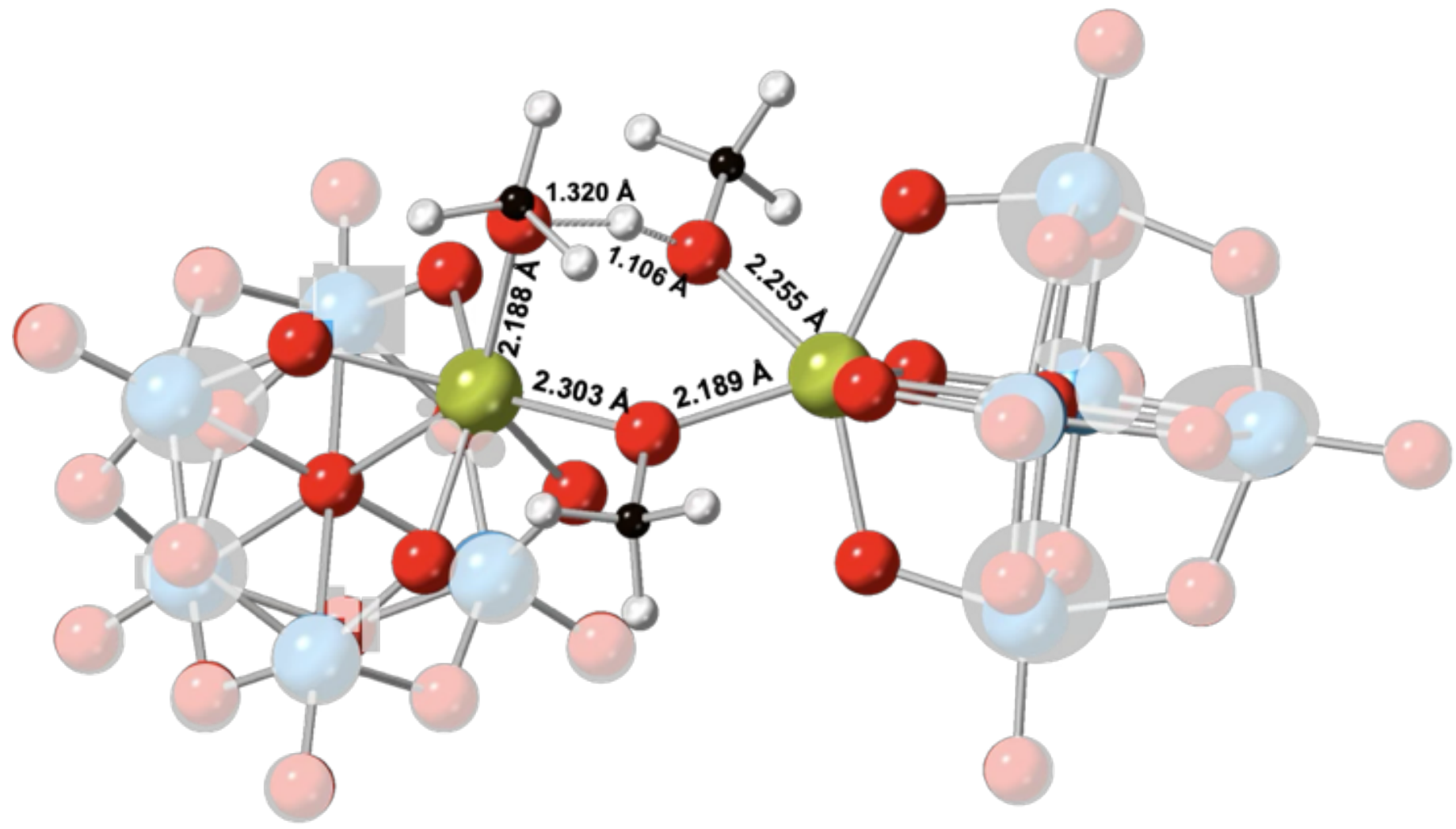

Figure S13. Calculated transition state for exchange reaction between $(TBA)_6$**5** and MeOH, highlighting the coordination environments of the Zr atoms.

Table S3. Comparison of calculated activation energies E_a (kJ mol^{-1}) for hydrolysis of {(MeO)MW_5} Lindqvist anions.

Anion	E_a	Reference
$[(MeO)TiW_5O_{18}]^{3-}$ **3**	110.5	17
$[(MeO)SnW_5O_{18}]^{3-}$ **4**	84.5	17
$[\{(MeO)ZrW_5O_{18}\}_2]^{6-}$ **5**	84.1	this work
$[(MeO)NbW_5O_{18}]^{2-}$	82.8	17

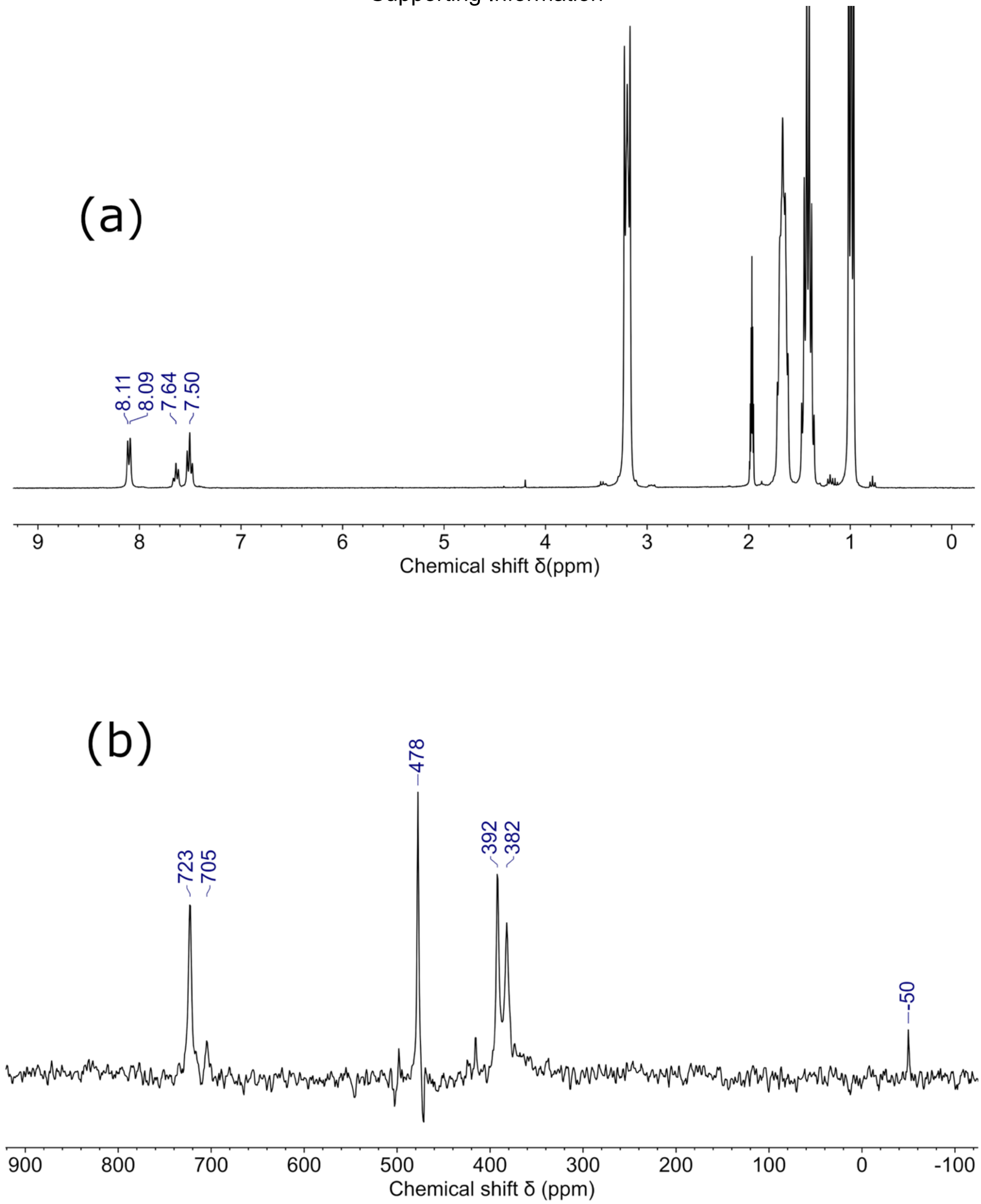

Figure S14. ^{1}H (a) and ^{17}O (b) NMR spectra of $(TBA)_3[(PhCO_2)ZrW_5O_{18}]$ $(TBA)_3$**12**

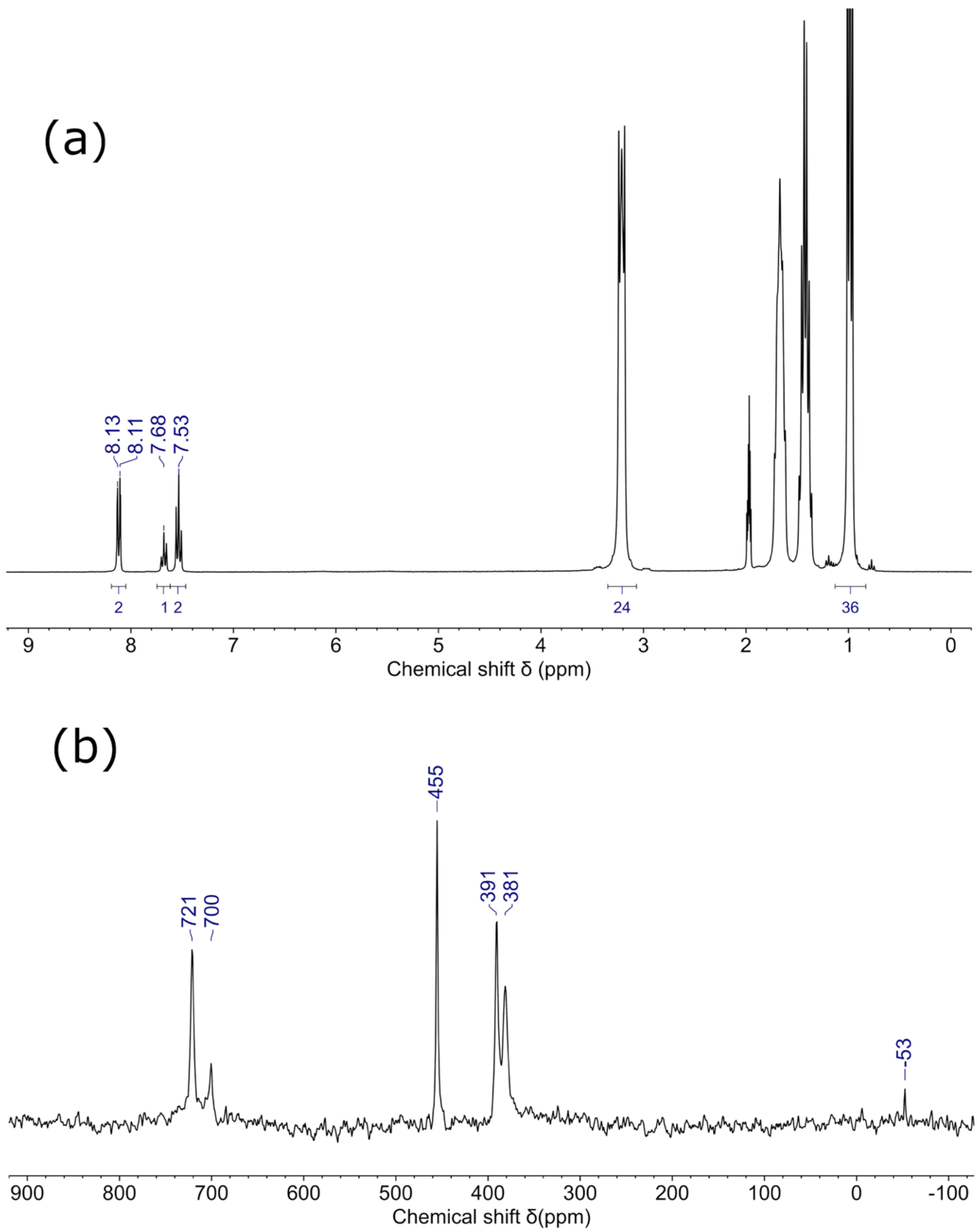

Figure S15. ^{1}H (a) and ^{17}O (b) NMR spectra of $(TBA)_3[(PhCO_2)HfW_5O_{18}]$ $(TBA)_3$**13.**

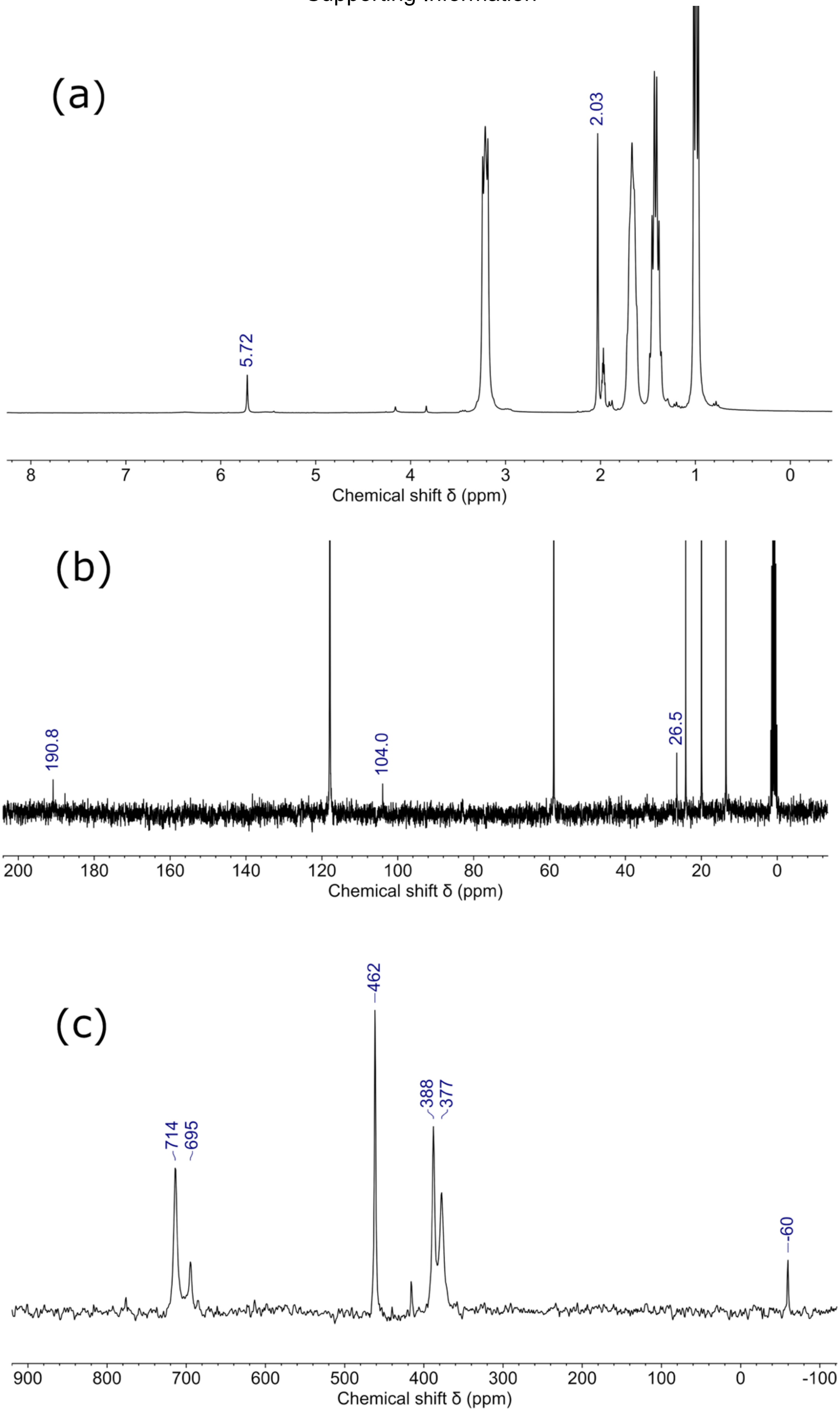

Figure S16. ^{1}H (a), ^{13}C (b) and ^{17}O (c) NMR spectra of $(TBA)_3[(acac)HfW_5O_{18}]$ $(TBA)_3$**14.**

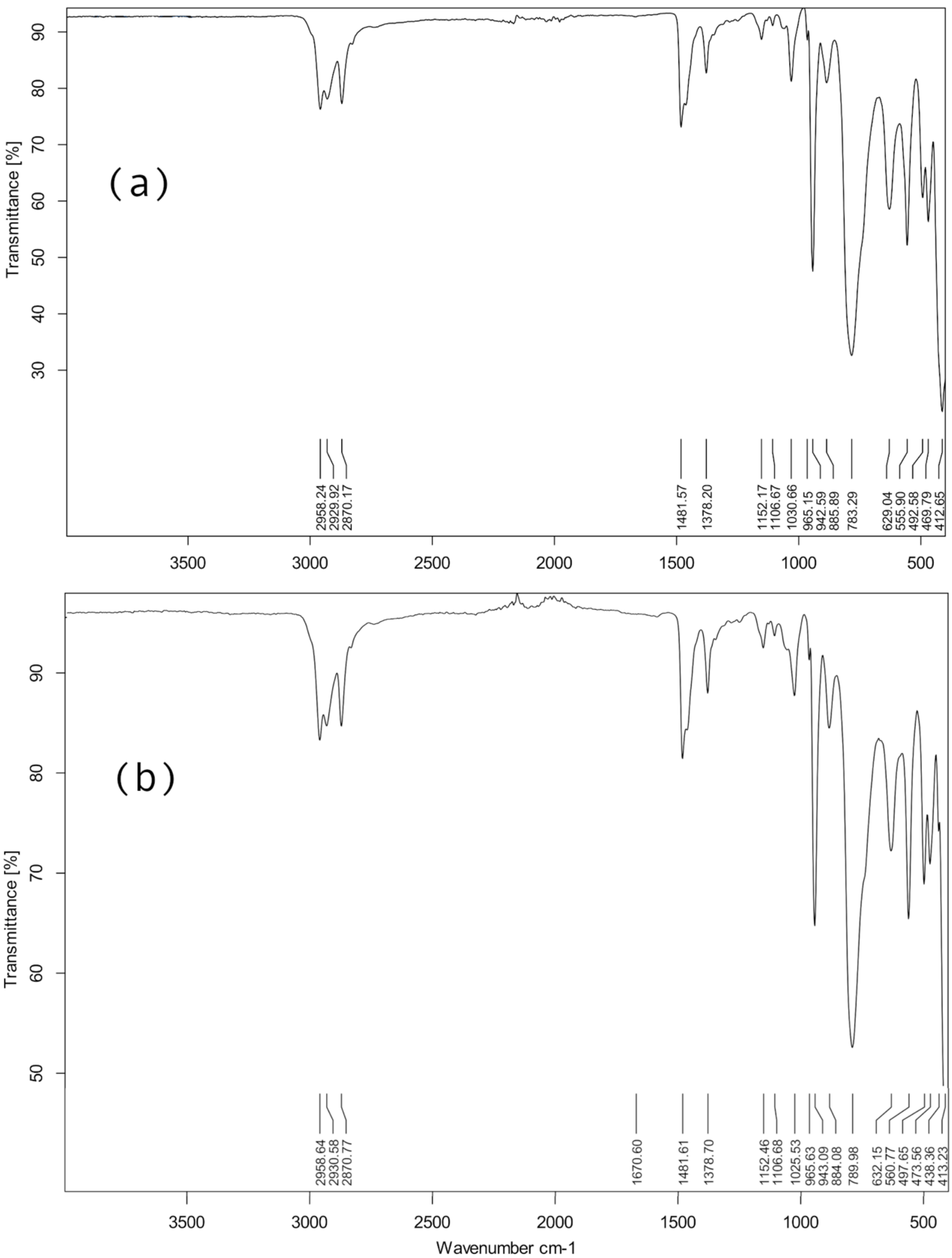

Figure S17. ATR FTIR spectra of $(TBA)_6[\{(MeO)MW_5O_{18}\}_2]$: M = Zr, **5** (a) and Hf, **6** (b).

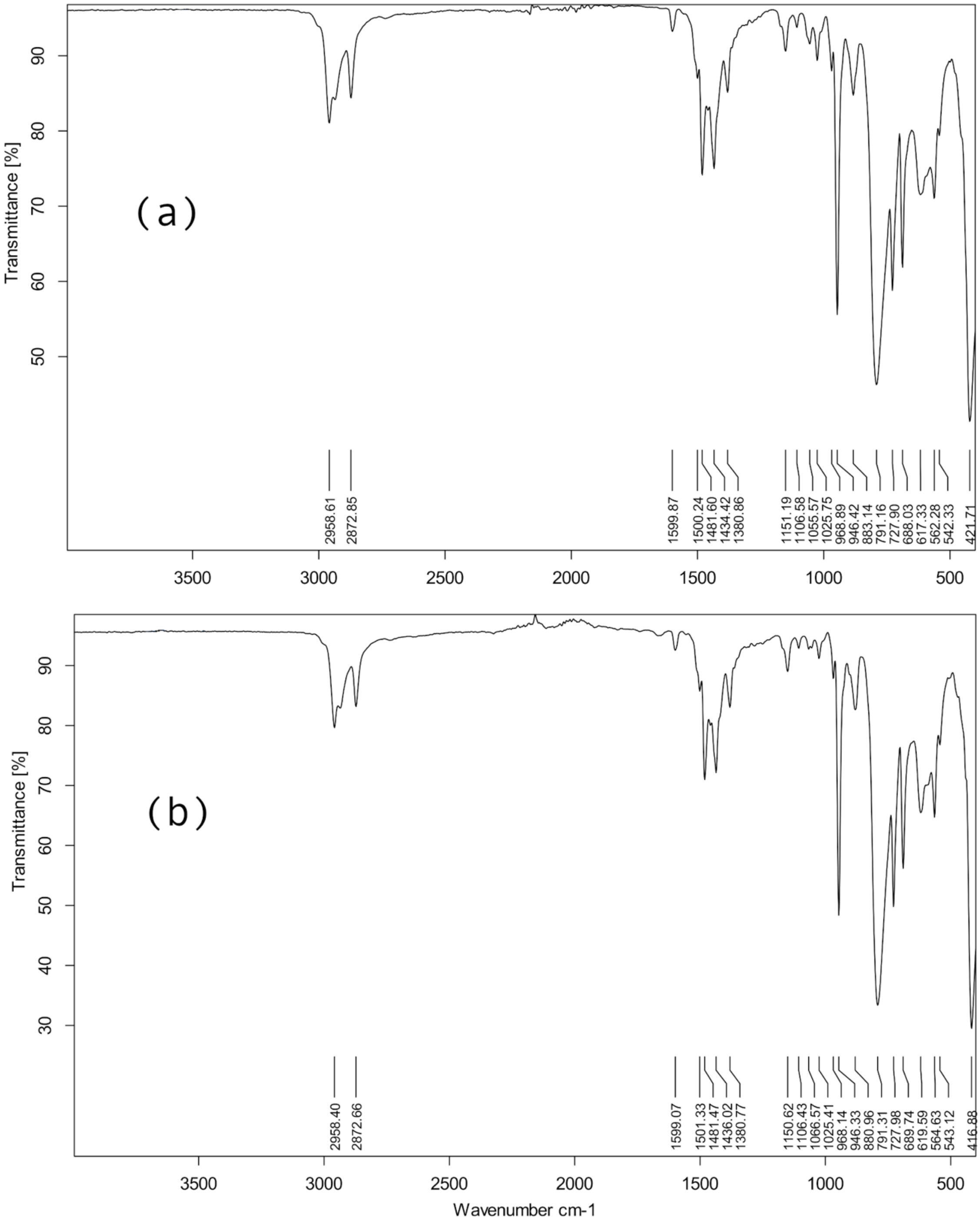

Figure S18. ATR FTIR spectra of $(TBA)_3[(PhCO_2)MW_5O_{18}]$: M = Zr, **12** (a) and Hf, **13** (b).

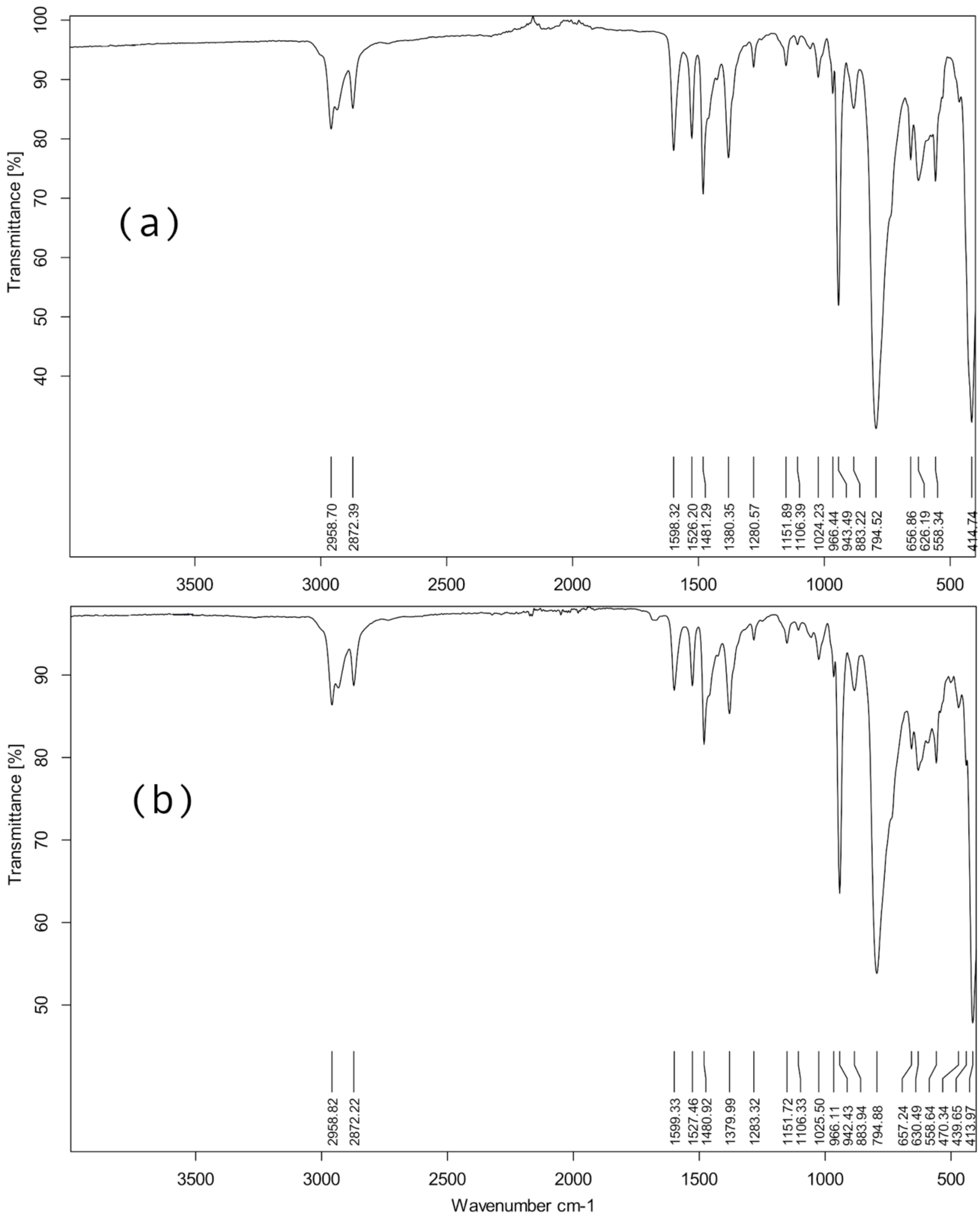

Figure S19. ATR FTIR spectra of $(TBA)_3[(acac)MW_5O_{18}]$: M = Zr, **11** (a) and Hf, **14** (b).

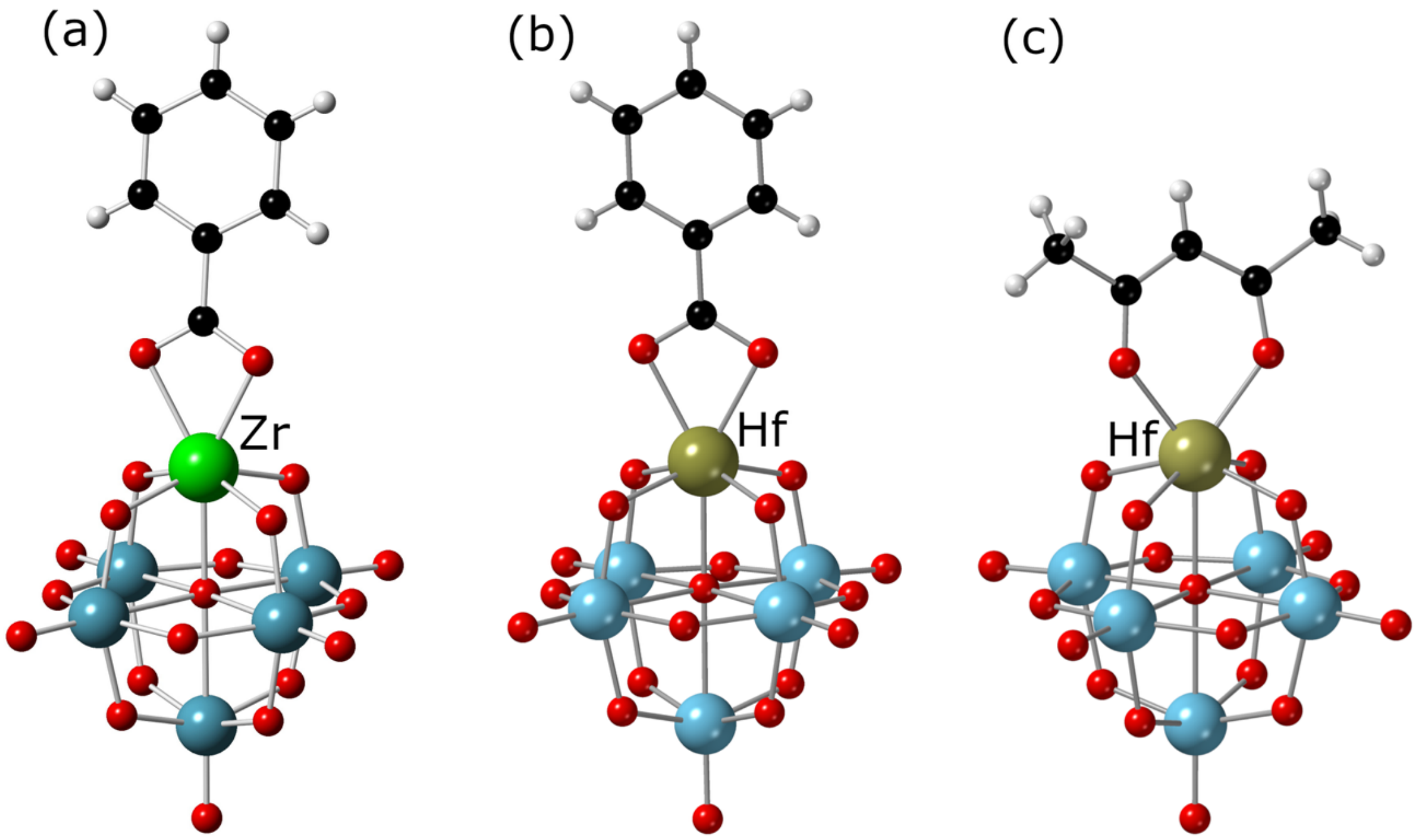

Figure S20. X-ray crystal structures of the anions $[(PhCO_2)ZrW_5O_{18}]^{3-}$ **12** (a), $[(PhCO_2)HfW_5O_{18}]^{3-}$ **13** (b) and $[(acac)HfW_5O_{18}]^{3-}$ **14** (c).

Table S4. Crystallographic parameters for $(TBA)_3$**12**, $(TBA)_3$**13** and $(TBA)_3$**14**.

	$(TBA)_3$**12**	$(TBA)_3$**13**	$(TBA)_3$**14**
Empirical formula	$C_{55}H_{113}N_3O_{20}W_5Zr$	$C_{55}H_{113}HfN_3O_{20}W_5Zr$	$C_{53}H_{102.31}HfN_3O_{20}W_5$
Formula weight	2146.95	2234.22	2199.42
Temperature/K	150.0(2)	150.0(2)	150.0(2)
Crystal system	monoclinic	monoclinic	orthorhombic
Space group	Pc	Pc	$P2_12_12_1$
a/Å	24.8557(2)	24.84140(10)	16.7979(6)
b/Å	17.92514(15)	17.87400(10)	17.9082(5)
c/Å	16.37189(14)	16.42110(10)	24.8816(7)
α/°	90	90	90
β/°	104.0151(8)	104.1190(10)	90
γ/°	90	90	90
Volume/Å^3	7077.22(11)	7070.96(7)	7484.9(4)
Z	4	4	4
ρ_{calc}g/cm^3	2.015	2.099	1.952
μ/mm^{-1}	16.301	17.769	16.777
F(000)	4136.0	4264.0	4173.0
Crystal size/mm^3	0.16 × 0.11 × 0.08	0.13 × 0.12 × 0.03	0.15 × 0.12 × 0.08
Radiation	CuKα (λ = 1.54184)	CuKα (λ = 1.54184)	CuKα (λ = 1.54184)
2Θ range for data collection/°	7.332 to 133.832	4.3944 to 154.75	6.08 to 155.446
Index ranges	-29 ≤ h ≤ 29, -21 ≤ k ≤ 21, -19 ≤ l ≤ 19	-30 ≤ h ≤ 31, -21 ≤ k ≤ 19, -20 ≤ l ≤ 20	-18 ≤ h ≤ 20, -22 ≤ k ≤ 13, -31 ≤ l ≤ 25
Reflections collected	102993	183821	32081
Independent reflections	23085 [R_{int} = 0.0549, R_{sigma} = 0.0474]	27069 [R_{int} = 0.0476, R_{sigma} = 0.0293]	13468 [R_{int} = 0.0415, R_{sigma} = 0.0486]
Data/restraints/parameters	23085/1979/1594	27069/2100/1678	13468/1834/902
Goodness-of-fit on F^2	1.047	1.061	1.050
Final R indexes [I>=2σ (I)]	R_1 = 0.0352, wR_2 = 0.0789	R_1 = 0.0252, wR_2 = 0.0636	R_1 = 0.0558, wR_2 = 0.1492
Final R indexes [all data]	R_1 = 0.0434, wR_2 = 0.0846	R_1 = 0.0258, wR_2 = 0.0640	R_1 = 0.0631, wR_2 = 0.1557
Largest diff. peak/hole / e Å^{-3}	0.95/-1.25	0.96/-1.35	2.23/-2.12
Flack parameter	0.546(14)	0.140(9)	0.010(14)

Table S5. Averaged bond distances (Å) and angles (°) for the monmomeric anions $[(PhCO_2)ZrW_5O_{18}]^{3-}$ **12**, $[(PhCO_2)HfW_5O_{18}]^{3-}$ **13**, $[(acac)ZrW_5O_{18}]^{3-}$ **11** and $[(acac)HfW_5O_{18}]^{3-}$ **14**.

	**12**	**13**	**11** [a]	**14**
M–OC	2.280	2.258	2.195	2.179
M–OW_{eq}	2.074	2.065	2.084	2.078
M–μ_6O	2.314	2.291	2.377	2.376
OMO angle at ligand	56.5	57.2	74.0	74.4
M.....W_{eq}	3.332	3.318	3.366	3.355
W_{eq}–OM	1.851	1.853	1.849	1.828
W_{ax}–OM	2.429	2.432	2.416	2.403
W=O	1.710	1.711	1.712	1.716
W_{eq}–OW_{eq}	1.932	1.934	1.933	1.939
W_{eq}–OW_{ax}	1.962	1.963	1.959	1.957
W_{eq}–μ_6O	2.338	2.338	2.332	2.331
W_{ax}–OW_{eq}	1.912	1.907	1.912	1.913
W_{ax}–μ_6O	2.429	2.432	2.416	2.403

[a] Ref. 18

References

1. R. J. Errington, *Advanced Practical Inorganic and Metalorganic Chemistry*, Blackie Academic and Professional. London, **1997**.

2. Hampden-Smith, M. J.; Wark, T. A.; Rheingold, A. and Huffman, J. C. *Can. J. Chem.* **1991**, *69*, 121-129.

3. Priebsch, W. and Rehder, D. *Inorg. Chem.* **1990**, *29*, 3013-3019.

4. Bradley, D. C.; Mehrotra, R. C. and Wardlaw, W. *J. Chem. Soc.* **1953**, 1634-1636.

5. Clark, R. C. and Reid, J. S. The analytical calculation of absorption in multifaceted crystals. *Acta Crystallogr*. **1995**, *A51*, 887-897.

6. *CrysAlisPro*, Rigaku Oxford Diffraction, Tokyo, Japan.

7. Sheldrick, G. M. SHELXT – Integrated space-group and crystal-structure determination. *Acta Crystallogr., Sect. A: Found. Crystallogr.* **2015**, *71*, 3-8.

8. Sheldrick, G. M. A short history of shelx. *Acta Crystallogr., Sect. A: Found. Crystallogr.* **2008**, *64*, 112-122.

9. Dolomanov, O. V.; Bourhis, L. J.; Gildea, R. J.; Howard, J. A. K. and Puschmann, H. Olex2: a complete structure solution refinement and analysis package. *J. Appl. Crystallogr.* **2009**, *42*, 339-341.

10. Gaussian 09, Frisch, M. J.; Trucks, G. W.; Schlegel, H. B.; Scuseria, G. E.; Robb, M. A.; Cheeseman, J. R.; Scalmani, G.; Barone, V.; Mennucci, B.; Petersson, G. A.; Nakatsuji, H.; Caricato, M.; Li, X.; Hratchian, H. P.; Izmaylov, A. F.; Bloino, J.; Zheng, G.; Sonnenberg, J. L.; Hada, M.; Ehara, M.; Toyota, K.; Fukuda, R.; Hasegawa, J.; Ishida, M.; Nakajima, T.; Honda, Y.; Kitao, O.; Nakai, H.; Vreven, T.; Montgomery Jr., J. A.; Peralta, J. E.; Ogliaro, F.; Bearpark, M.; Heyd, J. J.; Brothers, E.; Kudin, K. N.; Staroverov, V. N.; Kobayashi, R.; Normand, J.; Raghavachari, K.; Rendell, A.; Burant, J. C.; Iyengar, S. S.; Tomasi, J.; Cossi, M.; Rega, N.; Millam, J. M.; Klene, M.; Knox, J. E.; Cross, J. B.; Bakken, V.; Adamo, C.; Jaramillo, J.; Gomperts, R.; Stratmann, R. E.; Yazyev, O.; Austin, A. J.; Cammi, R.; Pomelli, C.; Ochterski, J. W.; Martin, R. L.; Morokuma, K.; Zakrzewski, V. G.; Voth, G. A.; Salvador, P.; Dannenberg, J. J.; Dapprich, S.; Daniels, A. D.; Farkas, Ö.; Foresman, J. B.; Ortiz, J. V.; Cioslowski, J.; Fox, D. J. Gaussian, Inc., Wallingford CT, **2009**.
11. Lee, C.; Yang, W. and Parr, R. G. Development of the Colle-Salvetti correlation-energy formula into a functional of the electron density. *Phys. Rev. B*. **1988**, *37*, 785-789.
12. Stephens, P. J.; Devlin, F. J.; Chabalowski, C. F. and Frisch, M. J. *Ab Initio* calculation of vibrational absorption and circular dichroism spectra using density functional force fields. *J. Phys. Chem.* **1994**, *98*, 11623-11627.
13. Hay, P. J. and Wadt, W. R. *Ab Initio* effective core potentials for molecular calculations. Potentials for K to Au including the outermost core orbitals. *J. Chem. Phys.* **1985**, *82*, 299-310.
14. Miertus, S.; Scrocco, E. and Tomasi, J. Electrostatic interaction of a solute with a continuum. A direct utilization of *ab initio* molecular potentials for the prevision of solvent effects. *Chem. Phys.* **1981**, *55*, 117-129.
15. Vitaly V Pavlishchuk, V. V; Addison W. A., Conversion constants for redox potentials measured versus different reference electrodes in acetonitrile solutions at 25°C, *Inorg. Chim. Acta* **2000**, 298, 97-102.
16. Kelly, C. P.; Cramer, C. J.; Truhlar, D. G. Single-Ion Solvation Free Energies and the Normal Hydrogen Electrode Potential in Methanol, Acetonitrile, and Dimethyl Sulfoxide. *J. Phys. Chem. B* **2007**, 111, 408−422.
17. Lebbie, D.; Izuagie, T.; Pascual-Borràs, M.; Kandasamy, B.; Wills, C.; Waddell, P. G.; Horrocks, B. R. and Errington, R. J. Protonolysis and Condensation Reactions of Alkoxido-Substituted Lindqvist $\{MW_5\}$ and Keggin $\{MPW_{11}\}$ Polyoxometalates: Comparative Experimental and Modeling Studies. *Inorg. Chem*., **2025**, *64*, 2379–2393.
18. Errington, R. J.; Petkar, S. S.; Middleton, P. S.; McFarlane, W.; Clegg, W.; Coxall, R. A. and Harrington, R. W. Synthesis and reactivity of the methoxozirconium pentatungstate $(^nBu_4N)_6[\{(\mu\text{-MeO})ZrW_5O_{18}\}_2]$: insights into proton-transfer reactions, solution dynamics, and assembly of $\{ZrW_5O_{18}\}^{2-}$ building blocks. *J. Am. Chem. Soc*. **2007**, *129*, 12181–12196.
